# Lipid21: Complex Lipid Membrane Simulations with AMBER

**DOI:** 10.1021/acs.jctc.1c01217

**Published:** 2022-02-03

**Authors:** Callum J. Dickson, Ross C. Walker, Ian R. Gould

**Affiliations:** † Computer-Aided Drug Discovery, Global Discovery Chemistry, Novartis Institutes for BioMedical Research, 181 Massachusetts Avenue, Cambridge, Massachusetts 02139, United States; ‡ GlaxoSmithKline PLC, 1250 S. Collegeville Road, Collegeville, Pennsylvania 19426, United States; § Department of Chemistry and Biochemistry, University of California, San Diego, 9500 Gilman Drive, La Jolla, California 92093, United States; ∥ Department of Chemistry, Imperial College London, London, SW7 2AZ, U.K.

## Abstract

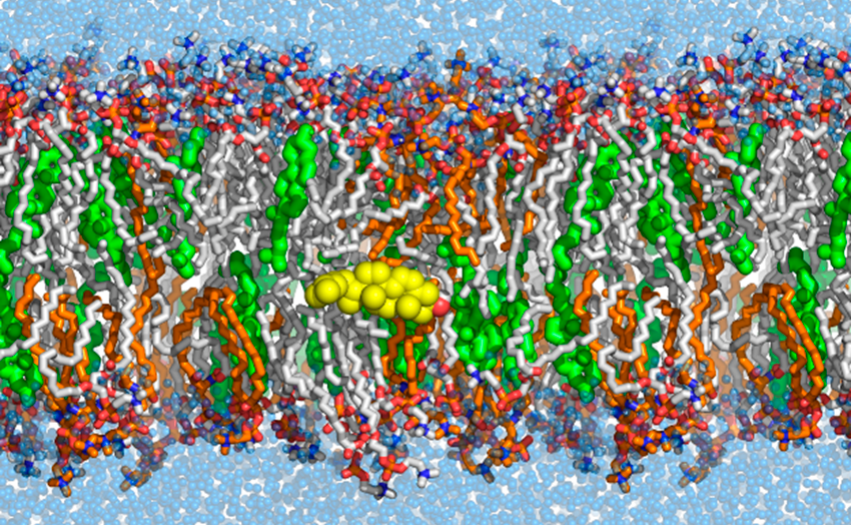

We extend the modular AMBER lipid force field to include anionic lipids, polyunsaturated fatty acid (PUFA) lipids, and sphingomyelin, allowing the simulation of realistic cell membrane lipid compositions, including raft-like domains. Head group torsion parameters are revised, resulting in improved agreement with NMR order parameters, and hydrocarbon chain parameters are updated, providing a better match with phase transition temperature. Extensive validation runs (0.9 μs per lipid type) show good agreement with experimental measurements. Furthermore, the simulation of raft-like bilayers demonstrates the perturbing effect of increasing PUFA concentrations on cholesterol molecules. The force field derivation is consistent with the AMBER philosophy, meaning it can be easily mixed with protein, small molecule, nucleic acid, and carbohydrate force fields.

## Introduction

In recent years, the convergence of lipid membrane force field development and advances in computer hardware have allowed the molecular dynamics simulation of numerous membrane phenomena, such as bilayer phase transitions,^1^ vesicle dynamics,^2^ and the behavior of realistic cell membranes.^3^ Whereas coarse-grained force fields are approaching simulations of membrane patches that are of biologically relevant time scales and dimensions, such simulations are beyond the reach of atomistic force fields in the absence of specialized hardware.^4^ However, atomistic force fields come to the fore when studying processes in atomic detail, such as drug-membrane interactions,^5^ membrane protein behavior,^6^ or membrane-peptide partitioning.^7^ To gain the most relevant insights, atomistic lipid force fields must be parametrized appropriately,^8^ such that agreement to experiment is optimal.

AMBER is both a molecular dynamics software suite and a series of interoperable force fields covering proteins, nucleic acids, carbohydrates and a selection of lipid types. Beginning with Lipid11,^9^ followed by GAFFlipid^10^ and then Lipid14^11^ the AMBER lipid force field has gone through a number of iterations but until recently only supported simulation of phosphatidylcholine (PC), phosphatidylethanolamine (PE), and cholesterol lipids. Although Lipid14 allowed tensionless simulations of bilayers, deficiencies were reported, such as poor agreement with NMR headgroup order parameters,^12^ a mismatch of the DPPC phase transition temperature, and the observation of gel phase behavior of DMPC at long simulation times.^13^

In this work, we update the AMBER lipid force field to address these issues, while also extending the coverage of lipid species. Due to the modular nature of the force field, parameter derivation is only required for individual headgroup and tail units. Head group torsion parameters are simultaneously fitted to QM conformational energies. Partial charges are derived for all lipid head groups. Hydrocarbon chain parameters are updated, allowing better agreement with phase transition temperatures. We run multiple long validation simulations for each lipid species and find suitable agreement with biophysical measurements. Finally, model lipid raft simulations are performed to investigate the impact of increasing PUFA concentrations on cholesterol molecules.

We also document instances when lipid simulations with AMBER give undesired results—simulations of anionic lipids are found to be sensitive to the cation type and force field model. We recommend best practices for lipid simulations with AMBER, which now cover a range of lipid types with the modular Lipid21 force field.

## Methods

The initial parameter set comprises lipid tail van der Waals parameters derived in the fitting of Lipid14 to reproduce densities and heats of vaporization of long chain hydrocarbons, and lipid tail partial charges, also derived in the fitting of Lipid14.^11a^ The remaining parameters were initially covered by GAFF version 1.7 and the AMBER phosphate parameters.^14^

**Figure 1:**
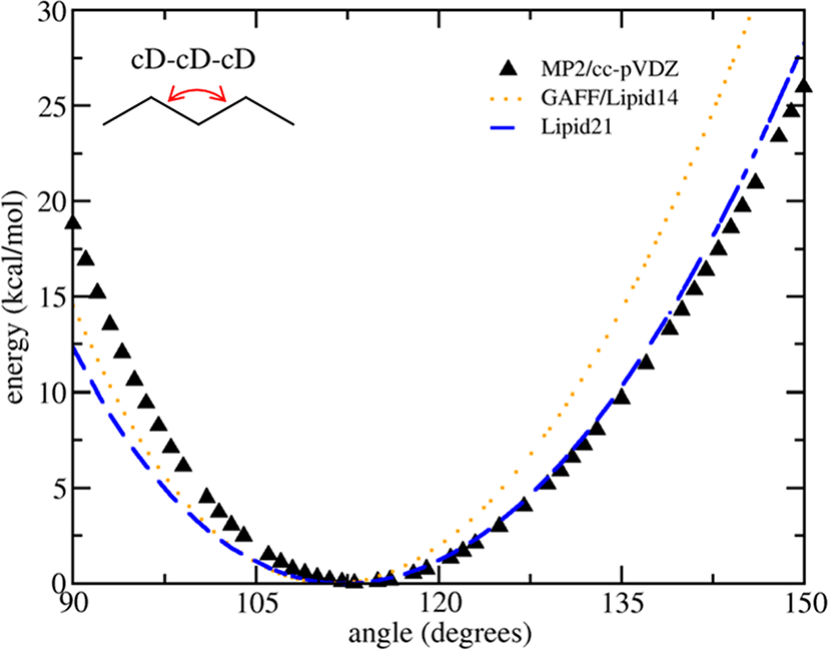
Fit of the C–C–C angle parameter to a MP2/cc-pVTZ scan on a pentane molecule.

**Figure 2:**
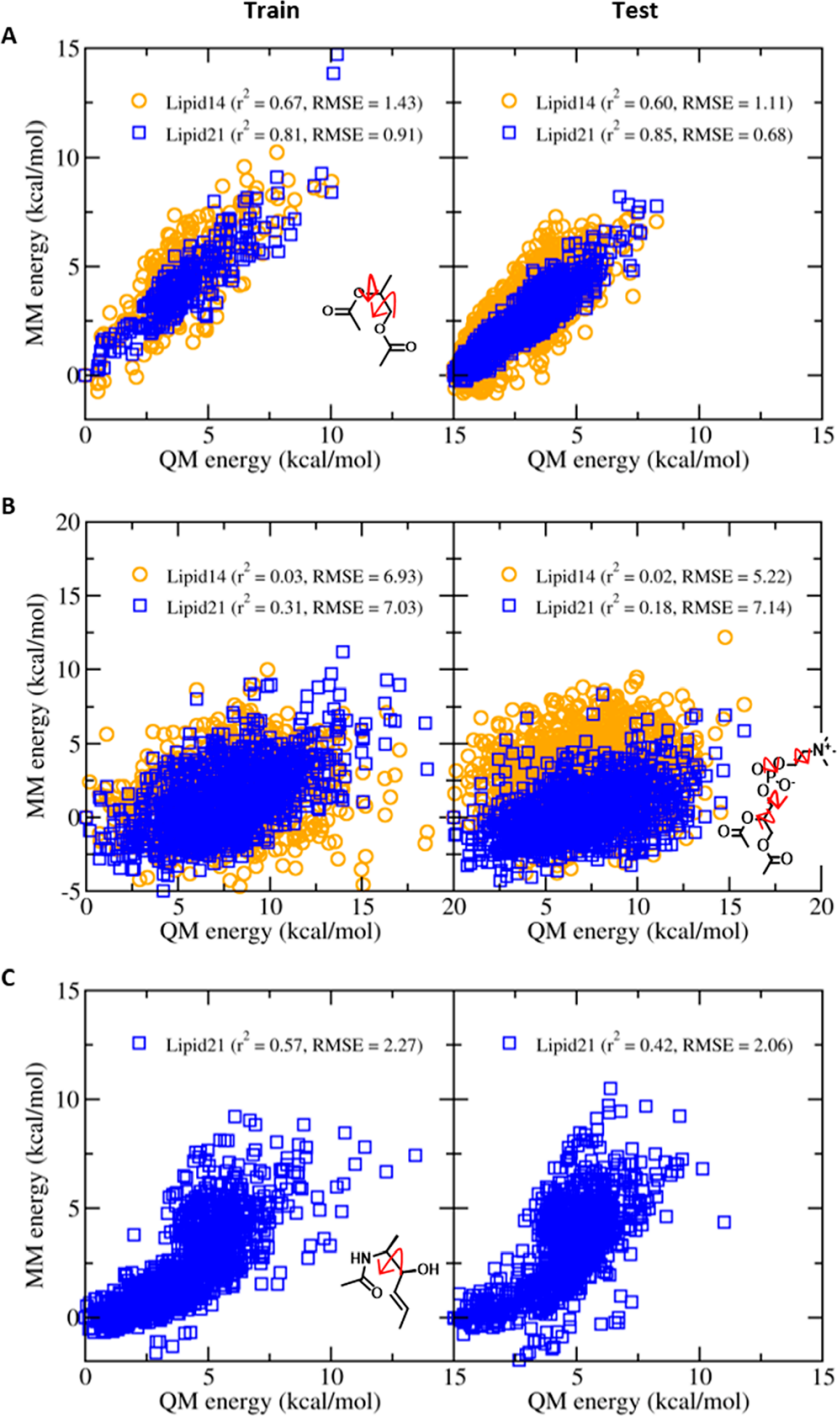
(a) Fitting of cC–oS–cA–cA and oS–cA–cA–oS torsions to QM single-point energies (MP2/cc-pVDZ) using model glyceride compounds; (b) fitting of cA–cA–cA–oT, cA–cA–oT–pA, cA–oT–pA–oT, and oT–cA–cA–nA torsions to QM single-point energies (MP2/cc-pVDZ + PCM) using model phosphatidylcholine compounds; and (c) fitting cB–cA–cA–nN torsion using model ceramide compounds using a training set (left) and performance on an equally sized test set (right).

A summary of all parameters fitted in this work is as follows: the alkane C–C–C angle in lipid tails with atom types cD–cD–cD was found to have a large influence on bilayer fluidity and was fit to quantum mechanical energies (see Figure 1). To accommodate this fit, all hydrocarbon torsions in lipid tails were refit (atom types cD–cD–cD–cD, cD–cB–cB–cD, cB–cD–cD–cD, cB–cB–cD–cD, cB–cB–cD–cB) (see Supporting Information (SI) Figures S3–S7). All headgroup partial charges are derived at the MP2/cc-pVTZ level with the polarizable continuum model using the Lipid11 capping strategy,^9^ covering phosphatidylcholine (PC), phosphatidylglycerol (PG), phosphatidylserine (PS), phosphatidylethanolamine (PE), phosphatidic acid (PH−), and sphingomyelin (SPM) head groups. Lipid tail partial charges are adopted from Lipid14.^11a^ Finally, headgroup torsion terms are fit to quantum mechanical energies (atom types cC–oS–cA–cA, oS–cA–cA–oS, cA–cA–cA–oT, cA–cA–oT–pA, cA–oT–pA–oT, oT–cA–cA–nA, cB–cA–cA–nN) (see Figure 2).

### Quantum Mechanical Energies

All QM single-point energies, angle scans, and torsion scans were performed using Gaussian 09.^15^ Angle and torsion scans on model hydrocarbon molecules were performed at the MP2/cc-pVTZ level. Angles were scanned from 90 to 150° in 1° increments. Torsions were scanned over 360° in 10° increments. Single-point energy calculations on model glyceride, ceramide, and phosphatidylcholine molecules were performed at the MP2/cc-pVDZ level, with application of the polarizable continuum model to create an implicit solvent environment for phosphatidylcholine only. To obtain relevant glyceride, ceramide, and phosphatidylcholine conformations, POPC and PSM simulations were performed for 100 ns using initial Lipid21 parameters, and 1000 random lipid structures were extracted. Prior to the QM single-point energy calculation, each molecule was minimized using AMBER20^16^ for 1000 steps, with the first 500 steps using the steepest descent and the final 500 steps using the conjugate gradient method,^17^ with restraints of 5000 kcal/mol/rad on each of the torsions being fitted. The GBn generalized Born model (igb = 7)^18^ was used during minimization of model phosphatidylcholine molecules only. We repeated this process on separate trajectories to create a test set, used for parameter validation. Parameters for the model glyceride, ceramide, and phosphatidylcholine molecules consisted of initial Lipid21 parameters and partial charges derived at the MP2/cc-pVDZ level after optimization of a single molecular conformation, allowing a two-stage RESP fit.^19^

### Torsion Parameter Fitting

All torsion parameters were fitted to the QM energies using the Pyevolve Genetic Algorithm.^20^ First, conformational energies were evaluated using AMBER20 with the torsional barrier heights set to zero for torsions of interest. When fitting to a 1D torsion scan, the model fragment was minimized for a small number of steps for each point along the scan, with a restraint applied to the torsion under study.

The Genetic Algorithm was then used to identify torsion barrier height and phase shift parameters which minimize the least-squares fitness function between QM and MM energies:
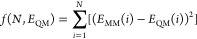
where *N* is the number of conformations, *E*_QM_ is the reference energy from QM and *E*_MM_ is the MM energy from AMBER, calculated as the “zeroed” torsion(s) plus the torsion energy contribution using fitted torsion parameters determined via the standard AMBER torsion equation:
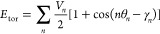
where the sum runs over periodicity *n*, *V*_*n*_ is the barrier height, θ_*n*_ is the torsion angle and γ_*n*_ is the phase shift. When fitting 1D torsion scans, the periodicity *n* = 1–5. When fitting single torsions to collections of glyceride and ceramide conformations, *n* = 1–3. When simultaneously fitting phosphatidylcholine torsions, periodicity was retained from the starting torsion parameters, with the exception of the cA–cA–oT–pA and cA–cA–cA–oT torsions, to which additional period terms were introduced. The phase shift γ_*n*_ was allowed to take terms 0 or 180° during fitting of hydrocarbon torsions. During fitting of headgroup torsions, phase shift γ_*n*_ was allowed to vary in 60° increments to accommodate headgroup stereochemistry.

### Lipid Partial Charge Derivation

Partial charge fitting was performed for all lipid headgroup units using the Lipid11 capping strategy,^9^ at a higher level of QM theory. For each headgroup type, a 100 ns bilayer simulation was performed using Lipid21 fitted parameters, and 200 random lipids were extracted. The methyl headgroup capping procedure was applied as in Lipid11, and a single-point QM calculation was performed at MP2/cc-pVTZ with the polarizable continuum model. Partial charges were then derived for each of the head groups using a two-stage RESP fit; final charges are the average of the 200 individual fits. This procedure provides Boltzmann-averaged implicitly polarized charges over a conformational ensemble.^21^

Due to the different level of QM theory, methyl acetate capping group partial charges were rederived at the MP2/cc-pVTZ level with the polarizable continuum model; updated partial charges are available in the SI Figure S1. The capping procedure used for fitting of sphingomyelin charges is also available in the SI Figure S2.

### DPPC Melting Point Simulations

To prepare a starting structure, a DPPC bilayer was equilibrated at 300 K for 300 ns. The final coordinates were then used for repeat heating simulations, run in the NPT ensemble for 100 ns while increasing the temperature from 300 to 350 K, at a rate of 0.5 K/ns. Three independent heating runs were performed. The area per lipid was monitored for each simulation to obtain an estimate of the phase transition temperature.

### Lipid Bilayer Systems

Twenty homogeneous lipid bilayer systems were simulated to validate the structural properties of the membranes, covering zwitterionic, anionic, PUFA, and sphingomyelin lipid species (see Table 1). Initial coordinates were obtained from the CHARMM-GUI.^22^ To ensure relaxation of each bilayer, a simulated annealing protocol was applied to the initial coordinates prior to equilibration and production runs. Specifically, bilayers were heated to 393 K in a 1 ns NPT simulation. Each membrane was then simulated at 393 K for 10 ns in the NPT ensemble. Systems were then cooled to the target temperature (Table 1) in a 1 ns NPT run and equilibrated for 100 ns. Following equilibration, three independent production runs of 300 ns were performed for each lipid species.

**Table 1:** Homogeneous Lipid Bilayer Systems Simulated in This Work

lipid type	temp (K)	no. lipids	no. waters	counterions	simulation time (ns)
DLPC	303	128	5120		3 × 300
DMPC	303	128	5120		3 × 300
DPPC	323	128	5120		3 × 300
DSPC	333	128	5120		3 × 300
DOPC	303	128	5120		3 × 300
POPC	303	128	5120		3 × 300
POPE	310	128	5120		3 × 300
DLPG	303	128	5120	128 K+	3 × 300
DMPG	303	128	5120	128 K+	3 × 300
DPPG	323	128	5120	128 K+	3 × 300
DSPG	333	128	6400	128 K+	3 × 300
DOPG	303	128	5120	128 K+	3 × 300
POPG	303	128	5120	128 K+	3 × 300
DOPS	303	128	5120	128 K+	3 × 300
POPS	298	128	5120	128 K+	3 × 300
POPA	310	128	5120	128 K+	3 × 300
DAPC	303	128	5120		3 × 300
SDPC	297	128	5120		3 × 300
PSM	328	128	5120		3 × 300
SSM	328	128	5120		3 × 300

### Molecular Dynamics Simulations

All simulations performed in this work used a similar protocol. First, each system was minimized for 10000 steps, with the first 5000 steps using steepest descent and the final 5000 steps using the conjugate gradient method.^17^ Heating to 100 K was performed in a 5 ps NVT simulation with restraints of 5 kcal/mol/Å^2^ on nonsolvent molecules. The same restraints were maintained during a 100 ps NPT simulation and systems heated to the target temperature (Table 1). All restraints were removed and the PME was used to treat all electrostatic interactions with a real space cutoff of 10 Å;^23^ a long-range analytical dispersion correction was applied to the energy and pressure. A constant pressure of 1 atm was maintained using a semi-isotropic Berendsen barostat for lipid systems (*x*- and *y*- box vectors were coupled, with the *z*-vector allowed to change freely) and a pressure relaxation time of 1 ps.^24^ Temperature was controlled by the Langevin thermostat,^25^ with a collision frequency of γ = 1 ps^–1^. Water was modeled using the TIP3P water model^26^ and counterions using the Åqvist parameters.^27^ All simulations used AMBER20 PMEMD CUDA on GPU cards using the SPFP precision model.^16,28^ Single exploratory production runs of 300 ns were also executed with the Monte Carlo barostat for pressure coupling, keeping all other settings identical.^29^ Concerning single 1 μs simulations: settings were identical to those described, with the exception that hydrogen mass repartitioning was used to allow a 4 fs time step.^30^

### Raft-like Bilayer Simulations

Three systems with a raft-like composition were prepared and simulated, with increasing mole fraction of the PUFA lipid DAPC (see Table 2). These underwent heating and equilibration of 100 ns as previously described, followed by two repeat 1 μs production runs at 310 K and application of hydrogen mass repartitioning to allow a 4 fs time step.^30^

**Table 2:** Lipid Composition of Raft-like Systems Studied in This Work, with Increasing Mole Fraction of the PUFA Lipid DAPC

χ_DAPC_	*N*_POPC_	*N*_CHOL_	*N*_PSM_	*N*_DAPC_
0	120	40	40	0
0.30	60	40	40	60
0.60	0	40	40	120

### Analysis Protocols

The area per lipid of each system was calculated directly from the simulation box size as done previously.^10^ Volume per lipid was determined in a similar manner, using the simulation box size and the average volume of a TIP3P water at the target temperature over a 50 ns NPT simulation. Bilayer thicknesses were determined from electron density profiles calculated with CPPTRAJ.^31^ NMR order parameters were also calculated with CPPTRAJ. X-ray and neutron scattering form factors were determined with the SIMtoEXP program.^32^ To study the cholesterol tilt angle distribution in the raft-like simulations, the *z*-position of the oxygen atom of each cholesterol was extracted using CPPTRAJ, as was the *z*-position position of the sterol carbon connecting the acyl chain of the cholesterol (see SI Figure S8). The angle between the bilayer normal and this vector was then calculated. The *z*-coordinate of cholesterol oxygen atoms was monitored for transit events, with any visit to −2 < *z* < 2 Å from the bilayer center being counted as an event.

## Parameter Derivation

### Hydrocarbon Parameters

The C–C–C angle parameter was found to have an influence on bilayer fluidity (Lipid21 atom types cD–cD–cD). Therefore, we refitted this angle parameter to high level gas-phase QM scans. The updated angle parameters are seen to better capture the energy minima about 112° and the parabolic energy well at higher angle stretches (see Figure 1).

To accommodate the updated C–C–C angle parameter, hydrocarbon torsions were also refit at the same level of QM theory—these fits are detailed in the SI.

### Glyceride, Ceramide, and Phosphatidylcholine Conformational Energies

Glycerol, ceramide, and phosphatidylcholine headgroup torsions were refit to better reproduce QM single-point energies (see Figure 2). To obtain relevant conformations, 1000 lipids were randomly extracted from 100 ns runs of POPC and PSM bilayers. Each molecule was then minimized using AMBER with restraints on the torsion(s) of interest. The QM energy was then evaluated at the MP2/cc-pVDZ level (with the PCM model applied to phosphatidylcholine molecules only, to mimic the solvent environment lipid head groups experience). Torsions of interest were then fitted simultaneously using the Genetic Algorithm protocol detailed in the Methods section. Regarding the quality of the torsion fitting, glycerol energies could be corrected to reproduce QM energies well, as seen from both the training and test sets. The phosphatidylcholine results are only a marginal improvement, with little change in RMSE, although there is a shift in correlation from *r*^2^ = 0.03 to *r*^2^ = 0.31 on the training set and *r*^2^ = 0.02 to *r*^2^ = 0.18 on the test set, potentially due to the complex electrostatic nature of these zwitter-ionic model molecules. Although it was possible to obtain better reproduction of QM energies by introducing additional periodic terms to each torsion, initial bilayer simulations were poor, indicating the more complex torsion sets suffered from overfitting. Finally, sphingomyelin was not covered previously; however, the torsion fit results indicate that the ceramide torsion reproduces the QM conformational energy landscape well. It was found that this torsion fitting procedure not only improved the bulk phase properties of lipid bilayer simulations but also headgroup order parameter agreement with NMR measurements, similar to recent Slipids results.^33^

## Results

### Bulk Structural Properties

As can be seen from Table 3, simulations with Lipid21 capture well the bulk phase properties of different lipid species as determined by experiment. The performance of PC lipid types is similar to Lipid14, while the area per lipid of POPE comes into good agreement with the experimental result, which was previously under-predicted with Lipid14.^11a^ Regarding the charged lipids, areas per lipid for all PG and PS lipid types agree well with experiment. However, area per lipid for both DSPG and DSPC are slightly low, suggesting that long acyl chains cause slight condensing of membranes with Lipid21.

**Table 3:** Lipid Bilayer Structural Properties from Experiment (exp) and Simulations (sim) with Lipid21. Values Are the Average ± st dev over Three Independent 300 ns Simulations

		area per lipid (Å^2^)		volume per lipid (Å^3^)		bilayer thickness *D*_HH_ (Å)	
lipid type	temp (K)	exp	sim	exp	sim	exp	sim
DLPC	303	63.2,^34^ 60.8^35^	61.14 ± 0.12	991^34^	933.23 ± 0.06	30.8^34^	31.42 ± 0.12
DMPC	303	60.6,^34^ 59.9^35^	59.71 ± 0.13	1101^36,34^	1039.32 ± 0.14	34.4,^37^ 35.3^38^	35.75 ± 0
DPPC	323	63.1,^35^ 64.3^39^	61.69 ± 0.09	1232^36^	1163.50 ± 0.21	38,^40^ 38.3^36^	38.75 ± 0
DSPC	333	63.8^35^	59.66 ± 0.17		1290.99 ± 0.03		43.67 ± 0.12
DOPC	303	67.4,^40^ 72.5^36^	66.95 ± 0.20	1303^36^	1232.84 ± 0.07	35.3,^41^ 36.7,^40,42^ 36.9,^36^ 37.1^43^	38.00 ± 0.41
POPC	303	64.3,^35^ 68.3^44^	63.92 ± 0.09	1256^44^	1190.32 ± 0.13	37^44^	38.50 ± 0.20
POPE	310	56.6,^45^ 59–60^46^	55.92 ± 0.15	1180^46^	1129.94 ± 0.26	39.5^46^	41.50 ± 0.20
DLPG	303	65.6^47^	65.80 ± 0.07	954^47^	927.78 ± 0.06		29.33 ± 0.31
DMPG	303	65.1^47^	65.25 ± 0.22	1057^47^	1034.47 ± 0.19		32.42 ± 0.24
DPPG	323	67.0^47^	67.72 ± 0.13	1189^47^	1159.95 ± 0.07		34.83 ± 0.12
DSPG	333	68.3^47^	66.65 ± 0.04	1305^47^	1293.80 ± 0.14		39.00 ± 0.00
DOPG	303	70.8^47^	71.08 ± 0.10	1265^47^	1227.37 ± 0.07		35.58 ± 0.31
POPG	303	66.1^47^	68.23 ± 0.07	1209^47^	1185.09 ± 0.04		35.92 ± 0.12
DOPS	303	64.1^48^	65.47 ± 0.16	1228^48^	1206.28 ± 0.18	39.0^48^	39.42 ± 0.24
POPS	298	62.7^49^	61.78 ± 0.34		1154.36 ± 0.31	42.2^49^	40.17 ± 0.42
POPA	310		63.90 ± 0.04		1093.10 ± 0.01		37.50 ± 0
DAPC	303		71.81 ± 0.08		1258.34 ± 0.03		36.42 ± 0.12
SDPC	297	68.2^50^	65.10 ± 0.28		1271.79 ± 0.08	37.9^50^	40.33 ± 0.24
PSM	328	61.9^51^	58.50 ± 0.07	1161.7^51^	1120.78 ± 0.27	37.8^51^	39.08 ± 0.12
SSM	328	62.5^51^	57.10 ± 0.20	1226.8^51^	1173.44 ± 0.25	40^51^	41.42 ± 0.24

Simulations of PUFA lipids (SDPC, DAPC) are close to available experimental data for area per lipid and bilayer thickness of SDPC; the SDPC area per lipid is approximately 3 Å^2^ below experiment. The area per lipid of DAPC is similar to the Charmm36 parametrization results.^52^ Finally, the results for sphingomyelin are encouraging, although the area per lipid is 4–5 Å^2^ below recently determined experimental values, suggesting there is room for further improvement.^51^

All production simulations used the Berendsen barostat for pressure coupling.^24^ We also ran single exploratory simulations of 300 ns per lipid type using the Monte Carlo barostat,^29^ which allows a speed-up in simulation throughput, keeping all other simulation settings identical. As shown in Table S1, we find that areas per lipid are depressed for all lipid types, dropping by an average of 3.78 Å^2^. We therefore recommend the Berendsen barostat pressure coupling for lipid bilayer simulations in AMBER.

In the next sections, lipid ordering is analyzed, providing an additional comparison to experiment.

### Lipid Ordering

One of the motivations to reparameterize the PC lipid model was poor agreement of headgroup order parameters with available NMR data, since the headgroup order parameters provide an accurate experimental picture of bilayer structure.^12^

**Figure 3:**
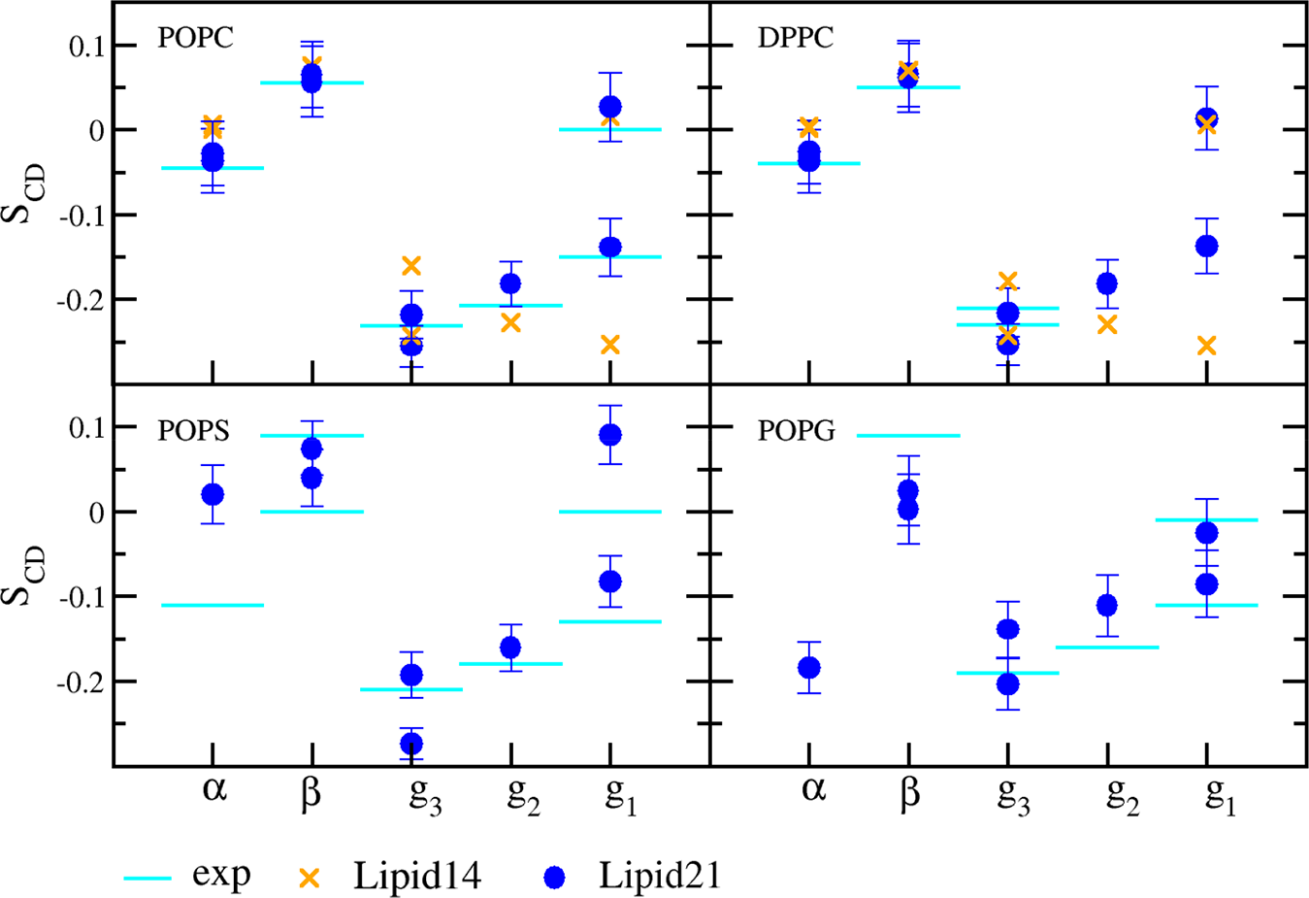
Head group NMR order parameters from experiment and Lipid21 simulations for POPC,^53^ DPPC,^54^ POPS,^55^ and POPG.^56^ Values are the average over a single 300 ns simulation ± st dev. Simulation values for Lipid14 are shown as orange crosses (POPC, DPPC).

Calculated headgroup order parameters for POPC, DPPC, POPS, and POPG from Lipid21 simulations are shown in Figure 3, along with comparison to experiment and in the case of POPC and DPPC, results for previous Lipid14 simulations. Where experimental data are available, POPC and DPPC results agree very well with experiment, showing a marked improvement over Lipid14. Concerning the charged lipids POPS and POPG, headgroup order parameters trend with experiment yet leave room for improvement. In particular, order parameters of the α C–H vector in POPS are significantly far from experiment. However, the overall results indicate that bilayer structure agrees suitably with the NMR experiments.

**Figure 4:**
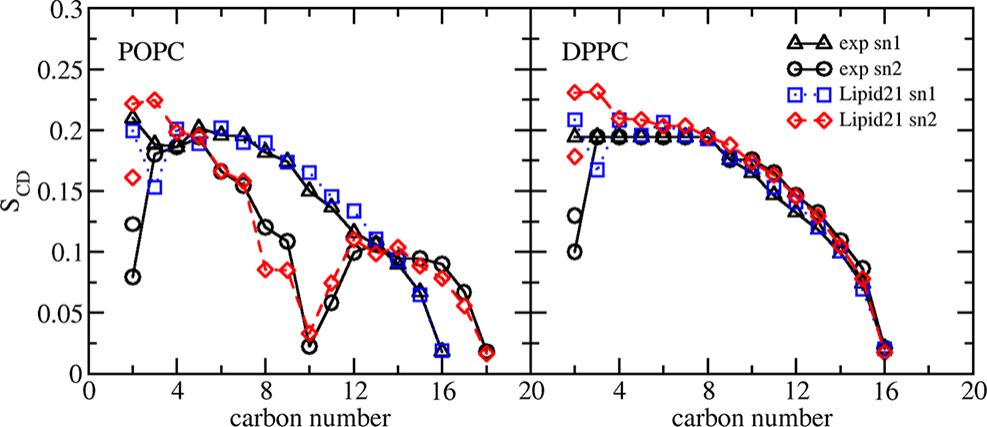
Tail group NMR order parameters from Lipid21 simulations and comparison to experiment for POPC^53^ and DPPC.^57^ Values are the average over a single 300 ns simulation. Error bars are not shown for clarity.

The ordering of the lipid chains can also be calculated allowing comparison to NMR experiments. Figure 4 details the results for POPC and DPPC lipids, finding good agreement with experiment, although the splitting of the sn-2 carbon-2 position does not reach the level determined experimentally and the POPC and DPPC carbon chains are too ordered near the headgroup region.

**Figure 5:**
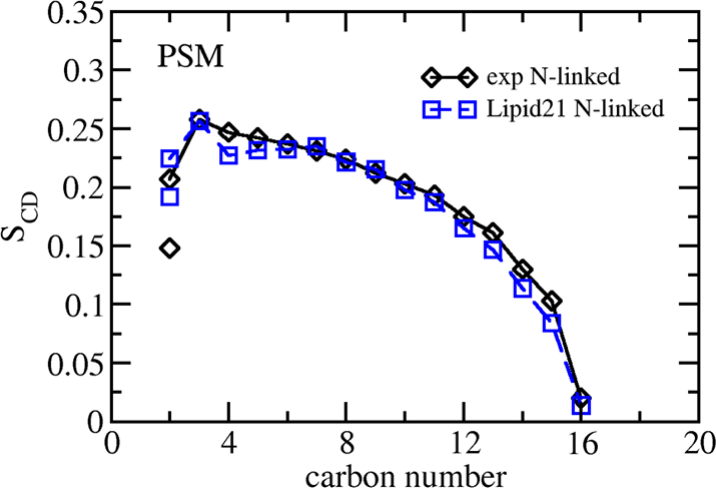
Tail group NMR order parameters from Lipid21 simulations and comparison to experiment for N-linked chain of PSM (16:0 sphingomyelin).^51^

A similar analysis can be performed for the PSM sphingomyelin model, for which NMR data are available for the N-linked chain.^51^Figure 5 details the Lipid21 results, finding good agreement with experiment for the N-linked chain, with the only exception being the carbon-2 position, which does not show the level of splitting observed in NMR—a similar behavior to the Lipid21 POPC and DPPC models. However, the suitable agreement indicates the bilayer structure of PSM is close to that studied in NMR experiments.

**Figure 6:**
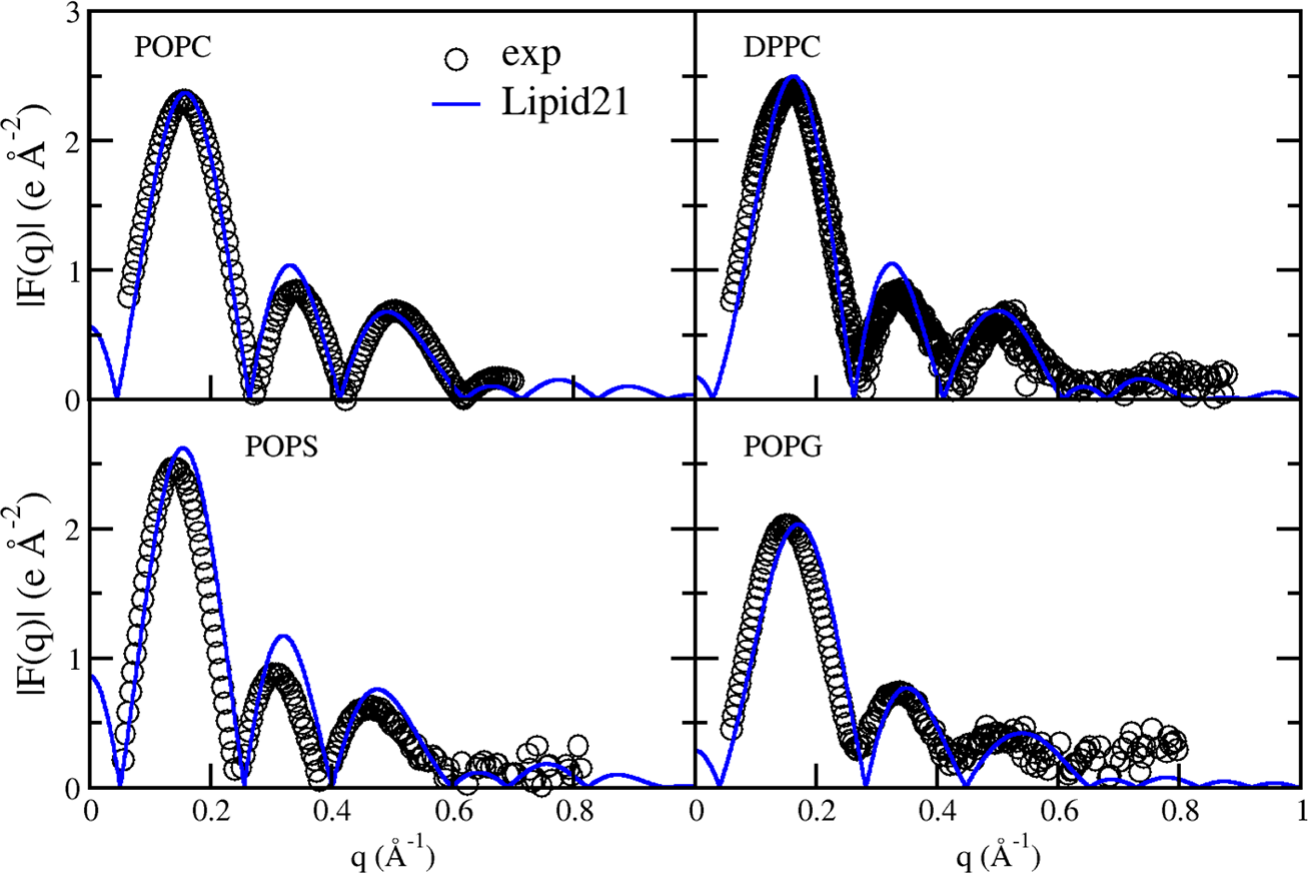
Small-angle X-ray scattering form factors from experiment (open black circles) and Lipid21 simulations (blue line) for POPC,^58^ DPPC,^35,40^ POPS,^32^ and POPG.^47,59^ Profiles are calculated with SIMtoEXP from average atom densities over a single 300 ns simulation.

Finally, small-angle X-ray scattering form factors (SAXS) provide another detailed reference of lipid bilayer structure. The results for POPC, DPPC, POPS, and POPG simulated with Lipid21 and comparison to experimental data are given in Figure 6. For all models, agreement with experiment is suitable, in particular the positions of the minima. A mismatch between experimental and simulated SAXS profiles is however observed around the second maxima, being particularly pronounced for POPS.

**Figure 7:**
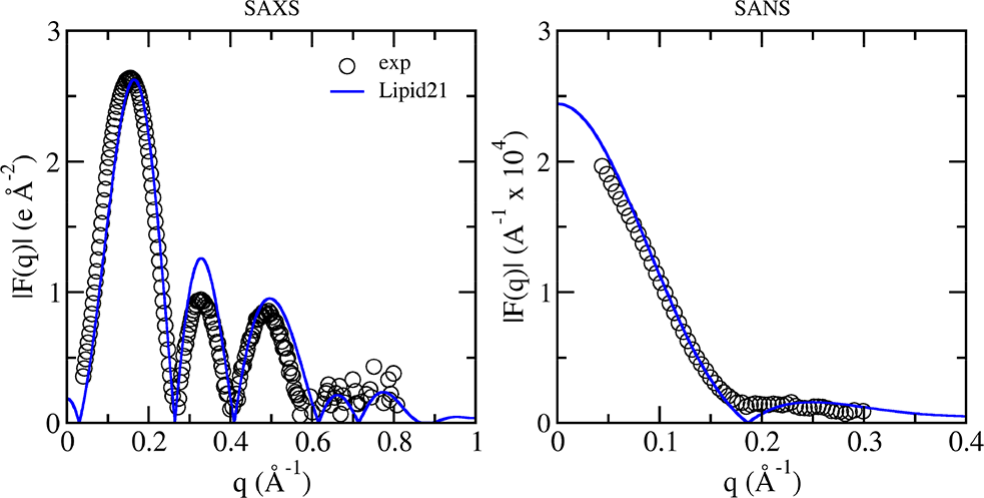
(Left) Small-angle X-ray scattering and (right) small-angle neutron scattering profiles for PSM from experiment at 100% D_2_O and simulation with Lipid21.^51^ The experimental data were collected at 318 K and simulation was performed at 328 K.

Also shown are the SAXS and small-angle neutron scattering (SANS) form factors for PSM collected at 318 K and 100% D_2_O,^51^ with comparison to Lipid21 simulations (328 K) in Figure 7. Both scattering profiles compare well to experiment, with the SAXS results showing minima at equivalent positions as experiment. As with the POPS result, the second SAXS maxima is overpredicted in comparison to experiment.

### DPPC Melting Point

**Figure 8:**
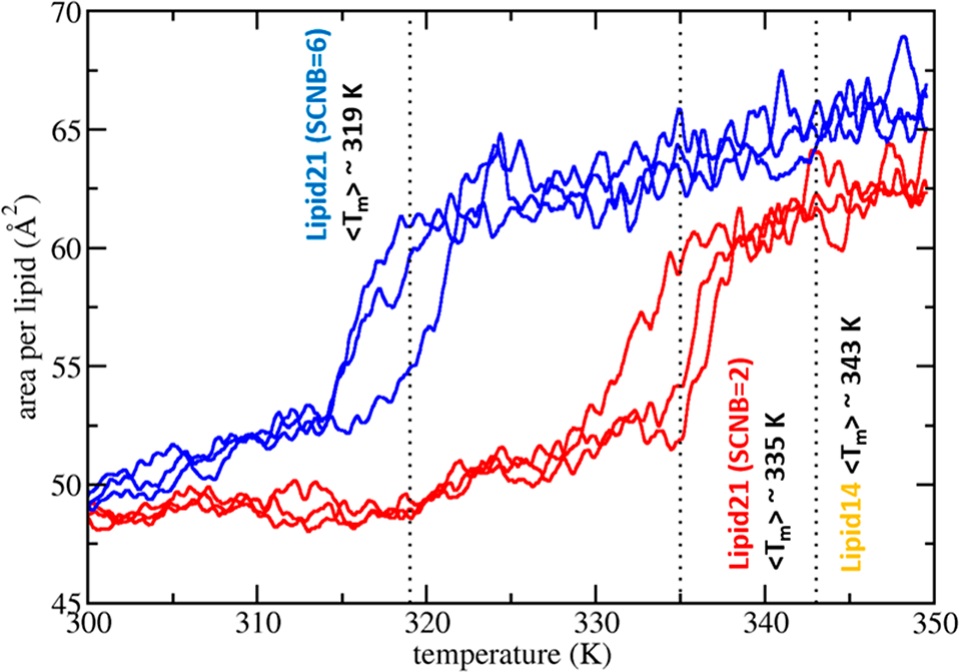
Melting point scan results for DPPC with Lipid21 parameters and 1–4 Lennard-Jones scaling factors 0.5 (SCNB = 2) or 0.167 (SCNB = 6) on the cD–cD–cD–cD torsion. Results are presented as the running average of the area per lipid, with a window size of 1 ns, as a function of temperature. The experimental melting point for DPPC is 314 K.^60^

Following updating of hydrocarbon bonded parameters, fitting of headgroup torsions to single-point QM energies and partial charge derivation, the initial Lipid21 parameter set was tested for the ability to reproduce the DPPC experimental melting point (see Figure 8). A lipid force field comparison from Pluháčková et al. indicated that the Lipid14 DPPC melting point is too high, estimating a value of 343 K, far above the experimental DPPC melting point of 314 K.^13,60^ Melting point scans were performed with a similar methodology using the initial Lipid21 model, in which bilayers are gradually heated from 300 to 350 K, at a rate of 0.5 K/ns. Plots of the area per lipid as a function of temperature show an inflection point at approximately 335 K for the initial Lipid21 parameter set. In order to better tune the force field to bilayer melting points, we scale down the 1–4 Lennard-Jones interaction of the acyl chain torsion parameter cD–cD–cD–cD, from the standard AMBER setting of 0.5 scaling to 0.167 (via the SCNB setting). By default, the Amber force field scales down 1–4 nonbonded terms to reduce the exaggeration of short-term repulsion caused by the Lennard-Jones potential (to model van der Waals interactions) and Coulomb potential (to model electrostatic interactions). The 1–4 scaling factor was previously proposed as a parameter by which to tune the melting point and other liquid phase properties of N-alkanes.^61^ The Lennard-Jones 1–4 scaling of 0.167 was found to bring the DPPC melting point down to 319 K, much closer to the experimental value of 314 K. As such, this scaling factor, which is included automatically when building the topology file, is incorporated into the final Lipid21 parameter set. It should be noted that this bilayer heating method is expected to overestimate the true melting point temperature by 5–10 K.^62^

### Influence of Cation Type and Force Field Model on Anionic Lipid Simulations

**Figure 9:**
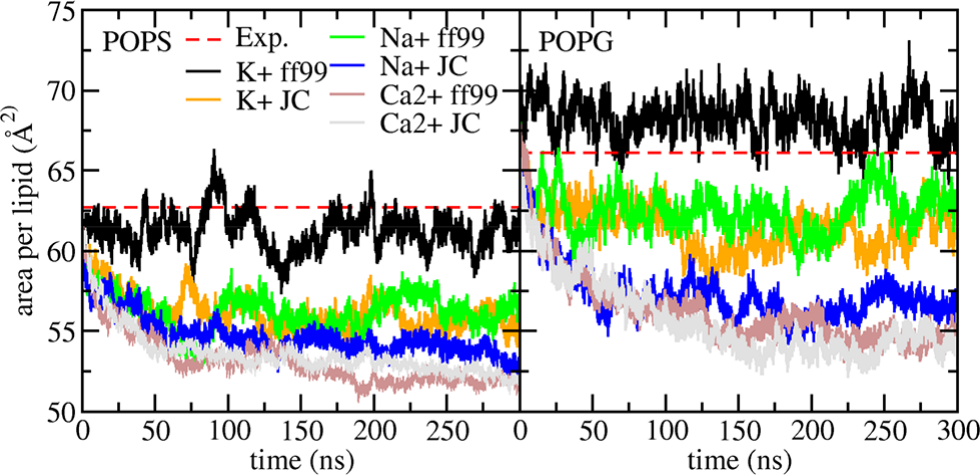
Lipid21 area per lipid results for POPS and POPG simulations using K+, Na+, or Ca2+ counterions modeled with ff99 or JC parameters. Only the K+ ff99 simulations maintain an area per lipid close to the experimental value.

Validation simulations for the anionic lipids PG, PS, and PH– used potassium counterions modeled with the Åqvist (ff99) force field^27^ to charge neutralize the simulation box, as required by PME^23^ to treat long-range electrostatics. The high concentration of cations in the water layer is likely physically unrealistic;^63^ further, the predominant extracellular cation is sodium, with potassium found with higher concentration inside the cell.^64^ The ff99 cation model was previously found to be defective, leading to formation of unphysical salt crystals when modeling high salt concentrations;^65^ Joung and Cheatham therefore considerably revised ion parameters for AMBER simulations.^65b^ However, during validation runs of anionic lipids using either K+, Na+, or Ca2+ counterions and JC parameters, bilayers were observed to condense and adopt an area per lipid below experiment (see Figure 9). In fact, even Na+ with ff99 resulted in similar behavior. We therefore used K+ counterions and ff99 parameters in the present work.

Unphysical lipid-ion pairing behavior is a known artifact in simulations of charged lipid bilayers and can result in condensing of the membrane and structural headgroup effects.^66^ Ion binding to lipid head groups has been an active area of research both computationally and experimentally, with the current view being that monovalent cations do not bind specifically to PC head groups at submolar concentrations (except Li+), whereas multivalent ions (such as Ca2+) do show specific binding.^67^ In the case of anionic head groups such as PS, monovalent ions show only weak binding, while Li+ and multivalent ions are able to form strong dehydrated complexes.^55^

Correct ion pairing behavior has been difficult to capture in molecular dynamics simulations, with a number of methods implemented to improve the description of lipid–ion interactions. Venable et al. derived pair-specific ion parameters for sodium,^66^ modifying the van der Waals interaction between Na+ and lipid headgroup oxygen atoms,^68^ allowing better agreement with NMR experiments for PG and PS simulations. Melcr et al. modified the Lipid14 POPC model to better capture cation interactions using the “electronic continuum correction” method, which involves scaling of the cation charge and lipid headgroup partial charges to account for electronic polarizability.^69^ This approach resulted in better agreement with NMR headgroup order parameter changes upon the addition of Na+ or Ca2+ ions (the “molecular electrometer” concept).

**Figure 10:**
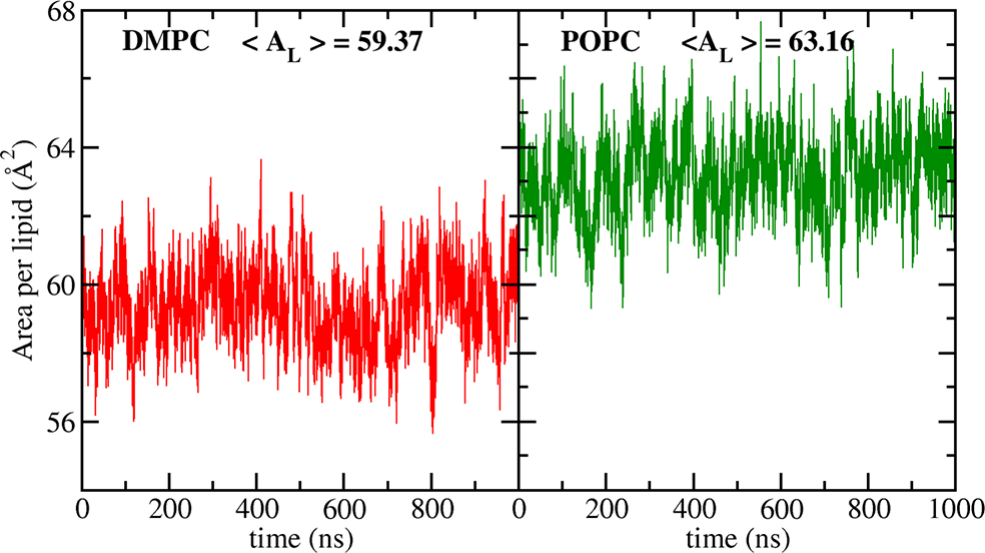
Lipid21 area per lipid results for 1 μs simulations of DMPC and POPC with 0.15 M NaCl modeled with JC ion parameters at 303 K. Bilayers maintain the correct phase with an area per lipid close to that of the validation simulations (see Table 1).

Such reparameterization is evidentially required for Lipid21 but is beyond the scope of the present work. Rather, we recommend K+ cations with ff99 parameters for simulations of entirely anionic lipid types. For typical simulations, bilayers are likely to be predominantly composed of PC lipids with lower fractions of anionic lipid types. Anticipating this, we ran μs simulations of POPC and DMPC with 0.15 M NaCl using JC parameters, as are typically paired with AMBER protein or DNA force fields. As demonstrated in Figure 10, this does not significantly alter the bilayer structure, unlike the effect observed for anionic lipids.

### Lipid Raft-like Simulations

The ability to model diverse lipid species with Lipid21 enables simulations of complex membranes of different lipid compositions. Cholesterol has a known aversion to poly unsaturated fatty acid lipids such as DAPC, adopting a larger tilt angle in DAPC bilayers than DPPC.^70^ Molecular dynamics (MD) studies have also found cholesterol to display a greater flip-flop rate in bilayers doped with PUFA lipids.^71^ In the extreme, cholesterol in pure DAPC bilayers shows a preference to reside at the membrane interior, as studied with neutron diffraction and MD.^72^

**Figure 11:**
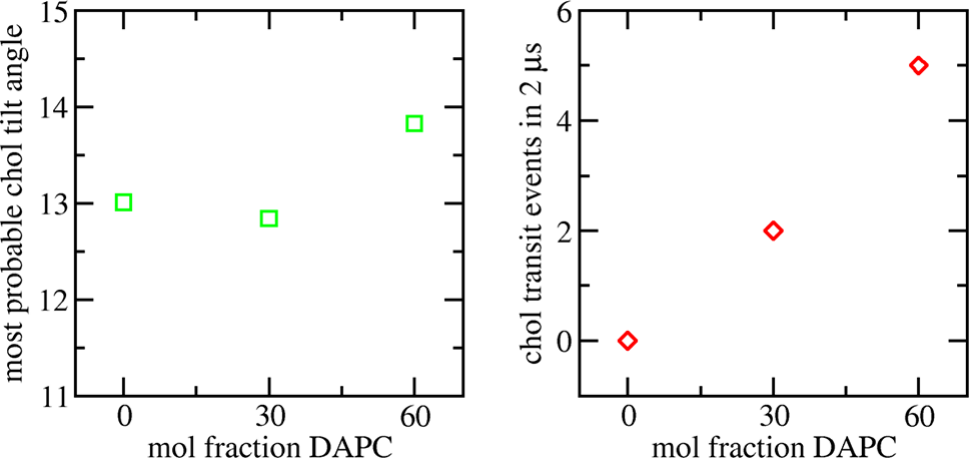
(Left) Most probable cholesterol tilt angle in raft-like membranes containing increasing mol % of DAPC. (Right) Total cholesterol transit events over a combined 2 μs of simulation as a function of increasing mol % of DAPC.

We constructed and simulated three raft-like membranes, containing POPC, PSM, cholesterol, and increasing fractions of the PUFA lipid DAPC (see Table 2) with Lipid21 to investigate cholesterol tilt angle and transit events as a function of DAPC doping. These underwent heating and equilibration of 100 ns at 310 K followed by two repeat production runs of 1 μs. Figure 11 plots the most probable cholesterol tilt angle, taken over all 40 cholesterol copies and two repeat 1 μs simulations, as a function of increasing DAPC doping. Also shown are the total number of cholesterol transit events over a combined 2 μs of simulation as a function of increasing DAPC, with a transit event being considered as any visit of cholesterol oxygen atoms to the bilayer center.

It can be observed from Figure 11 that the most probable cholesterol tilt angle in 0 mol % and 30 mol % DAPC raft-like bilayers is very similar, approximately 13°. At 60 mol % DAPC, this increases to approximately 14°. The raw probability distribution plots (SI Figure S9) reveal a broadening of the probability density, indicating destabilization of cholesterol molecules with increasing DAPC doping. Figure 11 also depicts the total number of cholesterol transit events as a function of DAPC doping. In 0 mol % DAPC bilayers, cholesterol molecules are very stable, displaying no transit events. At 30 mol % DAPC, there are two transit events. Analysis of the raw *z*-coordinate plots (SI Figure S10) show two complete flip-flop events, one from each 1 μs repeat simulation. Finally, at 60 mol % DAPC, cholesterol molecules are further destabilized, showing a total of five transit events, a mixture of complete flip-flop events and transient visits to the bilayer center. The increase in both tilt angle and transit events indicate that with increasing mol % of DAPC, Lipid21 qualitatively reproduces the observation of the perturbing effect of DAPC on cholesterol molecules.

## Discussion and Conclusions

The current work extends and improves the AMBER lipid force field, increasing coverage to anionic lipids, PUFA lipids, and sphingomyelin. The modular nature of the force field allows easy expansion to other lipid species. The PC model is improved; in particular, headgroup NMR order parameters are better reproduced in comparison to Lipid14. Bulk bilayer structural properties find good comparison to experiment for all lipids studied, matching well areas per lipid, bilayer thickness, NMR order parameters and X-ray scattering profiles. Simulations of raft-like membranes demonstrate an expected perturbation of cholesterol molecules as a function of increasing PUFA content.

The parametrization strategy required that the hydrocarbon parameters be updated, such that the DPPC melting point is better captured. Lipid headgroup partial charges are derived at a higher level of QM theory, including use of the polarizable continuum model allowing implicit inclusion of polarization. Lipid headgroup torsion parameters are updated to better reproduce QM energies.

Further work does remain concerning membrane simulations in AMBER. Although results for sphingomyelin reproduce available experimental data reasonably well, the area per lipid remains 4–5 Å too low. Head group NMR order parameters are improved for PC lipids; however, PG and PS do not find such a close match to experimental data. The type of cation used to charge neutralize anionic bilayer systems and the force field used to model ions is found to have a dramatic effect, artificially condensing POPS and POPG systems unless potassium counterions with Åqvist parameters are used. Correctly capturing the interaction of cations with different lipids species is an active area of research, with a number of methods proposed. Clearly, such work is required to update Lipid21. However, for simulations of physiologically relevant membranes, typically consisting of PC lipids and smaller fractions of other species, we recommend NaCl/KCl ions using JC parameters, as are typically combined with AMBER protein or DNA force fields. Indeed, 1 μs simulations of POPC and DMPC with 0.15 M NaCl using JC parameters reproduce experimental areas per lipid. Additionally, we identify that the Monte Carlo barostat results in the depression of areas per lipid for all lipid types studied in this work (see SI Table S1) and recommend the Berendsen barostat for pressure coupling for lipid bilayer simulations with AMBER.

Lipid21 significantly advances lipid membrane simulations with AMBER. The modular nature of the force field makes further lipid coverage achievable with minimal parametrization. We find suitable comparison to experiment for the lipids investigated. We also identify areas for improvement and recommend best practices for membrane simulations using Lipid21. The parameter set is available in the Supporting Information or can be downloaded from https://github.com/callumjd/lipid21.

## Supporting Information Available

Details of partial charge fitting, bilayer simulation results using the Monte Carlo barostat, torsion fitting profiles, details of cholesterol tilt angle, and transit events during raft-like simulations and the full Lipid21 parameter set (PDF)

## Abbreviations

MD: molecular dynamics
PC: phosphatidylcholine
PE: phosphatidylethanolamine
PS: phosphatidylserine
PG: phosphatidylglycerol
PUFA: polyunsaturated fatty acid
QM: quantum mechanics
