# Supplementary material for: Lipid21: Complex Lipid Membrane Simulations with AMBER
Source: J Chem Theory Comput. 2022 Feb 3;18(3):1726–36. doi: 10.1021/acs.jctc.1c01217 (PMC9007451; doi:10.1021/acs.jctc.1c01217)
Supplement: Supplementary file 1 — ct1c01217_si_001.pdf [file ct1c01217_si_001.pdf]

SUPPORTING INFORMATION

Lipid21: Complex Lipid Membrane Simulations with AMBER

Callum J. Dickson,[1] Ross C. Walker,[2,3] Ian R. Gould[4]*

[1]*Computer-Aided Drug Discovery, Global Discovery Chemistry, Novartis Institutes for BioMedical Research, 181 Massachusetts Avenue, Cambridge, Massachusetts 02139, United States*

[2]*GlaxoSmithKline PLC, 1250 S. Collegeville Rd, Collegeville, PA, 19426, United States*

[3]*Department of Chemistry and Biochemistry, University of California, San Diego, 9500 Gilman Drive, La Jolla, CA, 92093, United States*

[4]*Department of Chemistry, Imperial College London, London, SW7 2AZ, UK*

CORRESPONDING AUTHOR

*telephone: +44 (0)20 7594 5809

*email: i.gould@imperial.ac.uk

Lipid partial charge derivation

A capping procedure was used for the lipid head group and tail group partial charge fitting. See Lipid11 reference for full details.[1]

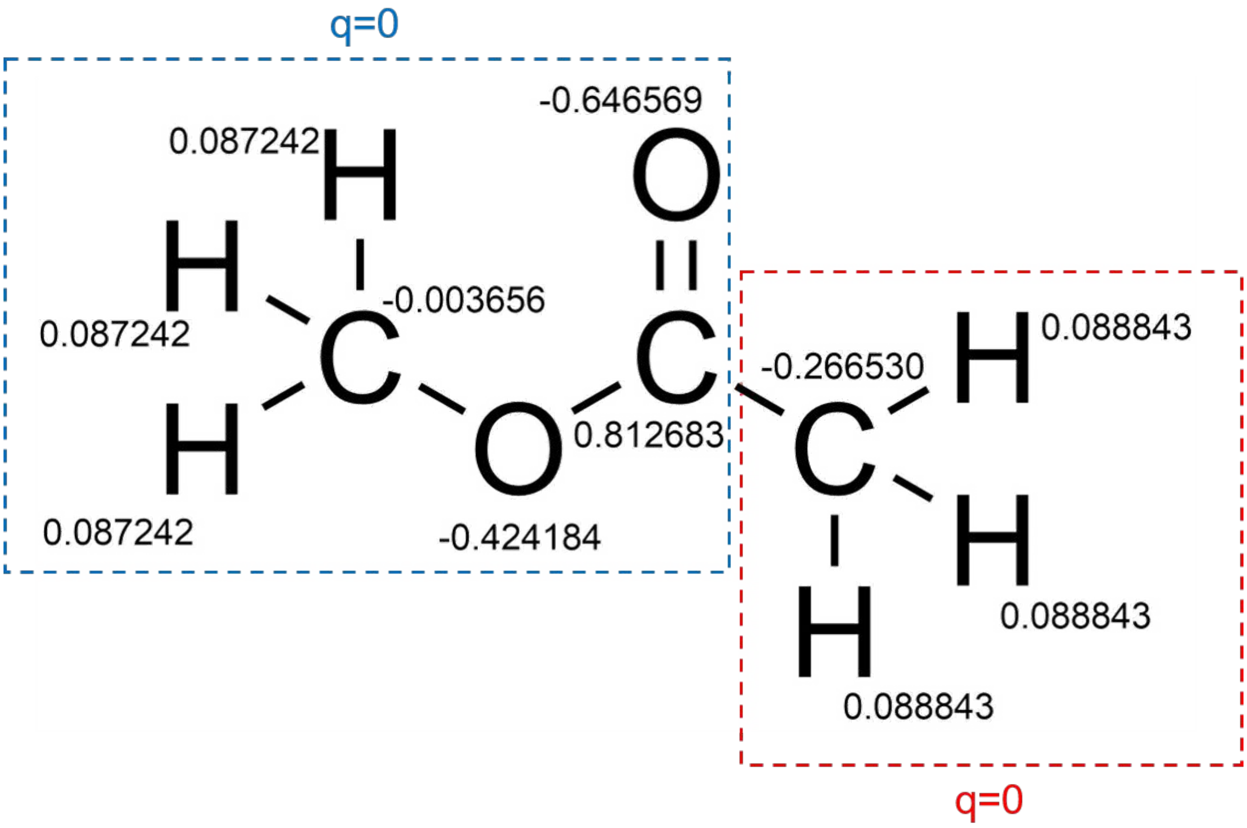

Figure S1 – Methyl acetate partial charges for head and tail group caps, derived at MP2/cc-pVTZ level with Polarizable Continuum Model.

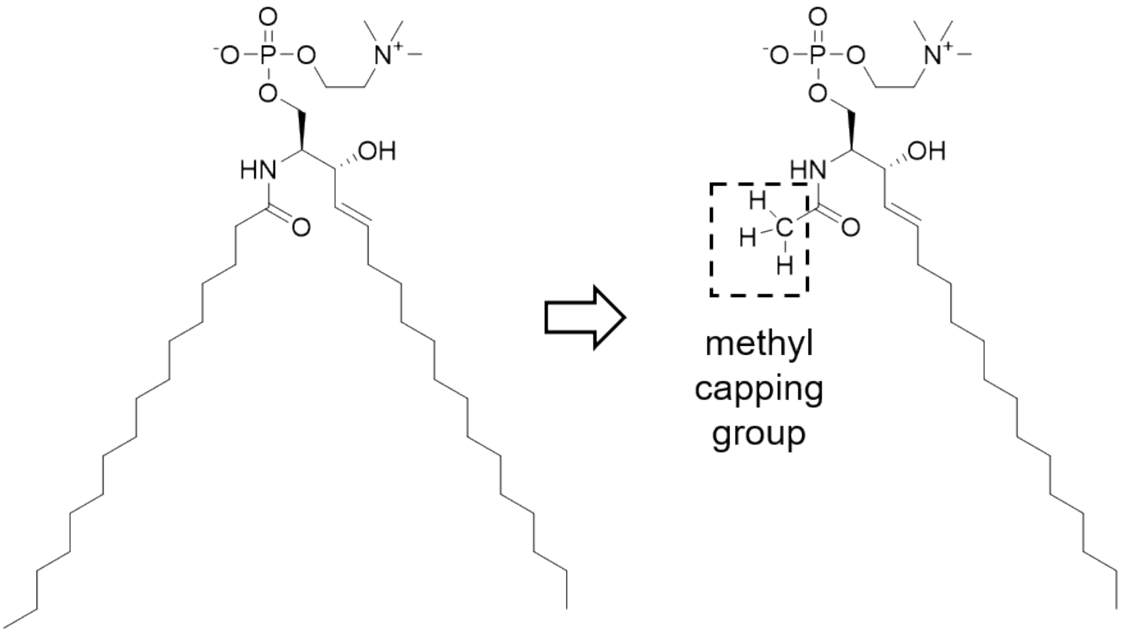

Figure S2 – Capping procedure used for fitting of sphingomyelin head group and sphingosine chain partial charges.

Table S1 – Area per lipid values for lipid bilayers simulated with Lipid21 and either the Berendsen or Monte Carlo barostats. Results for the Berendsen barostat are identical to those in Table 1 of the main text and are averages over 3x repeats of 300 ns. Results for the Monte Carlo are from a single run of 300 ns with identical starting coordinates. The average delta is 3.78 Å^2.

Lipid type	Area per lipid (Å^2) Berendsen barostat	Area per lipid (Å^2) Monte Carlo barostat	Delta (Å^2)
DLPC	61.14	57.92	3.22
DMPC	59.71	55.84	3.88
DPPC	61.69	57.65	4.04
DSPC	59.66	54.83	4.83
DOPC	66.95	62.68	4.27
POPC	63.92	59.93	3.99
POPE	55.92	52.49	3.42
DLPG	65.80	62.29	3.51
DMPG	65.25	61.45	3.80
DPPG	67.72	63.78	3.94
DSPG	66.65	61.51	5.14
DOPG	71.08	67.48	3.60
POPG	68.23	64.06	4.17
DOPS	65.47	61.75	3.73
POPS	61.78	58.90	2.88
POPA	63.90	60.35	3.55
DAPC	71.81	68.63	3.18
SDPC	65.10	61.24	3.87
PSM	58.50	55.59	2.91
SSM	57.10	53.48	3.61

Torsion parameter fitting

Hydrocarbon chain and choline torsions re-fitted to QM scans at MP2/cc-pVDZ level in this work. The majority of these torsions were also fitted to QM scans during the Lipid14 force-field fitting, so the level of

agreement for the Lipid14 model is not surprising. For the cB-cB-cD-cB torsion, these parameters are not present in the Lipid14 force field, so there is only comparison with QM.

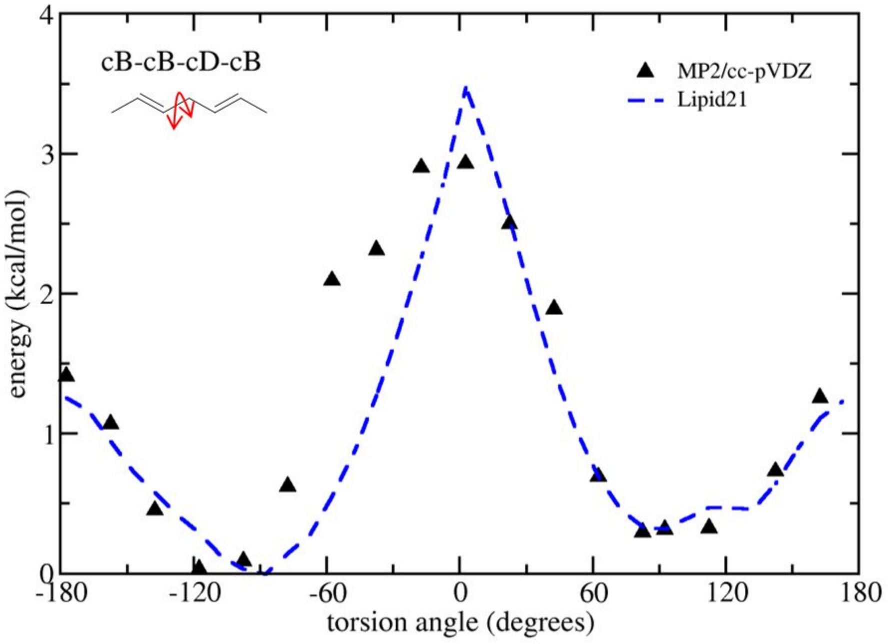

Figure S3 – Fitting of torsion cB-cB-cD-cB to QM scan.

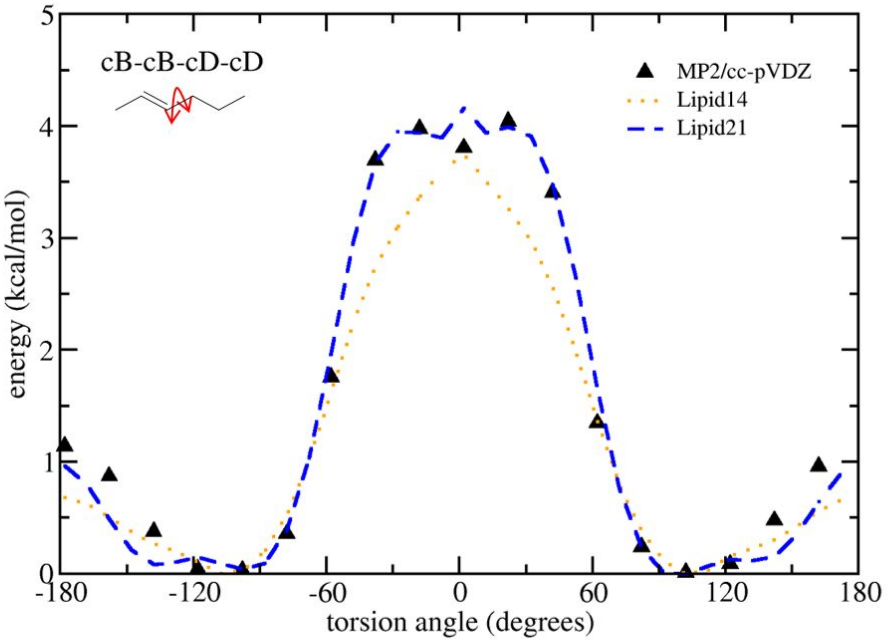

Figure S4 – Fitting of torsion cB-cB-cD-cD to QM scan.

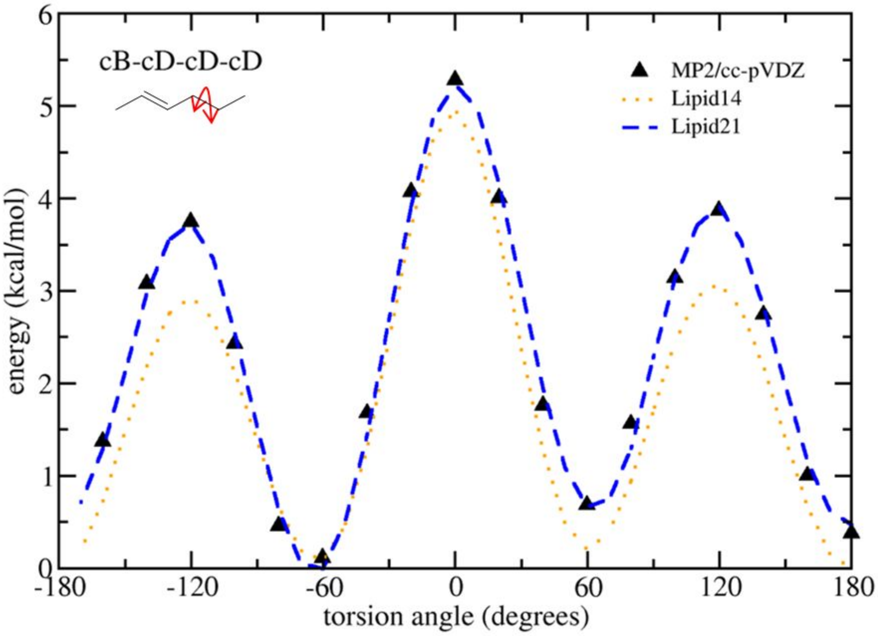

Figure S5 – Fitting of torsion cB-cD-cD-cD to QM scan.

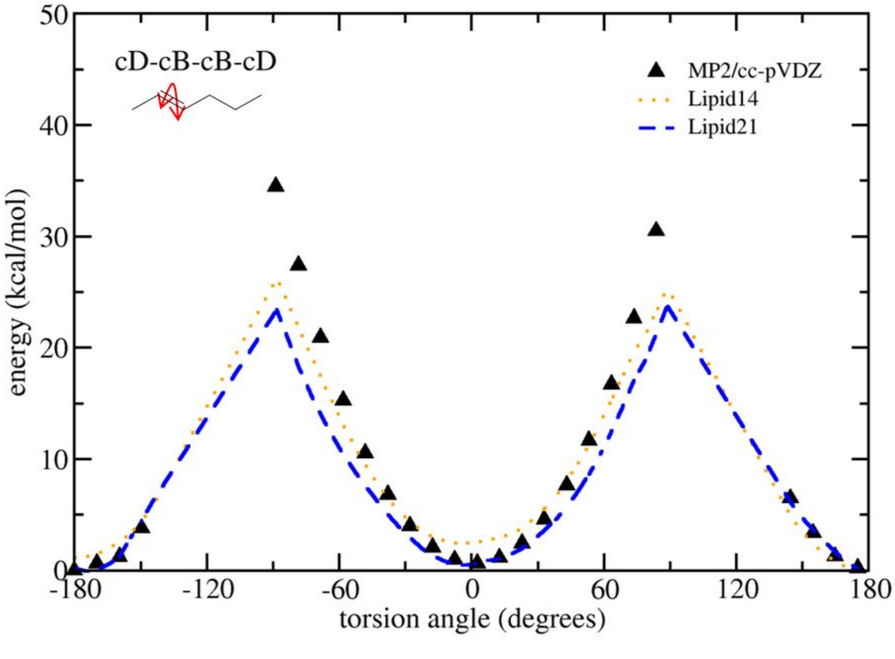

Figure S6 – Fitting of torsion cD-cB-cB-cD to QM scan.

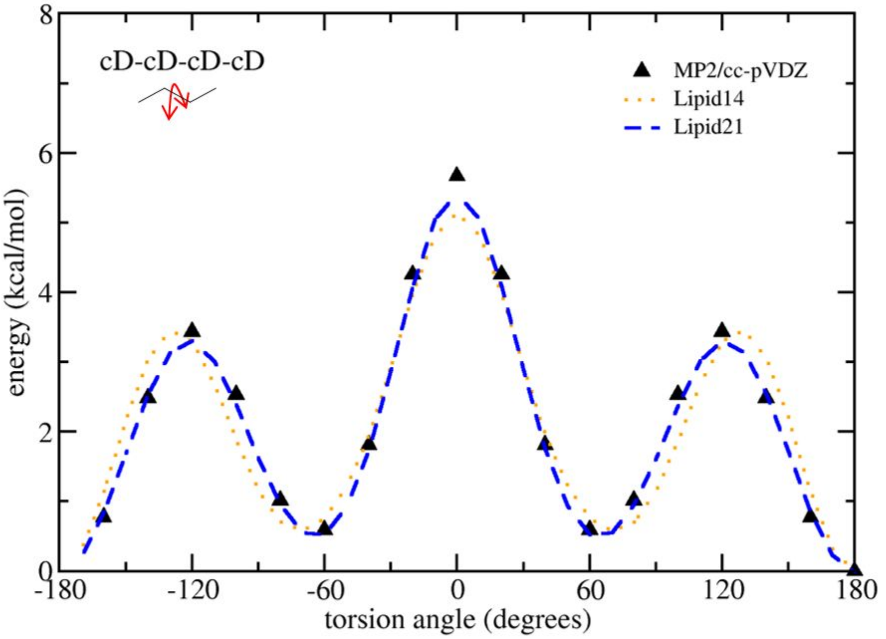

Figure S7 – Fitting of torsion cD-cD-cD-cD to QM scan.

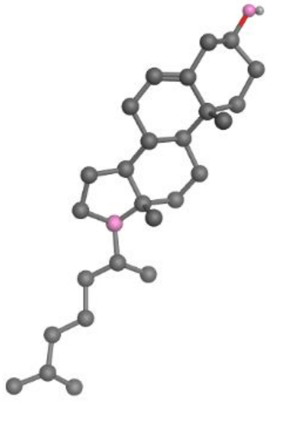

Figure S8 - Two cholesterol atoms used to monitor tilt angle during raft-like simulations.

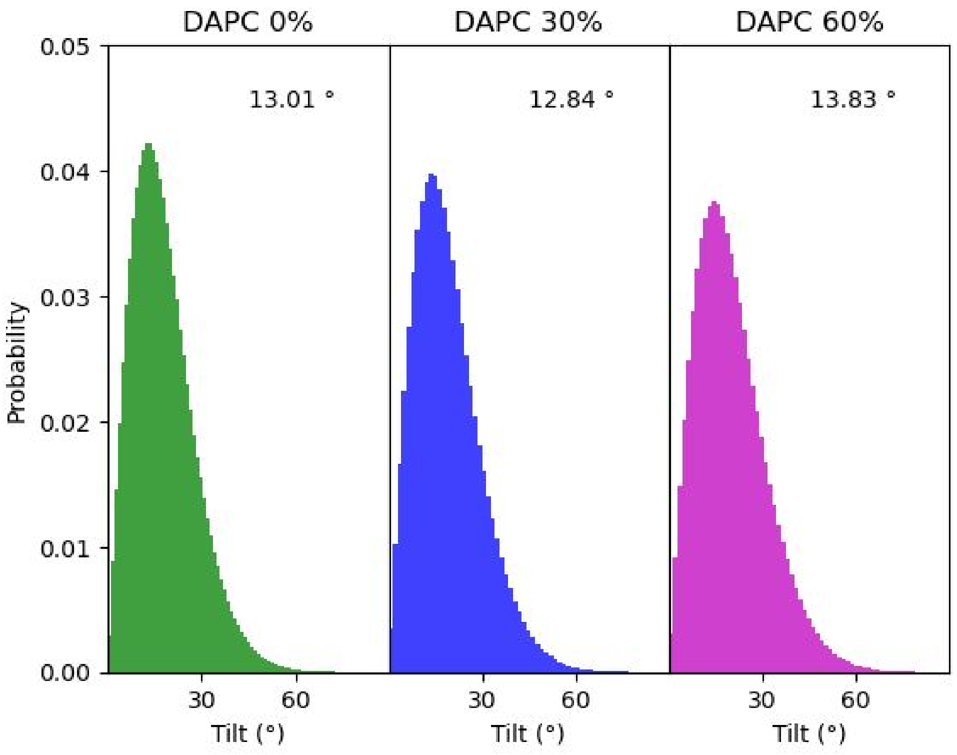

Figure S9 – Probability distribution of cholesterol tilt angle as a function of DAPC mol fraction doping.

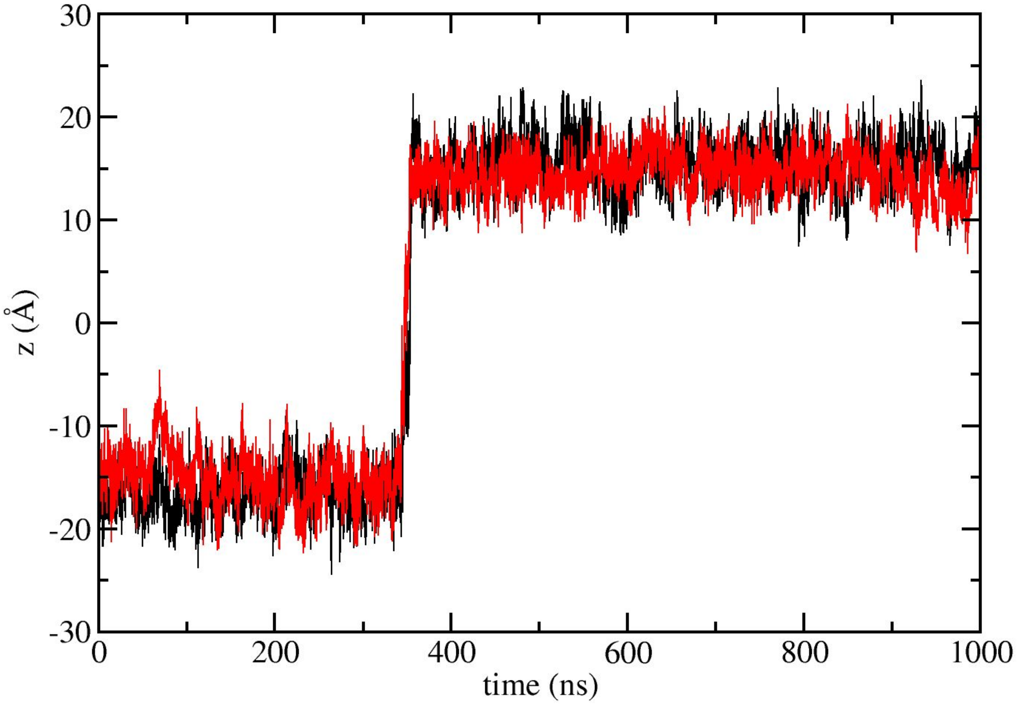

Figure S10 - Trace of z-coordinate of cholesterol oxygen atoms which underwent transit events during 30% mol fraction DAPC raft-like simulations. Transit events are from separate simulations.

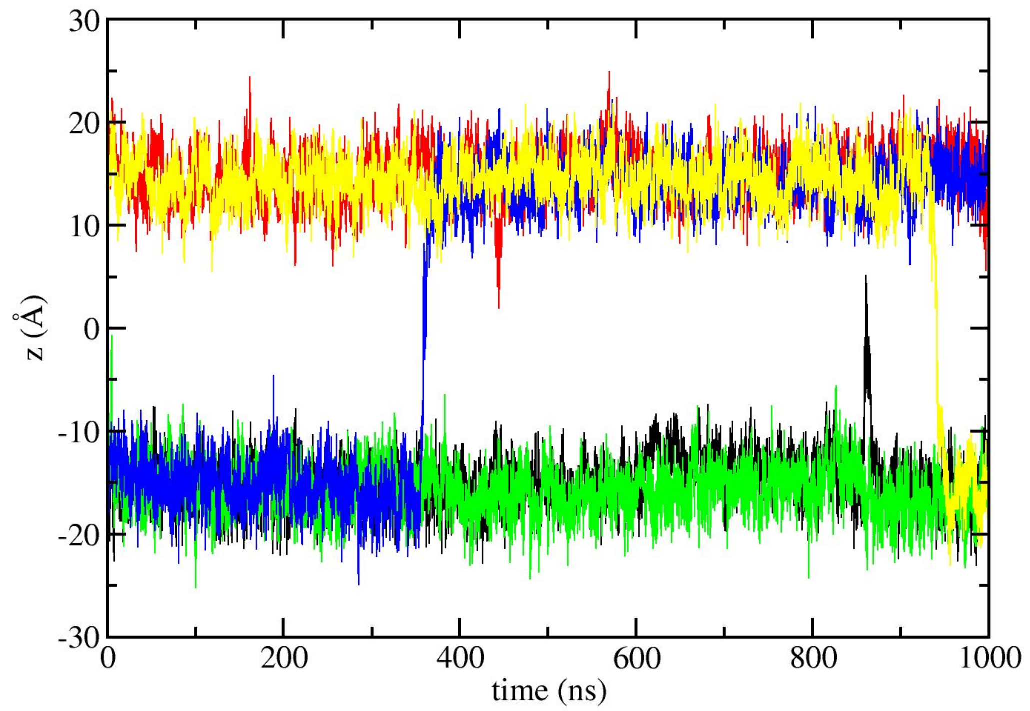

Figure S11 - Trace of z-coordinate of cholesterol oxygen atoms which underwent transit events during 60% mol fraction DAPC raft-like simulations.

Lipid21 parameters (lipid21.dat)

```
cA      12.010
cB      12.010
cC      12.010
cD      12.010
hA       1.008
hB       1.008
hE       1.008
hL       1.008
hN       1.008
hO       1.008
hX       1.008
nA      14.010
nN      14.010
oC      16.000
oH      16.000
oO      16.000
oP      16.000
oS      16.000
oT      16.000
pA      30.970
```

```
cD-cC    328.300   1.508
cC-cD    328.300   1.508
cD-cD    303.100   1.535
cA-cD    303.100   1.535
cC-oC    648.000   1.214
oS-cC    411.300   1.343
nA-cA    293.600   1.499
cA-cA    303.100   1.535
pA-oT    230.000   1.610  parm99
pA-oP    525.000   1.480  parm99
cA-oT    301.500   1.439
cA-oS    301.500   1.439
cA-hA    337.300   1.092
cD-hL    337.300   1.092
cA-hX    338.700   1.091
cA-hE    335.900   1.093
cB-cB    589.700   1.324
cB-cD    328.300   1.508
cB-cA    328.300   1.508
```

```
cB-hB    344.300   1.087
cA-cC    328.300   1.508
cC-oO    648.000   1.214
hN-nA    369.000   1.033
cA-oH    314.100   1.426
hO-oH    369.600   0.974
oH-pA    321.200   1.625
cC-nN    478.200   1.345
cA-nN    330.600   1.460
hN-nN    410.200   1.009


cD-cD-cD     47.200  112.050  Lipid21 v1.0 MP2 QM fit.
cD-cC-oC     68.030  123.110
cD-cC-oS     69.260  111.960
cD-cD-cC     63.790  110.530
hA-cA-hA     39.430  108.350
cA-cA-hA     46.370  110.050
cD-cA-hA     46.370  110.050
cA-cD-hL     46.370  110.050
cA-cA-cA     63.210  110.630
cD-cD-cA     63.210  110.630
cA-cA-cD     63.210  110.630
cA-cA-cB     63.530  111.440
cA-cB-cB     64.330  123.420
cA-cB-cA     62.700  116.520
cA-cB-hB     45.660  117.300
cB-cA-hA     47.030  110.490
oS-cC-oC     75.930  123.330
oP-pA-oP    140.000  119.900  from O2-P -O2
cA-nA-cA     62.840  110.640
cA-cA-nA     64.450  114.320
oT-pA-oP    100.000  108.230  from O2-P -OS
oT-cA-cA     67.780  108.420
pA-oT-cA    100.000  120.500  from CT-OS-P
oT-pA-oT     90.000  102.600  from OS-P -OS
cA-cA-oS     67.780  108.420
cA-oS-cC     63.630  115.140
hL-cD-cC     47.200  109.680
hL-cD-hL     39.430  108.350
hL-cD-cD     46.370  110.050
hX-cA-hX     39.040  110.740
nA-cA-hX     49.020  107.910
```

hE-cA-cA	46.360	110.070
hE-cA-hE	39.180	109.550
cA-cA-hX	46.020	111.740
oT-cA-hE	50.840	108.820
hE-cA-oS	50.840	108.820
cB-cB-cD	64.330	123.420
cB-cD-hL	47.030	110.490
cB-cD-cD	63.530	111.440
cB-cB-hB	50.040	120.940
cD-cB-hB	45.660	117.300
cA-cC-oO	68.030	123.110
oO-cC-oO	78.170	130.380
cA-nA-hN	46.190	110.110
hN-nA-hN	40.520	108.110
cA-cA-cC	63.790	110.530
cC-cA-nA	65.070	114.210
cC-cA-hX	47.230	109.540
hE-cA-oH	50.970	109.880
cA-oH-hO	47.090	108.160
cA-cA-oH	67.720	109.430
hO-oH-pA	55.880	110.140
oH-pA-oT	44.910	102.370
oP-pA-oH	43.790	115.260
cB-cD-cB	63.930	112.080
cA-cA-nN	65.850	112.130
cA-nN-cC	63.920	121.350
cA-nN-hN	46.040	116.780
cC-nN-hN	49.210	118.460
cD-cC-nN	67.860	115.150
hE-cA-nN	49.820	109.320
nN-cC-oC	75.830	122.030
cB-cA-oH	68.180	110.210
cB-cA-hE	47.030	110.460

cD-cD-cD-cD	1	0.100	0.000	-5.0	SCEE=1.2	SCNB=6.0	Lipid21 v1.0 MP2 QM fit.
cD-cD-cD-cD	1	0.100	0.000	-4.0	SCEE=1.2	SCNB=6.0	Lipid21 v1.0 MP2 QM fit.
cD-cD-cD-cD	1	0.300	0.000	-3.0	SCEE=1.2	SCNB=6.0	Lipid21 v1.0 MP2 QM fit.
cD-cD-cD-cD	1	0.100	0.000	-2.0	SCEE=1.2	SCNB=6.0	Lipid21 v1.0 MP2 QM fit.
cD-cD-cD-cD	1	0.100	0.000	1.0	SCEE=1.2	SCNB=6.0	Lipid21 v1.0 MP2 QM fit.
cA-oT-pA-oP	1	0.000	0.000	-5.0	SCEE=1.2	SCNB=2.0	Lipid21 v1.0
cA-oT-pA-oP	1	0.000	0.000	-4.0	SCEE=1.2	SCNB=2.0	Lipid21 v1.0
cA-oT-pA-oP	1	0.000	0.000	-3.0	SCEE=1.2	SCNB=2.0	Lipid21 v1.0

```
cA-oT-pA-oP    1    0.200    0.000  -2.0    SCEE=1.2 SCNB=2.0  Lipid21 v1.0
cA-oT-pA-oP    1    0.000    0.000   1.0    SCEE=1.2 SCNB=2.0  Lipid21 v1.0
hE-cA-oT-pA    1    0.000    0.000  -5.0    SCEE=1.2 SCNB=2.0  Lipid21 v1.0
hE-cA-oT-pA    1    0.000    0.000  -4.0    SCEE=1.2 SCNB=2.0  Lipid21 v1.0
hE-cA-oT-pA    1    0.383    0.000  -3.0    SCEE=1.2 SCNB=2.0  Lipid21 v1.0
hE-cA-oT-pA    1    0.000    0.000  -2.0    SCEE=1.2 SCNB=2.0  Lipid21 v1.0
hE-cA-oT-pA    1    0.000    0.000   1.0    SCEE=1.2 SCNB=2.0  Lipid21 v1.0
oS-cA-cA-cA    1    0.000    0.000  -5.0    SCEE=1.2 SCNB=2.0  Lipid21 v1.0
oS-cA-cA-cA    1    0.000    0.000  -4.0    SCEE=1.2 SCNB=2.0  Lipid21 v1.0
oS-cA-cA-cA    1    0.000    0.000  -3.0    SCEE=1.2 SCNB=2.0  Lipid21 v1.0
oS-cA-cA-cA    1    0.000    0.000  -2.0    SCEE=1.2 SCNB=2.0  Lipid21 v1.0
oS-cA-cA-cA    1    0.000    0.000   1.0    SCEE=1.2 SCNB=2.0  Lipid21 v1.0
oT-cA-cA-oS    1    0.000    0.000  -5.0    SCEE=1.2 SCNB=2.0  Lipid21 v1.0
oT-cA-cA-oS    1    0.000    0.000  -4.0    SCEE=1.2 SCNB=2.0  Lipid21 v1.0
oT-cA-cA-oS    1    0.000    0.000  -3.0    SCEE=1.2 SCNB=2.0  Lipid21 v1.0
oT-cA-cA-oS    1    0.000    0.000  -2.0    SCEE=1.2 SCNB=2.0  Lipid21 v1.0
oT-cA-cA-oS    1    0.000    0.000   1.0    SCEE=1.2 SCNB=2.0  Lipid21 v1.0
cC-oS-cA-cA    1   -0.200  300.000  -3.0    SCEE=1.2 SCNB=2.0  Lipid21 v1.0 MP2 QM fit.
cC-oS-cA-cA    1   -0.300  240.000  -2.0    SCEE=1.2 SCNB=2.0  Lipid21 v1.0 MP2 QM fit.
cC-oS-cA-cA    1   -0.100    0.000   1.0    SCEE=1.2 SCNB=2.0  Lipid21 v1.0 MP2 QM fit.
oS-cA-cA-oS    1    1.000    0.000  -3.0    SCEE=1.2 SCNB=2.0  Lipid21 v1.0 MP2 QM fit.
oS-cA-cA-oS    1    0.300    0.000  -2.0    SCEE=1.2 SCNB=2.0  Lipid21 v1.0 MP2 QM fit.
oS-cA-cA-oS    1   -0.100  180.000   1.0    SCEE=1.2 SCNB=2.0  Lipid21 v1.0 MP2 QM fit.
cA-oT-pA-oT    1    0.600    0.000  -3.0    SCEE=1.2 SCNB=2.0  Lipid21 v1.0 MP2 PCM QM fit.
cA-oT-pA-oT    1    2.300    0.000   2.0    SCEE=1.2 SCNB=2.0  Lipid21 v1.0 MP2 PCM QM fit.
cA-cA-oT-pA    1    0.000    0.000  -3.0    SCEE=1.2 SCNB=2.0  Lipid21 v1.0 MP2 PCM QM fit.
cA-cA-oT-pA    1   -0.800  180.000   2.0    SCEE=1.2 SCNB=2.0  Lipid21 v1.0 MP2 PCM QM fit.
cA-cA-cA-oT    1    0.400   60.000  -3.0    SCEE=1.2 SCNB=2.0  Lipid21 v1.0 MP2 PCM QM fit.
cA-cA-cA-oT    1    0.200  120.000  -2.0    SCEE=1.2 SCNB=2.0  Lipid21 v1.0 MP2 PCM QM fit.
cA-cA-cA-oT    1    0.800  180.000   1.0    SCEE=1.2 SCNB=2.0  Lipid21 v1.0 MP2 PCM QM fit.
nA-cA-cA-oT    1    1.000    0.000   3.0    SCEE=1.2 SCNB=2.0  Lipid21 v1.0 MP2 PCM QM fit.
cD-cB-cB-cD    1    0.000    0.000  -5.0    SCEE=1.2 SCNB=2.0  Lipid21 v1.0 MP2 QM fit.
cD-cB-cB-cD    1    0.000    0.000  -4.0    SCEE=1.2 SCNB=2.0  Lipid21 v1.0 MP2 QM fit.
cD-cB-cB-cD    1    0.600    0.000  -3.0    SCEE=1.2 SCNB=2.0  Lipid21 v1.0 MP2 QM fit.
cD-cB-cB-cD    1    3.300  180.000  -2.0    SCEE=1.2 SCNB=2.0  Lipid21 v1.0 MP2 QM fit.
cD-cB-cB-cD    1    1.700  180.000   1.0    SCEE=1.2 SCNB=2.0  Lipid21 v1.0 MP2 QM fit.
cA-cB-cB-cA    1    6.650  180.000  -2.0    SCEE=1.2 SCNB=2.0
cA-cB-cB-cA    1    1.900  180.000   1.0    SCEE=1.2 SCNB=2.0
cD-cB-cB-cA    1    6.650  180.000  -2.0    SCEE=1.2 SCNB=2.0
cD-cB-cB-cA    1    1.900  180.000   1.0    SCEE=1.2 SCNB=2.0
cB-cB-cD-cD    1    0.200  180.000  -5.0    SCEE=1.2 SCNB=2.0  Lipid21 v1.0 MP2 QM fit.
cB-cB-cD-cD    1    0.200  180.000  -4.0    SCEE=1.2 SCNB=2.0  Lipid21 v1.0 MP2 QM fit.
cB-cB-cD-cD    1    0.400  180.000  -3.0    SCEE=1.2 SCNB=2.0  Lipid21 v1.0 MP2 QM fit.
```

```
cB-cB-cD-cD    1    0.400  180.000  -2.0    SCEE=1.2 SCNB=2.0  Lipid21 v1.0 MP2 QM fit.
cB-cB-cD-cD    1    0.600    0.000   1.0    SCEE=1.2 SCNB=2.0  Lipid21 v1.0 MP2 QM fit.
cA-cA-cB-cB    1    0.000    0.000   2.0    SCEE=1.2 SCNB=2.0
cB-cD-cD-cD    1    0.100    0.000  -5.0    SCEE=1.2 SCNB=2.0  Lipid21 v1.0 MP2 QM fit.
cB-cD-cD-cD    1    0.000    0.000  -4.0    SCEE=1.2 SCNB=2.0  Lipid21 v1.0 MP2 QM fit.
cB-cD-cD-cD    1    0.500    0.000  -3.0    SCEE=1.2 SCNB=2.0  Lipid21 v1.0 MP2 QM fit.
cB-cD-cD-cD    1    0.000    0.000  -2.0    SCEE=1.2 SCNB=2.0  Lipid21 v1.0 MP2 QM fit.
cB-cD-cD-cD    1    0.000    0.000   1.0    SCEE=1.2 SCNB=2.0  Lipid21 v1.0 MP2 QM fit.
cB-cA-cA-cA    1    0.000    0.000  -5.0    SCEE=1.2 SCNB=2.0  Lipid21 v1.0
cB-cA-cA-cA    1    0.000    0.000  -4.0    SCEE=1.2 SCNB=2.0  Lipid21 v1.0
cB-cA-cA-cA    1    0.000    0.000  -3.0    SCEE=1.2 SCNB=2.0  Lipid21 v1.0
cB-cA-cA-cA    1    0.000    0.000  -2.0    SCEE=1.2 SCNB=2.0  Lipid21 v1.0
cB-cA-cA-cA    1    0.000    0.000   1.0    SCEE=1.2 SCNB=2.0  Lipid21 v1.0
cD-cD-cC-oC    1    0.000  180.000   2.0    SCEE=1.2 SCNB=2.0
cD-cD-cC-oS    1    0.000  180.000   2.0    SCEE=1.2 SCNB=2.0
cD-cC-oS-cA    1    2.700  180.000   2.0    SCEE=1.2 SCNB=2.0
cC-cD-cD-cD    1    0.156    0.000   3.0    SCEE=1.2 SCNB=2.0
cA-cA-nA-cA    1    0.156    0.000   3.0    SCEE=1.2 SCNB=2.0
cA-oS-cC-oC    1    2.700  180.000  -2.0    SCEE=1.2 SCNB=2.0
cA-oS-cC-oC    1    1.400  180.000   1.0    SCEE=1.2 SCNB=2.0
hL-cD-cC-oC    1    0.080  180.000  -3.0    SCEE=1.2 SCNB=2.0
hL-cD-cC-oC    1    0.800    0.000   1.0    SCEE=1.2 SCNB=2.0
hL-cD-cC-oS    1    0.000  180.000   2.0    SCEE=1.2 SCNB=2.0
hL-cD-cD-hL    1    0.150    0.000   3.0    SCEE=1.2 SCNB=2.0
hL-cD-cD-cC    1    0.156    0.000   3.0    SCEE=1.2 SCNB=2.0
hL-cD-cD-cD    1    0.160    0.000   3.0    SCEE=1.2 SCNB=2.0
cD-cD-cD-hL    1    0.160    0.000   3.0    SCEE=1.2 SCNB=2.0
cC-cD-cD-hL    1    0.156    0.000   3.0    SCEE=1.2 SCNB=2.0
hX-cA-nA-cA    1    0.156    0.000   3.0    SCEE=1.2 SCNB=2.0
hE-cA-cA-hX    1    0.156    0.000   3.0    SCEE=1.2 SCNB=2.0
hE-cA-cA-nA    1    0.156    0.000   3.0    SCEE=1.2 SCNB=2.0
oT-cA-cA-hX    1    0.156    0.000   3.0    SCEE=1.2 SCNB=2.0
hE-cA-cA-oS    1    0.000    0.000  -3.0    SCEE=1.2 SCNB=2.0
hE-cA-cA-oS    1    0.250    0.000   1.0    SCEE=1.2 SCNB=2.0
hE-cA-cA-hE    1    0.156    0.000   3.0    SCEE=1.2 SCNB=2.0
hE-cA-cA-oT    1    0.000    0.000  -3.0    SCEE=1.2 SCNB=2.0
hE-cA-cA-oT    1    0.250    0.000   1.0    SCEE=1.2 SCNB=2.0
hE-cA-oS-cC    1    0.383    0.000   3.0    SCEE=1.2 SCNB=2.0
hE-cA-cA-cA    1    0.156    0.000   3.0    SCEE=1.2 SCNB=2.0
cA-cA-cA-hE    1    0.156    0.000   3.0    SCEE=1.2 SCNB=2.0
oS-cA-cA-hE    1    0.000    0.000  -3.0    SCEE=1.2 SCNB=2.0
oS-cA-cA-hE    1    0.250    0.000   1.0    SCEE=1.2 SCNB=2.0
cD-cD-cB-hB    1    0.000    0.000   2.0    SCEE=1.2 SCNB=2.0
```

```
hB-cB-cD-hL    1    0.000    0.000   2.0    SCEE=1.2 SCNB=2.0
cB-cD-cD-hL    1    0.1556   0.000   3.0    SCEE=1.2 SCNB=2.0
cD-cB-cB-hB    1    6.650  180.000   2.0    SCEE=1.2 SCNB=2.0
cB-cB-cD-hL    1    0.380  180.000  -3.0    SCEE=1.2 SCNB=2.0
cB-cB-cD-hL    1    1.150    0.000   1.0    SCEE=1.2 SCNB=2.0
hB-cB-cB-hB    1    6.650  180.000   2.0    SCEE=1.2 SCNB=2.0
cC-cA-nA-hN    1    0.1556   0.000   3.0    SCEE=1.2 SCNB=2.0
nA-cA-cC-oO    1    0.000  180.000   2.0    SCEE=1.2 SCNB=2.0
hN-nA-cA-hX    1    0.1556   0.000   3.0    SCEE=1.2 SCNB=2.0
hX-cA-cC-oO    1    0.000  180.000   2.0    SCEE=1.2 SCNB=2.0
cC-cA-cA-hE    1    0.1556   0.000   3.0    SCEE=1.2 SCNB=2.0
cA-cA-nA-hN    1    0.1556   0.000   3.0    SCEE=1.2 SCNB=2.0
cA-cA-cC-oO    1    0.000  180.000   2.0    SCEE=1.2 SCNB=2.0
cC-cA-cA-oT    1    0.1556   0.000   3.0    SCEE=1.2 SCNB=2.0
hE-cA-oH-hO    1    0.500    0.000   3.0    SCEE=1.2 SCNB=2.0
cA-cA-oH-hO    1    0.160    0.000  -3.0    SCEE=1.2 SCNB=2.0
cA-cA-oH-hO    1    0.250    0.000   1.0    SCEE=1.2 SCNB=2.0
hE-cA-cA-oH    1    0.000    0.000  -3.0    SCEE=1.2 SCNB=2.0
hE-cA-cA-oH    1    0.250    0.000   1.0    SCEE=1.2 SCNB=2.0
cA-cA-cA-oH    1    0.1556   0.000   3.0    SCEE=1.2 SCNB=2.0
oH-cA-cA-oT    1    0.144    0.000  -3.0    SCEE=1.2 SCNB=2.0
oH-cA-cA-oT    1    1.175    0.000   2.0    SCEE=1.2 SCNB=2.0
oH-cA-cA-oH    1    0.144    0.000  -3.0    SCEE=1.2 SCNB=2.0
oH-cA-cA-oH    1    1.175    0.000   2.0    SCEE=1.2 SCNB=2.0
cA-oT-pA-oH    1    0.250    0.000  -3.0    SCEE=1.2 SCNB=2.0
cA-oT-pA-oH    1    1.200    0.000   2.0    SCEE=1.2 SCNB=2.0
hO-oH-pA-oT    1    0.5333   0.000   3.0    SCEE=1.2 SCNB=2.0
hO-oH-pA-oP    1    0.5333   0.000   3.0    SCEE=1.2 SCNB=2.0
cB-cB-cD-cB    1    0.000    0.000  -5.0    SCEE=1.2 SCNB=2.0  Lipid21 v1.0 MP2 QM fit.
cB-cB-cD-cB    1    0.000    0.000  -4.0    SCEE=1.2 SCNB=2.0  Lipid21 v1.0 MP2 QM fit.
cB-cB-cD-cB    1    0.000    0.000  -3.0    SCEE=1.2 SCNB=2.0  Lipid21 v1.0 MP2 QM fit.
cB-cB-cD-cB    1    0.000    0.000  -2.0    SCEE=1.2 SCNB=2.0  Lipid21 v1.0 MP2 QM fit.
cB-cB-cD-cB    1    0.000    0.000   1.0    SCEE=1.2 SCNB=2.0  Lipid21 v1.0 MP2 QM fit.
cB-cD-cB-hB    1    0.000    0.000   2.0    SCEE=1.2 SCNB=2.0
cA-cA-cA-cA    1    0.180    0.000  -3.0    SCEE=1.2 SCNB=2.0
cA-cA-cA-cA    1    0.250  180.000  -2.0    SCEE=1.2 SCNB=2.0
cA-cA-cA-cA    1    0.200  180.000   1.0    SCEE=1.2 SCNB=2.0
cA-cA-cA-cD    1    0.180    0.000  -3.0    SCEE=1.2 SCNB=2.0
cA-cA-cA-cD    1    0.250  180.000  -2.0    SCEE=1.2 SCNB=2.0
cA-cA-cA-cD    1    0.200  180.000   1.0    SCEE=1.2 SCNB=2.0
cA-cA-cD-cD    1    0.180    0.000  -3.0    SCEE=1.2 SCNB=2.0
cA-cA-cD-cD    1    0.250  180.000  -2.0    SCEE=1.2 SCNB=2.0
cA-cA-cD-cD    1    0.200  180.000   1.0    SCEE=1.2 SCNB=2.0
```

```
cA-cD-cD-cD    1    0.180    0.000  -3.0    SCEE=1.2 SCNB=2.0
cA-cD-cD-cD    1    0.250  180.000  -2.0    SCEE=1.2 SCNB=2.0
cA-cD-cD-cD    1    0.200  180.000   1.0    SCEE=1.2 SCNB=2.0
cA-cA-cA-hA    1    0.160    0.000   3.0    SCEE=1.2 SCNB=2.0
cD-cA-cA-hA    1    0.160    0.000   3.0    SCEE=1.2 SCNB=2.0
cA-cA-cD-hL    1    0.160    0.000   3.0    SCEE=1.2 SCNB=2.0
hA-cA-cA-hA    1    0.150    0.000   3.0    SCEE=1.2 SCNB=2.0
hA-cA-cD-hL    1    0.150    0.000   3.0    SCEE=1.2 SCNB=2.0
cD-cD-cA-hA    1    0.160    0.000   3.0    SCEE=1.2 SCNB=2.0
cA-cD-cD-hL    1    0.160    0.000   3.0    SCEE=1.2 SCNB=2.0
cA-cA-cB-cA    1    0.000    0.000   2.0    SCEE=1.2 SCNB=2.0
cA-cB-cB-hB    1    6.650  180.000   2.0    SCEE=1.2 SCNB=2.0
hA-cA-cB-hB    1    0.000    0.000   2.0    SCEE=1.2 SCNB=2.0
cA-cA-cB-hB    1    0.000    0.000   2.0    SCEE=1.2 SCNB=2.0
cB-cA-cA-hA    1    0.1556   0.000   3.0    SCEE=1.2 SCNB=2.0
cB-cA-cA-oH    1    0.1556   0.000   3.0    SCEE=1.2 SCNB=2.0
cB-cB-cA-hA    1    0.380  180.000  -3.0    SCEE=1.2 SCNB=2.0
cB-cB-cA-hA    1    1.150    0.000   1.0    SCEE=1.2 SCNB=2.0
hA-cA-cA-oH    1    0.000    0.000  -3.0    SCEE=1.2 SCNB=2.0
hA-cA-cA-oH    1    0.250    0.000   1.0    SCEE=1.2 SCNB=2.0
cA-cB-cA-hA    1    0.000    0.000   2.0    SCEE=1.2 SCNB=2.0
hA-cA-cA-hE    1    0.1556   0.000   3.0    SCEE=1.2 SCNB=2.0
cB-cA-cA-hE    1    0.1556   0.000   3.0    SCEE=1.2 SCNB=2.0
cA-cA-nN-cC    1    0.000    0.000   2.0    SCEE=1.2 SCNB=2.0
cA-cA-nN-hN    1    0.000    0.000   2.0    SCEE=1.2 SCNB=2.0
cA-nN-cC-cD    1    2.500  180.000   2.0    SCEE=1.2 SCNB=2.0
cA-nN-cC-oC    1    2.500  180.000   2.0    SCEE=1.2 SCNB=2.0
cB-cA-cA-nN    1    2.400    0.000  -3.0    SCEE=1.2 SCNB=2.0  Lipid21 v1.0 MP2 QM fit.
cB-cA-cA-nN    1    1.300  180.000  -2.0    SCEE=1.2 SCNB=2.0  Lipid21 v1.0 MP2 QM fit.
cB-cA-cA-nN    1    0.400   60.000   1.0    SCEE=1.2 SCNB=2.0  Lipid21 v1.0 MP2 QM fit.
cC-nN-cA-hE    1    0.000    0.000   2.0    SCEE=1.2 SCNB=2.0
cD-cC-nN-hN    1    2.500  180.000   2.0    SCEE=1.2 SCNB=2.0
cD-cD-cC-nN    1    0.000  180.000   2.0    SCEE=1.2 SCNB=2.0
hE-cA-cA-nN    1    0.156    0.000   3.0    SCEE=1.2 SCNB=2.0
hE-cA-nN-hN    1    0.000    0.000   2.0    SCEE=1.2 SCNB=2.0
hL-cD-cC-nN    1    0.000    0.000   2.0    SCEE=1.2 SCNB=2.0
hN-nN-cC-oC    1    2.500  180.000   2.0    SCEE=1.2 SCNB=2.0
nN-cA-cA-oH    1    0.156    0.000   3.0    SCEE=1.2 SCNB=2.0
nN-cA-cA-oT    1    0.000    0.000   3.0    SCEE=1.2 SCNB=2.0  Lipid21 v1.0
cB-cA-oH-hO    1    0.160    0.000  -3.0    SCEE=1.2 SCNB=2.0
cB-cA-oH-hO    1    0.250    0.000   1.0    SCEE=1.2 SCNB=2.0
cB-cB-cA-oH    1    0.000    0.000   2.0    SCEE=1.2 SCNB=2.0
hE-cA-cB-cB    1    0.000    0.000   2.0    SCEE=1.2 SCNB=2.0
```

```
hE-cA-cB-hB    1    0.000    0.000   2.0    SCEE=1.2 SCNB=2.0
oH-cA-cB-hB    1    0.000    0.000   2.0    SCEE=1.2 SCNB=2.0
cC-cD-cD-cB    1    0.1556   0.000   3.0    SCEE=1.2 SCNB=2.0


cA-cA-cB-cB              1.10         180.00           2.00
cA-cB-cB-hB              1.10         180.00           2.00
cA-cC-nN-hN              1.10         180.00           2.00
cA-oO-cC-oO              1.10         180.00           2.00
cB-cD-cB-hB              1.10         180.00           2.00
cD-nN-cC-oC              1.10         180.00           2.00
cD-oC-cC-oS             10.50         180.00           2.00
cA-cC-nN-hN              1.10         180.00           2.00


  hw  ow  0000.     0000.                               4.  flag for fast water    Lipid11
v1.0  (GAFF)


MOD4      RE
  pA    2.1000   0.2000
  nA    1.8240   0.1700
  nN    1.8240   0.1700
  cA    1.9080   0.1094
  cD    1.9080   0.1094
  cB    1.9080   0.0860
  cC    1.9080   0.0860
  oC    1.6612   0.2100
  oS    1.6837   0.1700
  oP    1.6612   0.2100
  oT    1.6837   0.1700
  oO    1.6612   0.2100
  oH    1.7210   0.2104
  hA    1.4870   0.0157
  hB    1.2500   0.0070 Lipid14
  hL    1.4600   0.0100 Lipid14
  hE    1.3870   0.0157
  hX    1.1000   0.0157
  hN    0.6000   0.0157
  hO    0.6000   0.0157 Remove zero size
```

Lipid21 charges (lipid21.lib)

```
!!index array str
 "AR"
 "CHL"
 "DHA"
 "LAL"
 "MY"
 "OL"
 "PA"
 "PC"
 "PE"
 "PGR"
 "PGS"
 "PH-"
 "PS"
 "SA"
 "SPM"
 "ST"
!entry.AR.unit.atoms table  str name  str type  int typex  int resx  int flags  int seq
int elmnt  dbl chg
 "C116" "cD" 0 1 131073 1 6 0.039100
 "H16R" "hL" 0 1 131073 2 1 0.028848
 "H16S" "hL" 0 1 131073 3 1 0.028848
 "C115" "cB" 0 1 131073 4 6 -0.244149
 "H15R" "hB" 0 1 131073 5 1 0.132793
 "C114" "cB" 0 1 131073 6 6 -0.224769
 "H14R" "hB" 0 1 131073 7 1 0.129616
 "C113" "cD" 0 1 131073 8 6 0.058197
 "H13R" "hL" 0 1 131073 9 1 0.061350
 "H13S" "hL" 0 1 131073 10 1 0.061350
 "C112" "cB" 0 1 131073 11 6 -0.220164
 "H12R" "hB" 0 1 131073 12 1 0.131986
 "C111" "cB" 0 1 131073 13 6 -0.228704
 "H11R" "hB" 0 1 131073 14 1 0.131341
 "C110" "cD" 0 1 131073 15 6 0.095406
 "H10R" "hL" 0 1 131073 16 1 0.051140
 "H10S" "hL" 0 1 131073 17 1 0.051140
 "C19" "cB" 0 1 131073 18 6 -0.228694
 "H9R" "hB" 0 1 131073 19 1 0.132167
 "C18" "cB" 0 1 131073 20 6 -0.228527
 "H8R" "hB" 0 1 131073 21 1 0.134735
 "C17" "cD" 0 1 131073 22 6 0.055784
 "H7R" "hL" 0 1 131073 23 1 0.062775
 "H7S" "hL" 0 1 131073 24 1 0.062775
```

```
 "C16" "cB" 0 1 131073 25 6 -0.206786
 "H6R" "hB" 0 1 131073 26 1 0.129858
 "C15" "cB" 0 1 131073 27 6 -0.267890
 "H5R" "hB" 0 1 131073 28 1 0.143577
 "C14" "cD" 0 1 131073 29 6 0.031382
 "H4R" "hL" 0 1 131073 30 1 0.038708
 "H4S" "hL" 0 1 131073 31 1 0.038708
 "C13" "cD" 0 1 131073 32 6 0.025626
 "H3R" "hL" 0 1 131073 33 1 0.025117
 "H3S" "hL" 0 1 131073 34 1 0.025117
 "C12" "cD" 0 1 131073 35 6 -0.123935
 "H2R" "hL" 0 1 131073 36 1 0.024535
 "H2S" "hL" 0 1 131073 37 1 0.024535
 "C117" "cD" 0 1 131073 45 6 -0.028398
 "H17R" "hL" 0 1 131073 46 1 0.019581
 "H17S" "hL" 0 1 131073 47 1 0.019581
 "C118" "cD" 0 1 131073 48 6 -0.019772
 "H18R" "hL" 0 1 131073 49 1 0.014218
 "H18S" "hL" 0 1 131073 50 1 0.014218
 "C119" "cD" 0 1 131073 51 6 0.024957
 "H19R" "hL" 0 1 131073 52 1 0.005426
 "H19S" "hL" 0 1 131073 53 1 0.005426
 "C120" "cD" 0 1 131073 54 6 -0.109302
 "H20R" "hL" 0 1 131073 55 1 0.023723
 "H20S" "hL" 0 1 131073 56 1 0.023723
 "H20T" "hL" 0 1 131073 57 1 0.023723
!entry.AR.unit.atomspertinfo table  str pname  str ptype  int ptypex  int pelmnt  dbl pchg
 "C116" "cD" 0 -1 0.0
 "H16R" "hL" 0 -1 0.0
 "H16S" "hL" 0 -1 0.0
 "C115" "cB" 0 -1 0.0
 "H15R" "hB" 0 -1 0.0
 "C114" "cB" 0 -1 0.0
 "H14R" "hB" 0 -1 0.0
 "C113" "cD" 0 -1 0.0
 "H13R" "hL" 0 -1 0.0
 "H13S" "hL" 0 -1 0.0
 "C112" "cB" 0 -1 0.0
 "H12R" "hB" 0 -1 0.0
 "C111" "cB" 0 -1 0.0
 "H11R" "hB" 0 -1 0.0
 "C110" "cD" 0 -1 0.0
 "H10R" "hL" 0 -1 0.0
```

```
 "H10S" "hL" 0 -1 0.0
 "C19" "cB" 0 -1 0.0
 "H9R" "hB" 0 -1 0.0
 "C18" "cB" 0 -1 0.0
 "H8R" "hB" 0 -1 0.0
 "C17" "cD" 0 -1 0.0
 "H7R" "hL" 0 -1 0.0
 "H7S" "hL" 0 -1 0.0
 "C16" "cB" 0 -1 0.0
 "H6R" "hB" 0 -1 0.0
 "C15" "cB" 0 -1 0.0
 "H5R" "hB" 0 -1 0.0
 "C14" "cD" 0 -1 0.0
 "H4R" "hL" 0 -1 0.0
 "H4S" "hL" 0 -1 0.0
 "C13" "cD" 0 -1 0.0
 "H3R" "hL" 0 -1 0.0
 "H3S" "hL" 0 -1 0.0
 "C12" "cD" 0 -1 0.0
 "H2R" "hL" 0 -1 0.0
 "H2S" "hL" 0 -1 0.0
 "C117" "cD" 0 -1 0.0
 "H17R" "hL" 0 -1 0.0
 "H17S" "hL" 0 -1 0.0
 "C118" "cD" 0 -1 0.0
 "H18R" "hL" 0 -1 0.0
 "H18S" "hL" 0 -1 0.0
 "C119" "cD" 0 -1 0.0
 "H19R" "hL" 0 -1 0.0
 "H19S" "hL" 0 -1 0.0
 "C120" "cD" 0 -1 0.0
 "H20R" "hL" 0 -1 0.0
 "H20S" "hL" 0 -1 0.0
 "H20T" "hL" 0 -1 0.0
!entry.AR.unit.boundbox array dbl
 -1.000000
 0.0
 0.0
 0.0
 0.0
!entry.AR.unit.childsequence single int
 2
!entry.AR.unit.connect array int
```

```
 35
 35
!entry.AR.unit.connectivity table  int atom1x  int atom2x  int flags
 1 2 1
 1 3 1
 1 4 1
 1 38 1
 4 5 1
 4 6 2
 6 7 1
 6 8 1
 8 9 1
 8 10 1
 8 11 1
 11 12 1
 11 13 2
 13 14 1
 13 15 1
 15 16 1
 15 17 1
 15 18 1
 18 19 1
 18 20 2
 20 21 1
 20 22 1
 22 23 1
 22 24 1
 22 25 1
 25 26 1
 25 27 2
 27 28 1
 27 29 1
 29 30 1
 29 31 1
 29 32 1
 32 33 1
 32 34 1
 32 35 1
 35 36 1
 35 37 1
 38 39 1
 38 40 1
 38 41 1
```

```
 41 42 1
 41 43 1
 41 44 1
 44 45 1
 44 46 1
 44 47 1
 47 48 1
 47 49 1
 47 50 1
!entry.AR.unit.hierarchy table  str abovetype  int abovex  str belowtype  int belowx
 "U" 0 "R" 1
 "R" 1 "A" 1
 "R" 1 "A" 2
 "R" 1 "A" 3
 "R" 1 "A" 4
 "R" 1 "A" 5
 "R" 1 "A" 6
 "R" 1 "A" 7
 "R" 1 "A" 8
 "R" 1 "A" 9
 "R" 1 "A" 10
 "R" 1 "A" 11
 "R" 1 "A" 12
 "R" 1 "A" 13
 "R" 1 "A" 14
 "R" 1 "A" 15
 "R" 1 "A" 16
 "R" 1 "A" 17
 "R" 1 "A" 18
 "R" 1 "A" 19
 "R" 1 "A" 20
 "R" 1 "A" 21
 "R" 1 "A" 22
 "R" 1 "A" 23
 "R" 1 "A" 24
 "R" 1 "A" 25
 "R" 1 "A" 26
 "R" 1 "A" 27
 "R" 1 "A" 28
 "R" 1 "A" 29
 "R" 1 "A" 30
 "R" 1 "A" 31
 "R" 1 "A" 32
```

```
 "R" 1 "A" 33
 "R" 1 "A" 34
 "R" 1 "A" 35
 "R" 1 "A" 36
 "R" 1 "A" 37
 "R" 1 "A" 38
 "R" 1 "A" 39
 "R" 1 "A" 40
 "R" 1 "A" 41
 "R" 1 "A" 42
 "R" 1 "A" 43
 "R" 1 "A" 44
 "R" 1 "A" 45
 "R" 1 "A" 46
 "R" 1 "A" 47
 "R" 1 "A" 48
 "R" 1 "A" 49
 "R" 1 "A" 50
!entry.AR.unit.name single str
 "AR"
!entry.AR.unit.positions table  dbl x  dbl y  dbl z
 -3.542000 1.263000 -0.507000
 -4.373000 1.525000 0.147000
 -2.920000 0.574000 0.053000
 -4.106000 0.611000 -1.743000
 -4.793000 1.231000 -2.299000
 -3.871000 -0.600000 -2.218000
 -4.393000 -0.888000 -3.118000
 -2.969000 -1.670000 -1.649000
 -2.270000 -1.252000 -0.937000
 -2.374000 -2.080000 -2.465000
 -3.766000 -2.788000 -1.011000
 -4.712000 -3.005000 -1.481000
 -3.426000 -3.504000 0.046000
 -4.122000 -4.252000 0.390000
 -2.146000 -3.387000 0.847000
 -1.326000 -3.099000 0.203000
 -1.897000 -4.375000 1.231000
 -2.272000 -2.426000 2.011000
 -3.242000 -2.408000 2.482000
 -1.340000 -1.630000 2.505000
 -1.611000 -1.007000 3.344000
 0.097000 -1.490000 2.057000
```

```
 0.316000 -2.162000 1.240000
 0.737000 -1.797000 2.884000
 0.442000 -0.063000 1.702000
 0.227000 0.652000 2.481000
 0.966000 0.404000 0.581000
 1.145000 1.467000 0.528000
 1.363000 -0.350000 -0.663000
 0.783000 0.037000 -1.499000
 1.119000 -1.403000 -0.583000
 2.852000 -0.196000 -1.008000
 3.101000 0.857000 -1.091000
 3.031000 -0.640000 -1.984000
 3.775000 -0.860000 0.024000
 3.551000 -1.915000 0.111000
 3.634000 -0.397000 0.994000
 -2.747000 2.535000 -0.828000
 -1.894000 2.273000 -1.450000
 -3.364000 3.205000 -1.425000
 -2.265000 3.270000 0.423000
 -3.125000 3.537000 1.037000
 -1.659000 2.595000 1.024000
 -1.459000 4.532000 0.111000
 -0.599000 4.267000 -0.501000
 -2.064000 5.207000 -0.492000
 -0.981000 5.263000 1.365000
 -0.412000 6.152000 1.111000
 -0.345000 4.625000 1.972000
 -1.821000 5.573000 1.982000
!entry.AR.unit.residueconnect table  int c1x  int c2x  int c3x  int c4x  int c5x  int c6x
 35 35 0 0 0 0
!entry.AR.unit.residues table  str name  int seq  int childseq  int startatomx  str restype
int imagingx
 "AR" 1 58 1 "?" 0
!entry.AR.unit.residuesPdbSequenceNumber array int
 0
!entry.AR.unit.solventcap array dbl
 -1.000000
 0.0
 0.0
 0.0
 0.0
!entry.AR.unit.velocities table  dbl x  dbl y  dbl z
 0.0 0.0 0.0
```

```
0.0 0.0 0.0
0.0 0.0 0.0
0.0 0.0 0.0
0.0 0.0 0.0
0.0 0.0 0.0
0.0 0.0 0.0
0.0 0.0 0.0
0.0 0.0 0.0
0.0 0.0 0.0
0.0 0.0 0.0
0.0 0.0 0.0
0.0 0.0 0.0
0.0 0.0 0.0
0.0 0.0 0.0
0.0 0.0 0.0
0.0 0.0 0.0
0.0 0.0 0.0
0.0 0.0 0.0
0.0 0.0 0.0
0.0 0.0 0.0
0.0 0.0 0.0
0.0 0.0 0.0
0.0 0.0 0.0
0.0 0.0 0.0
0.0 0.0 0.0
0.0 0.0 0.0
0.0 0.0 0.0
0.0 0.0 0.0
0.0 0.0 0.0
0.0 0.0 0.0
0.0 0.0 0.0
0.0 0.0 0.0
0.0 0.0 0.0
0.0 0.0 0.0
0.0 0.0 0.0
0.0 0.0 0.0
0.0 0.0 0.0
0.0 0.0 0.0
0.0 0.0 0.0
0.0 0.0 0.0
0.0 0.0 0.0
0.0 0.0 0.0
0.0 0.0 0.0
```

```
 0.0 0.0 0.0
 0.0 0.0 0.0
 0.0 0.0 0.0
 0.0 0.0 0.0
 0.0 0.0 0.0
 0.0 0.0 0.0
!entry.CHL.unit.atoms table  str name  str type  int typex  int resx  int flags  int seq
int elmnt  dbl chg
 "C1" "cA" 0 1 131073 1 6 -0.247736
 "H11" "hA" 0 1 131073 2 1 0.048772
 "H12" "hA" 0 1 131073 3 1 0.048772
 "C2" "cA" 0 1 131073 4 6 -0.061498
 "H21" "hA" 0 1 131073 5 1 0.048128
 "H22" "hA" 0 1 131073 6 1 0.048128
 "C3" "cA" 0 1 131073 7 6 0.400362
 "H31" "hE" 0 1 131073 8 1 -0.008510
 "C4" "cA" 0 1 131073 9 6 -0.237134
 "H41" "hA" 0 1 131073 10 1 0.100619
 "H42" "hA" 0 1 131073 11 1 0.100619
 "C5" "cB" 0 1 131073 12 6 -0.257515
 "C6" "cB" 0 1 131073 13 6 -0.243730
 "H61" "hB" 0 1 131073 14 1 0.148607
 "C7" "cA" 0 1 131073 15 6 -0.075453
 "H71" "hA" 0 1 131073 16 1 0.051297
 "H72" "hA" 0 1 131073 17 1 0.051297
 "C8" "cA" 0 1 131073 18 6 0.017835
 "H81" "hA" 0 1 131073 19 1 0.028120
 "C9" "cA" 0 1 131073 20 6 -0.029839
 "H91" "hA" 0 1 131073 21 1 -0.003361
 "C10" "cA" 0 1 131073 22 6 0.546470
 "C11" "cA" 0 1 131073 23 6 -0.127715
 "H111" "hA" 0 1 131073 24 1 0.040257
 "H112" "hA" 0 1 131073 25 1 0.040257
 "C12" "cA" 0 1 131073 26 6 -0.158054
 "H121" "hA" 0 1 131073 27 1 0.007687
 "H122" "hA" 0 1 131073 28 1 0.007687
 "C13" "cA" 0 1 131073 29 6 0.583692
 "C14" "cA" 0 1 131073 30 6 0.013753
 "H141" "hA" 0 1 131073 31 1 0.004469
 "C15" "cA" 0 1 131073 32 6 -0.185047
 "H151" "hA" 0 1 131073 33 1 0.041228
 "H152" "hA" 0 1 131073 34 1 0.041228
 "C16" "cA" 0 1 131073 35 6 -0.082919
```

```
 "H161" "hA" 0 1 131073 36 1 0.027639
 "H162" "hA" 0 1 131073 37 1 0.027639
 "C17" "cA" 0 1 131073 38 6 -0.051767
 "H171" "hA" 0 1 131073 39 1 -0.030436
 "C18" "cA" 0 1 131073 40 6 -0.536004
 "H181" "hA" 0 1 131073 41 1 0.103623
 "H182" "hA" 0 1 131073 42 1 0.103623
 "H183" "hA" 0 1 131073 43 1 0.103623
 "C19" "cA" 0 1 131073 44 6 -0.352218
 "H191" "hA" 0 1 131073 45 1 0.072469
 "H192" "hA" 0 1 131073 46 1 0.072469
 "H193" "hA" 0 1 131073 47 1 0.072469
 "C20" "cD" 0 1 131075 48 6 0.178752
 "H201" "hL" 0 1 131075 49 1 -0.016015
 "C21" "cD" 0 1 131075 50 6 -0.427968
 "H211" "hL" 0 1 131075 51 1 0.099219
 "H212" "hL" 0 1 131075 52 1 0.099219
 "H213" "hL" 0 1 131075 53 1 0.099219
 "C22" "cD" 0 1 131075 54 6 -0.050256
 "H221" "hL" 0 1 131075 55 1 0.009445
 "H222" "hL" 0 1 131075 56 1 0.009445
 "C23" "cD" 0 1 131075 57 6 0.078665
 "H231" "hL" 0 1 131075 58 1 -0.007957
 "H232" "hL" 0 1 131075 59 1 -0.007957
 "C24" "cD" 0 1 131075 60 6 -0.282133
 "H241" "hL" 0 1 131075 61 1 0.062972
 "H242" "hL" 0 1 131075 62 1 0.062972
 "C25" "cD" 0 1 131075 63 6 0.450601
 "H251" "hL" 0 1 131075 64 1 -0.049128
 "C26" "cD" 0 1 131075 65 6 -0.449300
 "H261" "hL" 0 1 131075 66 1 0.099869
 "H262" "hL" 0 1 131075 67 1 0.099869
 "H263" "hL" 0 1 131075 68 1 0.099869
 "C27" "cD" 0 1 131075 69 6 -0.449300
 "H271" "hL" 0 1 131075 70 1 0.099869
 "H272" "hL" 0 1 131075 71 1 0.099869
 "H273" "hL" 0 1 131075 72 1 0.099869
 "O1" "oH" 0 1 131073 73 8 -0.766581
 "HO1" "hO" 0 1 131073 74 1 0.442970
!entry.CHL.unit.atomspertinfo table  str pname  str ptype  int ptypex  int pelmnt  dbl pchg
 "C1" "cA" 0 -1 0.0
 "H11" "hA" 0 -1 0.0
 "H12" "hA" 0 -1 0.0
```

```
"C2" "cA" 0 -1 0.0
"H21" "hA" 0 -1 0.0
"H22" "hA" 0 -1 0.0
"C3" "cA" 0 -1 0.0
"H31" "hE" 0 -1 0.0
"C4" "cA" 0 -1 0.0
"H41" "hA" 0 -1 0.0
"H42" "hA" 0 -1 0.0
"C5" "cB" 0 -1 0.0
"C6" "cB" 0 -1 0.0
"H61" "hB" 0 -1 0.0
"C7" "cA" 0 -1 0.0
"H71" "hA" 0 -1 0.0
"H72" "hA" 0 -1 0.0
"C8" "cA" 0 -1 0.0
"H81" "hA" 0 -1 0.0
"C9" "cA" 0 -1 0.0
"H91" "hA" 0 -1 0.0
"C10" "cA" 0 -1 0.0
"C11" "cA" 0 -1 0.0
"H111" "hA" 0 -1 0.0
"H112" "hA" 0 -1 0.0
"C12" "cA" 0 -1 0.0
"H121" "hA" 0 -1 0.0
"H122" "hA" 0 -1 0.0
"C13" "cA" 0 -1 0.0
"C14" "cA" 0 -1 0.0
"H141" "hA" 0 -1 0.0
"C15" "cA" 0 -1 0.0
"H151" "hA" 0 -1 0.0
"H152" "hA" 0 -1 0.0
"C16" "cA" 0 -1 0.0
"H161" "hA" 0 -1 0.0
"H162" "hA" 0 -1 0.0
"C17" "cA" 0 -1 0.0
"H171" "hA" 0 -1 0.0
"C18" "cA" 0 -1 0.0
"H181" "hA" 0 -1 0.0
"H182" "hA" 0 -1 0.0
"H183" "hA" 0 -1 0.0
"C19" "cA" 0 -1 0.0
"H191" "hA" 0 -1 0.0
"H192" "hA" 0 -1 0.0
```

```
 "H193" "hA" 0 -1 0.0
 "C20" "cA" 0 -1 0.0
 "H201" "hA" 0 -1 0.0
 "C21" "cA" 0 -1 0.0
 "H211" "hA" 0 -1 0.0
 "H212" "hA" 0 -1 0.0
 "H213" "hA" 0 -1 0.0
 "C22" "cA" 0 -1 0.0
 "H221" "hA" 0 -1 0.0
 "H222" "hA" 0 -1 0.0
 "C23" "cA" 0 -1 0.0
 "H231" "hA" 0 -1 0.0
 "H232" "hA" 0 -1 0.0
 "C24" "cA" 0 -1 0.0
 "H241" "hA" 0 -1 0.0
 "H242" "hA" 0 -1 0.0
 "C25" "cA" 0 -1 0.0
 "H251" "hA" 0 -1 0.0
 "C26" "cA" 0 -1 0.0
 "H261" "hA" 0 -1 0.0
 "H262" "hA" 0 -1 0.0
 "H263" "hA" 0 -1 0.0
 "C27" "cA" 0 -1 0.0
 "H271" "hA" 0 -1 0.0
 "H272" "hA" 0 -1 0.0
 "H273" "hA" 0 -1 0.0
 "O1" "oH" 0 -1 0.0
 "HO1" "hO" 0 -1 0.0
!entry.CHL.unit.boundbox array dbl
 -1.000000
 0.0
 0.0
 0.0
 0.0
!entry.CHL.unit.childsequence single int
 2
!entry.CHL.unit.connect array int
 0
 0
!entry.CHL.unit.connectivity table  int atom1x  int atom2x  int flags
 1 2 1
 1 3 1
 1 4 1
```

1 22 1
4 5 1
4 6 1
4 7 1
7 8 1
7 9 1
7 73 1
9 10 1
9 11 1
9 12 1
12 13 2
12 22 1
13 14 1
13 15 1
15 16 1
15 17 1
15 18 1
18 19 1
18 20 1
18 30 1
20 21 1
20 22 1
20 23 1
22 44 1
23 24 1
23 25 1
23 26 1
26 27 1
26 28 1
26 29 1
29 30 1
29 38 1
29 40 1
30 31 1
30 32 1
32 33 1
32 34 1
32 35 1
35 36 1
35 37 1
35 38 1
38 39 1
38 48 1

```
 40 41 1
 40 42 1
 40 43 1
 44 45 1
 44 46 1
 44 47 1
 48 49 1
 48 50 1
 48 54 1
 50 51 1
 50 52 1
 50 53 1
 54 55 1
 54 56 1
 54 57 1
 57 58 1
 57 59 1
 57 60 1
 60 61 1
 60 62 1
 60 63 1
 63 64 1
 63 65 1
 63 69 1
 65 66 1
 65 67 1
 65 68 1
 69 70 1
 69 71 1
 69 72 1
 73 74 1
!entry.CHL.unit.hierarchy table  str abovetype  int abovex  str belowtype  int belowx
 "U" 0 "R" 1
 "R" 1 "A" 1
 "R" 1 "A" 2
 "R" 1 "A" 3
 "R" 1 "A" 4
 "R" 1 "A" 5
 "R" 1 "A" 6
 "R" 1 "A" 7
 "R" 1 "A" 8
 "R" 1 "A" 9
 "R" 1 "A" 10
```

"R" 1 "A" 11
"R" 1 "A" 12
"R" 1 "A" 13
"R" 1 "A" 14
"R" 1 "A" 15
"R" 1 "A" 16
"R" 1 "A" 17
"R" 1 "A" 18
"R" 1 "A" 19
"R" 1 "A" 20
"R" 1 "A" 21
"R" 1 "A" 22
"R" 1 "A" 23
"R" 1 "A" 24
"R" 1 "A" 25
"R" 1 "A" 26
"R" 1 "A" 27
"R" 1 "A" 28
"R" 1 "A" 29
"R" 1 "A" 30
"R" 1 "A" 31
"R" 1 "A" 32
"R" 1 "A" 33
"R" 1 "A" 34
"R" 1 "A" 35
"R" 1 "A" 36
"R" 1 "A" 37
"R" 1 "A" 38
"R" 1 "A" 39
"R" 1 "A" 40
"R" 1 "A" 41
"R" 1 "A" 42
"R" 1 "A" 43
"R" 1 "A" 44
"R" 1 "A" 45
"R" 1 "A" 46
"R" 1 "A" 47
"R" 1 "A" 48
"R" 1 "A" 49
"R" 1 "A" 50
"R" 1 "A" 51
"R" 1 "A" 52
"R" 1 "A" 53

```
 "R" 1 "A" 54
 "R" 1 "A" 55
 "R" 1 "A" 56
 "R" 1 "A" 57
 "R" 1 "A" 58
 "R" 1 "A" 59
 "R" 1 "A" 60
 "R" 1 "A" 61
 "R" 1 "A" 62
 "R" 1 "A" 63
 "R" 1 "A" 64
 "R" 1 "A" 65
 "R" 1 "A" 66
 "R" 1 "A" 67
 "R" 1 "A" 68
 "R" 1 "A" 69
 "R" 1 "A" 70
 "R" 1 "A" 71
 "R" 1 "A" 72
 "R" 1 "A" 73
 "R" 1 "A" 74
!entry.CHL.unit.name single str
 "CHL"
!entry.CHL.unit.positions table  dbl x  dbl y  dbl z
 4.697000 -1.469000 -0.739000
 4.428000 -1.214000 -1.762000
 4.303000 -2.462000 -0.556000
 6.224000 -1.517000 -0.642000
 6.547000 -1.874000 0.331000
 6.617000 -2.214000 -1.376000
 6.830000 -0.145000 -0.877000
 6.590000 0.176000 -1.891000
 6.246000 0.863000 0.111000
 6.601000 0.589000 1.102000
 6.636000 1.856000 -0.095000
 4.731000 0.895000 0.070000
 4.091000 2.041000 -0.088000
 4.668000 2.946000 -0.204000
 2.599000 2.206000 -0.119000
 2.323000 3.070000 0.480000
 2.290000 2.443000 -1.137000
 1.853000 0.964000 0.375000
 1.922000 0.946000 1.460000
```

2.514000 -0.306000 -0.204000
2.518000 -0.165000 -1.285000
4.012000 -0.452000 0.214000
1.671000 -1.566000 0.076000
2.085000 -2.410000 -0.467000
1.730000 -1.829000 1.125000
0.190000 -1.429000 -0.312000
0.108000 -1.322000 -1.392000
-0.312000 -2.355000 -0.056000
-0.469000 -0.212000 0.358000
0.381000 1.015000 -0.041000
0.381000 1.020000 -1.132000
-0.462000 2.215000 0.391000
-0.304000 2.443000 1.442000
-0.217000 3.115000 -0.163000
-1.913000 1.751000 0.131000
-2.537000 1.930000 1.001000
-2.357000 2.309000 -0.684000
-1.859000 0.228000 -0.203000
-1.794000 0.127000 -1.286000
-0.546000 -0.407000 1.886000
-1.062000 0.412000 2.374000
0.431000 -0.485000 2.344000
-1.086000 -1.316000 2.130000
4.156000 -0.937000 1.675000
3.579000 -0.319000 2.354000
5.185000 -0.898000 2.008000
3.826000 -1.963000 1.788000
-3.141000 -0.519000 0.237000
-3.267000 -0.372000 1.309000
-3.080000 -2.029000 -0.029000
-2.330000 -2.520000 0.577000
-2.851000 -2.232000 -1.072000
-4.025000 -2.508000 0.198000
-4.375000 0.093000 -0.461000
-4.324000 1.175000 -0.403000
-4.337000 -0.153000 -1.522000
-5.732000 -0.335000 0.111000
-5.862000 -1.406000 0.010000
-5.752000 -0.121000 1.179000
-6.898000 0.389000 -0.569000
-6.718000 1.461000 -0.516000
-6.910000 0.137000 -1.629000

```
 -8.286000 0.104000 0.028000
 -8.244000 0.333000 1.092000
 -8.705000 -1.362000 -0.122000
 -8.034000 -2.035000 0.400000
 -8.723000 -1.655000 -1.169000
 -9.702000 -1.521000 0.280000
 -9.336000 1.023000 -0.605000
 -9.079000 2.069000 -0.470000
 -10.316000 0.862000 -0.166000
 -9.420000 0.839000 -1.674000
 8.224000 -0.262000 -0.734000
 8.633000 0.570000 -0.924000
!entry.CHL.unit.residueconnect table  int c1x  int c2x  int c3x  int c4x  int c5x  int c6x
 0 0 0 0 0 0
!entry.CHL.unit.residues table  str name  int seq  int childseq  int startatomx  str
restype  int imagingx
 "CHL" 1 75 1 "?" 0
!entry.CHL.unit.residuesPdbSequenceNumber array int
 0
!entry.CHL.unit.solventcap array dbl
 -1.000000
 0.0
 0.0
 0.0
 0.0
!entry.CHL.unit.velocities table  dbl x  dbl y  dbl z
 0.0 0.0 0.0
 0.0 0.0 0.0
 0.0 0.0 0.0
 0.0 0.0 0.0
 0.0 0.0 0.0
 0.0 0.0 0.0
 0.0 0.0 0.0
 0.0 0.0 0.0
 0.0 0.0 0.0
 0.0 0.0 0.0
 0.0 0.0 0.0
 0.0 0.0 0.0
 0.0 0.0 0.0
 0.0 0.0 0.0
 0.0 0.0 0.0
 0.0 0.0 0.0
 0.0 0.0 0.0
```

```
0.0 0.0 0.0
0.0 0.0 0.0
0.0 0.0 0.0
0.0 0.0 0.0
0.0 0.0 0.0
0.0 0.0 0.0
0.0 0.0 0.0
0.0 0.0 0.0
0.0 0.0 0.0
0.0 0.0 0.0
0.0 0.0 0.0
0.0 0.0 0.0
0.0 0.0 0.0
0.0 0.0 0.0
0.0 0.0 0.0
0.0 0.0 0.0
0.0 0.0 0.0
0.0 0.0 0.0
0.0 0.0 0.0
0.0 0.0 0.0
0.0 0.0 0.0
0.0 0.0 0.0
0.0 0.0 0.0
0.0 0.0 0.0
0.0 0.0 0.0
0.0 0.0 0.0
0.0 0.0 0.0
0.0 0.0 0.0
0.0 0.0 0.0
0.0 0.0 0.0
0.0 0.0 0.0
0.0 0.0 0.0
0.0 0.0 0.0
0.0 0.0 0.0
0.0 0.0 0.0
0.0 0.0 0.0
0.0 0.0 0.0
0.0 0.0 0.0
0.0 0.0 0.0
0.0 0.0 0.0
0.0 0.0 0.0
0.0 0.0 0.0
0.0 0.0 0.0
```

```
 0.0 0.0 0.0
 0.0 0.0 0.0
 0.0 0.0 0.0
 0.0 0.0 0.0
 0.0 0.0 0.0
 0.0 0.0 0.0
 0.0 0.0 0.0
 0.0 0.0 0.0
 0.0 0.0 0.0
 0.0 0.0 0.0
 0.0 0.0 0.0
 0.0 0.0 0.0
 0.0 0.0 0.0
 0.0 0.0 0.0
!entry.DHA.unit.atoms table  str name  str type  int typex  int resx  int flags  int seq
int elmnt  dbl chg
 "C116" "cB" 0 1 131073 1 6 -0.233897
 "H16R" "hB" 0 1 131073 2 1 0.133812
 "C115" "cD" 0 1 131073 3 6 0.069215
 "H15R" "hL" 0 1 131073 4 1 0.058341
 "H15S" "hL" 0 1 131073 5 1 0.058341
 "C114" "cB" 0 1 131073 6 6 -0.240419
 "H14R" "hB" 0 1 131073 7 1 0.138337
 "C113" "cB" 0 1 131073 8 6 -0.218668
 "H13R" "hB" 0 1 131073 9 1 0.131575
 "C112" "cD" 0 1 131073 10 6 0.085245
 "H12R" "hL" 0 1 131073 11 1 0.053422
 "H12S" "hL" 0 1 131073 12 1 0.053422
 "C111" "cB" 0 1 131073 13 6 -0.241205
 "H11R" "hB" 0 1 131073 14 1 0.136308
 "C110" "cB" 0 1 131073 15 6 -0.221433
 "H10R" "hB" 0 1 131073 16 1 0.133628
 "C19" "cD" 0 1 131073 17 6 0.089043
 "H9R" "hL" 0 1 131073 18 1 0.054255
 "H9S" "hL" 0 1 131073 19 1 0.054255
 "C18" "cB" 0 1 131073 20 6 -0.226254
 "H8R" "hB" 0 1 131073 21 1 0.128147
 "C17" "cB" 0 1 131073 22 6 -0.220895
 "H7R" "hB" 0 1 131073 23 1 0.139863
 "C16" "cD" 0 1 131073 24 6 0.060337
 "H6R" "hL" 0 1 131073 25 1 0.063515
 "H6S" "hL" 0 1 131073 26 1 0.063515
 "C15" "cB" 0 1 131073 27 6 -0.214845
```

```
 "H5R" "hB" 0 1 131073 28 1 0.132504
 "C14" "cB" 0 1 131073 29 6 -0.287059
 "H4R" "hB" 0 1 131073 30 1 0.154038
 "C13" "cD" 0 1 131073 31 6 0.120999
 "H3R" "hL" 0 1 131073 32 1 0.026097
 "H3S" "hL" 0 1 131073 33 1 0.026097
 "C12" "cD" 0 1 131073 34 6 -0.142937
 "H2R" "hL" 0 1 131073 35 1 0.037544
 "H2S" "hL" 0 1 131073 36 1 0.037544
 "C117" "cB" 0 1 131073 44 6 -0.216499
 "H17R" "hB" 0 1 131073 45 1 0.132121
 "C118" "cD" 0 1 131073 46 6 0.092718
 "H18R" "hL" 0 1 131073 47 1 0.053227
 "H18S" "hL" 0 1 131073 48 1 0.053227
 "C119" "cB" 0 1 131073 49 6 -0.246833
 "H19R" "hB" 0 1 131073 50 1 0.136323
 "C120" "cB" 0 1 131073 51 6 -0.240216
 "H20R" "hB" 0 1 131073 52 1 0.133048
 "C121" "cD" 0 1 131073 53 6 0.122022
 "H21R" "hL" 0 1 131073 54 1 0.010042
 "H21S" "hL" 0 1 131073 55 1 0.010042
 "C122" "cD" 0 1 131073 56 6 -0.108913
 "H22R" "hL" 0 1 131073 57 1 0.025968
 "H22S" "hL" 0 1 131073 58 1 0.025968
 "H22T" "hL" 0 1 131073 59 1 0.025968
!entry.DHA.unit.atomspertinfo table  str pname  str ptype  int ptypex  int pelmnt  dbl pchg
 "C116" "cB" 0 -1 0.0
 "H16R" "hB" 0 -1 0.0
 "C115" "cD" 0 -1 0.0
 "H15R" "hL" 0 -1 0.0
 "H15S" "hL" 0 -1 0.0
 "C114" "cB" 0 -1 0.0
 "H14R" "hB" 0 -1 0.0
 "C113" "cB" 0 -1 0.0
 "H13R" "hB" 0 -1 0.0
 "C112" "cD" 0 -1 0.0
 "H12R" "hL" 0 -1 0.0
 "H12S" "hL" 0 -1 0.0
 "C111" "cB" 0 -1 0.0
 "H11R" "hB" 0 -1 0.0
 "C110" "cB" 0 -1 0.0
 "H10R" "hB" 0 -1 0.0
 "C19" "cD" 0 -1 0.0
```

```
 "H9R" "hL" 0 -1 0.0
 "H9S" "hL" 0 -1 0.0
 "C18" "cB" 0 -1 0.0
 "H8R" "hB" 0 -1 0.0
 "C17" "cB" 0 -1 0.0
 "H7R" "hB" 0 -1 0.0
 "C16" "cD" 0 -1 0.0
 "H6R" "hL" 0 -1 0.0
 "H6S" "hL" 0 -1 0.0
 "C15" "cB" 0 -1 0.0
 "H5R" "hB" 0 -1 0.0
 "C14" "cB" 0 -1 0.0
 "H4R" "hB" 0 -1 0.0
 "C13" "cD" 0 -1 0.0
 "H3R" "hL" 0 -1 0.0
 "H3S" "hL" 0 -1 0.0
 "C12" "cD" 0 -1 0.0
 "H2R" "hL" 0 -1 0.0
 "H2S" "hL" 0 -1 0.0
 "C117" "cB" 0 -1 0.0
 "H17R" "hB" 0 -1 0.0
 "C118" "cD" 0 -1 0.0
 "H18R" "hL" 0 -1 0.0
 "H18S" "hL" 0 -1 0.0
 "C119" "cB" 0 -1 0.0
 "H19R" "hB" 0 -1 0.0
 "C120" "cB" 0 -1 0.0
 "H20R" "hB" 0 -1 0.0
 "C121" "cD" 0 -1 0.0
 "H21R" "hL" 0 -1 0.0
 "H21S" "hL" 0 -1 0.0
 "C122" "cD" 0 -1 0.0
 "H22R" "hL" 0 -1 0.0
 "H22S" "hL" 0 -1 0.0
 "H22T" "hL" 0 -1 0.0
!entry.DHA.unit.boundbox array dbl
 -1.000000
 0.0
 0.0
 0.0
 0.0
!entry.DHA.unit.childsequence single int
 2
```

```
!entry.DHA.unit.connect array int
 34
 34
!entry.DHA.unit.connectivity table  int atom1x  int atom2x  int flags
 1 2 1
 1 3 1
 1 37 2
 3 4 1
 3 5 1
 3 6 1
 6 7 1
 6 8 2
 8 9 1
 8 10 1
 10 11 1
 10 12 1
 10 13 1
 13 14 1
 13 15 2
 15 16 1
 15 17 1
 17 18 1
 17 19 1
 17 20 1
 20 21 1
 20 22 2
 22 23 1
 22 24 1
 24 25 1
 24 26 1
 24 27 1
 27 28 1
 27 29 2
 29 30 1
 29 31 1
 31 32 1
 31 33 1
 31 34 1
 34 35 1
 34 36 1
 37 38 1
 37 39 1
 39 40 1
```

```
 39 41 1
 39 42 1
 42 43 1
 42 44 2
 44 45 1
 44 46 1
 46 47 1
 46 48 1
 46 49 1
 49 50 1
 49 51 1
 49 52 1
!entry.DHA.unit.hierarchy table  str abovetype  int abovex  str belowtype  int belowx
 "U" 0 "R" 1
 "R" 1 "A" 1
 "R" 1 "A" 2
 "R" 1 "A" 3
 "R" 1 "A" 4
 "R" 1 "A" 5
 "R" 1 "A" 6
 "R" 1 "A" 7
 "R" 1 "A" 8
 "R" 1 "A" 9
 "R" 1 "A" 10
 "R" 1 "A" 11
 "R" 1 "A" 12
 "R" 1 "A" 13
 "R" 1 "A" 14
 "R" 1 "A" 15
 "R" 1 "A" 16
 "R" 1 "A" 17
 "R" 1 "A" 18
 "R" 1 "A" 19
 "R" 1 "A" 20
 "R" 1 "A" 21
 "R" 1 "A" 22
 "R" 1 "A" 23
 "R" 1 "A" 24
 "R" 1 "A" 25
 "R" 1 "A" 26
 "R" 1 "A" 27
 "R" 1 "A" 28
 "R" 1 "A" 29
```

```
 "R" 1 "A" 30
 "R" 1 "A" 31
 "R" 1 "A" 32
 "R" 1 "A" 33
 "R" 1 "A" 34
 "R" 1 "A" 35
 "R" 1 "A" 36
 "R" 1 "A" 37
 "R" 1 "A" 38
 "R" 1 "A" 39
 "R" 1 "A" 40
 "R" 1 "A" 41
 "R" 1 "A" 42
 "R" 1 "A" 43
 "R" 1 "A" 44
 "R" 1 "A" 45
 "R" 1 "A" 46
 "R" 1 "A" 47
 "R" 1 "A" 48
 "R" 1 "A" 49
 "R" 1 "A" 50
 "R" 1 "A" 51
 "R" 1 "A" 52
!entry.DHA.unit.name single str
 "DHA"
!entry.DHA.unit.positions table  dbl x  dbl y  dbl z
 6.186000 0.815000 -0.603000
 5.772000 1.811000 -0.619000
 5.152000 -0.282000 -0.706000
 5.627000 -1.257000 -0.734000
 4.618000 -0.174000 -1.644000
 4.194000 -0.242000 0.462000
 4.661000 -0.419000 1.419000
 2.893000 -0.010000 0.455000
 2.380000 -0.028000 1.404000
 1.993000 0.270000 -0.727000
 2.572000 0.350000 -1.640000
 1.515000 1.232000 -0.581000
 0.961000 -0.820000 -0.898000
 1.380000 -1.795000 -1.094000
 -0.357000 -0.729000 -0.828000
 -0.925000 -1.634000 -0.972000
 -1.204000 0.493000 -0.557000
```

```
 -1.860000 0.659000 -1.404000
 -0.584000 1.379000 -0.470000
 -2.001000 0.333000 0.716000
 -1.397000 0.236000 1.605000
 -3.314000 0.284000 0.867000
 -3.695000 0.166000 1.869000
 -4.387000 0.387000 -0.193000
 -4.997000 -0.509000 -0.162000
 -3.948000 0.424000 -1.183000
 -5.236000 1.620000 0.008000
 -4.695000 2.548000 -0.092000
 -6.524000 1.695000 0.297000
 -6.951000 2.681000 0.401000
 -7.505000 0.570000 0.499000
 -7.983000 0.686000 1.467000
 -7.007000 -0.392000 0.505000
 -8.592000 0.571000 -0.593000
 -8.148000 0.420000 -1.567000
 -9.110000 1.525000 -0.592000
 7.499000 0.703000 -0.489000
 8.069000 1.616000 -0.419000
 8.338000 -0.552000 -0.428000
 7.719000 -1.435000 -0.548000
 9.032000 -0.549000 -1.261000
 9.079000 -0.651000 0.885000
 8.435000 -0.701000 1.750000
 10.384000 -0.673000 1.094000
 10.715000 -0.743000 2.120000
 11.504000 -0.603000 0.088000
 12.102000 -1.509000 0.169000
 11.121000 -0.579000 -0.925000
 12.411000 0.611000 0.320000
 13.235000 0.621000 -0.387000
 12.834000 0.599000 1.320000
 11.857000 1.537000 0.206000
!entry.DHA.unit.residueconnect table  int c1x  int c2x  int c3x  int c4x  int c5x  int c6x
 34 34 0 0 0 0
!entry.DHA.unit.residues table  str name  int seq  int childseq  int startatomx  str
restype  int imagingx
 "DHA" 1 60 1 "?" 0
!entry.DHA.unit.residuesPdbSequenceNumber array int
 0
!entry.DHA.unit.solventcap array dbl
```

```
 -1.000000
 0.0
 0.0
 0.0
 0.0
!entry.DHA.unit.velocities table  dbl x  dbl y  dbl z
 0.0 0.0 0.0
 0.0 0.0 0.0
 0.0 0.0 0.0
 0.0 0.0 0.0
 0.0 0.0 0.0
 0.0 0.0 0.0
 0.0 0.0 0.0
 0.0 0.0 0.0
 0.0 0.0 0.0
 0.0 0.0 0.0
 0.0 0.0 0.0
 0.0 0.0 0.0
 0.0 0.0 0.0
 0.0 0.0 0.0
 0.0 0.0 0.0
 0.0 0.0 0.0
 0.0 0.0 0.0
 0.0 0.0 0.0
 0.0 0.0 0.0
 0.0 0.0 0.0
 0.0 0.0 0.0
 0.0 0.0 0.0
 0.0 0.0 0.0
 0.0 0.0 0.0
 0.0 0.0 0.0
 0.0 0.0 0.0
 0.0 0.0 0.0
 0.0 0.0 0.0
 0.0 0.0 0.0
 0.0 0.0 0.0
 0.0 0.0 0.0
 0.0 0.0 0.0
 0.0 0.0 0.0
 0.0 0.0 0.0
 0.0 0.0 0.0
 0.0 0.0 0.0
 0.0 0.0 0.0
```

```
 0.0 0.0 0.0
 0.0 0.0 0.0
 0.0 0.0 0.0
 0.0 0.0 0.0
 0.0 0.0 0.0
 0.0 0.0 0.0
 0.0 0.0 0.0
 0.0 0.0 0.0
 0.0 0.0 0.0
 0.0 0.0 0.0
 0.0 0.0 0.0
 0.0 0.0 0.0
 0.0 0.0 0.0
 0.0 0.0 0.0
 0.0 0.0 0.0
!entry.LAL.unit.atoms table  str name  str type  int typex  int resx  int flags  int seq
int elmnt  dbl chg
 "H12T" "hL" 0 1 131073 1 1 0.025809
 "C112" "cD" 0 1 131073 2 6 -0.118534
 "H12R" "hL" 0 1 131073 3 1 0.025809
 "H12S" "hL" 0 1 131073 4 1 0.025809
 "C111" "cD" 0 1 131073 5 6 0.023320
 "H11R" "hL" 0 1 131073 6 1 0.008276
 "H11S" "hL" 0 1 131073 7 1 0.008276
 "C110" "cD" 0 1 131073 8 6 -0.019575
 "H10R" "hL" 0 1 131073 9 1 0.015163
 "H10S" "hL" 0 1 131073 10 1 0.015163
 "C19" "cD" 0 1 131073 11 6 -0.021975
 "H9R" "hL" 0 1 131073 12 1 0.005869
 "H9S" "hL" 0 1 131073 13 1 0.005869
 "C18" "cD" 0 1 131073 14 6 -0.020879
 "H8R" "hL" 0 1 131073 15 1 0.010385
 "H8S" "hL" 0 1 131073 16 1 0.010385
 "C17" "cD" 0 1 131073 17 6 -0.019279
 "H7R" "hL" 0 1 131073 18 1 0.011207
 "H7S" "hL" 0 1 131073 19 1 0.011207
 "C16" "cD" 0 1 131073 20 6 -0.019631
 "H6R" "hL" 0 1 131073 21 1 0.010236
 "H6S" "hL" 0 1 131073 22 1 0.010236
 "C15" "cD" 0 1 131073 23 6 -0.015735
 "H5R" "hL" 0 1 131073 24 1 0.009551
 "H5S" "hL" 0 1 131073 25 1 0.009551
 "C14" "cD" 0 1 131073 26 6 -0.024078
```

```
 "H4R" "hL" 0 1 131073 27 1 0.020580
 "H4S" "hL" 0 1 131073 28 1 0.020580
 "C13" "cD" 0 1 131073 29 6 0.001510
 "H3R" "hL" 0 1 131073 30 1 0.019108
 "H3S" "hL" 0 1 131073 31 1 0.019108
 "C12" "cD" 0 1 131073 32 6 -0.172159
 "H2R" "hL" 0 1 131073 33 1 0.054419
 "H2S" "hL" 0 1 131073 34 1 0.054419
!entry.LAL.unit.atomspertinfo table  str pname  str ptype  int ptypex  int pelmnt  dbl pchg
 "H12T" "hL" 0 -1 0.0
 "C112" "cD" 0 -1 0.0
 "H12R" "hL" 0 -1 0.0
 "H12S" "hL" 0 -1 0.0
 "C111" "cD" 0 -1 0.0
 "H11R" "hL" 0 -1 0.0
 "H11S" "hL" 0 -1 0.0
 "C110" "cD" 0 -1 0.0
 "H10R" "hL" 0 -1 0.0
 "H10S" "hL" 0 -1 0.0
 "C19" "cD" 0 -1 0.0
 "H9R" "hL" 0 -1 0.0
 "H9S" "hL" 0 -1 0.0
 "C18" "cD" 0 -1 0.0
 "H8R" "hL" 0 -1 0.0
 "H8S" "hL" 0 -1 0.0
 "C17" "cD" 0 -1 0.0
 "H7R" "hL" 0 -1 0.0
 "H7S" "hL" 0 -1 0.0
 "C16" "cD" 0 -1 0.0
 "H6R" "hL" 0 -1 0.0
 "H6S" "hL" 0 -1 0.0
 "C15" "cD" 0 -1 0.0
 "H5R" "hL" 0 -1 0.0
 "H5S" "hL" 0 -1 0.0
 "C14" "cD" 0 -1 0.0
 "H4R" "hL" 0 -1 0.0
 "H4S" "hL" 0 -1 0.0
 "C13" "cD" 0 -1 0.0
 "H3R" "hL" 0 -1 0.0
 "H3S" "hL" 0 -1 0.0
 "C12" "cD" 0 -1 0.0
 "H2R" "hL" 0 -1 0.0
 "H2S" "hL" 0 -1 0.0
```

!entry.LAL.unit.boundbox array dbl
-1.000000
0.0
0.0
0.0
0.0
!entry.LAL.unit.childsequence single int
2
!entry.LAL.unit.connect array int
32
32
!entry.LAL.unit.connectivity table int atom1x int atom2x int flags
1 2 1
2 3 1
2 4 1
2 5 1
5 8 1
5 7 1
5 6 1
8 11 1
8 10 1
8 9 1
11 14 1
11 13 1
11 12 1
14 17 1
14 16 1
14 15 1
17 20 1
17 19 1
17 18 1
20 23 1
20 22 1
20 21 1
23 26 1
23 25 1
23 24 1
26 29 1
26 28 1
26 27 1
29 32 1
29 30 1
29 31 1

```
 32 34 1
 32 33 1
!entry.LAL.unit.hierarchy table  str abovetype  int abovex  str belowtype  int belowx
 "U" 0 "R" 1
 "R" 1 "A" 1
 "R" 1 "A" 2
 "R" 1 "A" 3
 "R" 1 "A" 4
 "R" 1 "A" 5
 "R" 1 "A" 6
 "R" 1 "A" 7
 "R" 1 "A" 8
 "R" 1 "A" 9
 "R" 1 "A" 10
 "R" 1 "A" 11
 "R" 1 "A" 12
 "R" 1 "A" 13
 "R" 1 "A" 14
 "R" 1 "A" 15
 "R" 1 "A" 16
 "R" 1 "A" 17
 "R" 1 "A" 18
 "R" 1 "A" 19
 "R" 1 "A" 20
 "R" 1 "A" 21
 "R" 1 "A" 22
 "R" 1 "A" 23
 "R" 1 "A" 24
 "R" 1 "A" 25
 "R" 1 "A" 26
 "R" 1 "A" 27
 "R" 1 "A" 28
 "R" 1 "A" 29
 "R" 1 "A" 30
 "R" 1 "A" 31
 "R" 1 "A" 32
 "R" 1 "A" 33
 "R" 1 "A" 34
!entry.LAL.unit.name single str
 "LAL"
!entry.LAL.unit.positions table  dbl x  dbl y  dbl z
 72.456000 10.844000 19.346000
 71.871000 10.231000 18.815000
```

71.178000 9.870000 19.578000
72.578000 9.424000 18.604000
71.063000 10.566000 17.542000
71.736000 10.898000 16.748000
70.381000 11.389000 17.765000
70.248000 9.346000 17.062000
69.657000 8.960000 17.896000
70.930000 8.552000 16.748000
69.295000 9.709000 15.900000
69.867000 10.082000 15.047000
68.620000 10.502000 16.225000
68.458000 8.485000 15.479000
67.960000 8.078000 16.362000
69.123000 7.713000 15.091000
67.396000 8.833000 14.413000
67.887000 9.155000 13.492000
66.788000 9.669000 14.765000
66.494000 7.608000 14.129000
66.260000 7.108000 15.071000
67.042000 6.887000 13.518000
65.152000 7.963000 13.447000
64.552000 8.556000 14.137000
64.602000 7.038000 13.264000
65.314000 8.740000 12.120000
66.094000 8.276000 11.515000
65.638000 9.759000 12.335000
64.012000 8.783000 11.285000
63.755000 7.770000 10.968000
64.207000 9.367000 10.384000
62.815000 9.404000 12.036000
63.101000 10.354000 12.488000
62.489000 8.734000 12.830000
!entry.LAL.unit.residueconnect table int c1x int c2x int c3x int c4x int c5x int c6x
32 32 0 0 0 0
!entry.LAL.unit.residues table str name int seq int childseq int startatomx str restype int imagingx
"LAL" 1 35 1 "?" 0
!entry.LAL.unit.residuesPdbSequenceNumber array int
1
!entry.LAL.unit.solventcap array dbl
-1.000000
0.0
0.0

```
 0.0
 0.0
!entry.LAL.unit.velocities table  dbl x  dbl y  dbl z
 0.0 0.0 0.0
 0.0 0.0 0.0
 0.0 0.0 0.0
 0.0 0.0 0.0
 0.0 0.0 0.0
 0.0 0.0 0.0
 0.0 0.0 0.0
 0.0 0.0 0.0
 0.0 0.0 0.0
 0.0 0.0 0.0
 0.0 0.0 0.0
 0.0 0.0 0.0
 0.0 0.0 0.0
 0.0 0.0 0.0
 0.0 0.0 0.0
 0.0 0.0 0.0
 0.0 0.0 0.0
 0.0 0.0 0.0
 0.0 0.0 0.0
 0.0 0.0 0.0
 0.0 0.0 0.0
 0.0 0.0 0.0
 0.0 0.0 0.0
 0.0 0.0 0.0
 0.0 0.0 0.0
 0.0 0.0 0.0
 0.0 0.0 0.0
 0.0 0.0 0.0
 0.0 0.0 0.0
 0.0 0.0 0.0
 0.0 0.0 0.0
 0.0 0.0 0.0
 0.0 0.0 0.0
!entry.MY.unit.atoms table  str name  str type  int typex  int resx  int flags  int seq
int elmnt  dbl chg
 "H14T" "hL" 0 1 131073 1 1 0.025511
 "C114" "cD" 0 1 131073 2 6 -0.111712
 "H14R" "hL" 0 1 131073 3 1 0.025511
 "H14S" "hL" 0 1 131073 4 1 0.025511
```

```
 "C113" "cD" 0 1 131073 5 6 0.017463
 "H13R" "hL" 0 1 131073 6 1 0.007531
 "H13S" "hL" 0 1 131073 7 1 0.007531
 "C112" "cD" 0 1 131073 8 6 -0.028094
 "H12R" "hL" 0 1 131073 9 1 0.017208
 "H12S" "hL" 0 1 131073 10 1 0.017208
 "C111" "cD" 0 1 131073 11 6 -0.031787
 "H11R" "hL" 0 1 131073 12 1 0.012677
 "H11S" "hL" 0 1 131073 13 1 0.012677
 "C110" "cD" 0 1 131073 14 6 -0.021025
 "H10R" "hL" 0 1 131073 15 1 0.010037
 "H10S" "hL" 0 1 131073 16 1 0.010037
 "C19" "cD" 0 1 131073 17 6 -0.014653
 "H9R" "hL" 0 1 131073 18 1 0.010172
 "H9S" "hL" 0 1 131073 19 1 0.010172
 "C18" "cD" 0 1 131073 20 6 -0.026451
 "H8R" "hL" 0 1 131073 21 1 0.011241
 "H8S" "hL" 0 1 131073 22 1 0.011241
 "C17" "cD" 0 1 131073 23 6 -0.004386
 "H7R" "hL" 0 1 131073 24 1 0.006833
 "H7S" "hL" 0 1 131073 25 1 0.006833
 "C16" "cD" 0 1 131073 26 6 -0.027812
 "H6R" "hL" 0 1 131073 27 1 0.011511
 "H6S" "hL" 0 1 131073 28 1 0.011511
 "C15" "cD" 0 1 131073 29 6 -0.016521
 "H5R" "hL" 0 1 131073 30 1 0.010573
 "H5S" "hL" 0 1 131073 31 1 0.010573
 "C14" "cD" 0 1 131073 32 6 -0.026954
 "H4R" "hL" 0 1 131073 33 1 0.020431
 "H4S" "hL" 0 1 131073 34 1 0.020431
 "C13" "cD" 0 1 131073 35 6 -0.002435
 "H3R" "hL" 0 1 131073 36 1 0.018859
 "H3S" "hL" 0 1 131073 37 1 0.018859
 "C12" "cD" 0 1 131073 38 6 -0.164200
 "H2R" "hL" 0 1 131073 39 1 0.053944
 "H2S" "hL" 0 1 131073 40 1 0.053944
!entry.MY.unit.atomspertinfo table  str pname  str ptype  int ptypex  int pelmnt  dbl pchg
 "H14T" "hL" 0 -1 0.0
 "C114" "cD" 0 -1 0.0
 "H14R" "hL" 0 -1 0.0
 "H14S" "hL" 0 -1 0.0
 "C113" "cD" 0 -1 0.0
 "H13R" "hL" 0 -1 0.0
```

```
 "H13S" "hL" 0 -1 0.0
 "C112" "cD" 0 -1 0.0
 "H12R" "hL" 0 -1 0.0
 "H12S" "hL" 0 -1 0.0
 "C111" "cD" 0 -1 0.0
 "H11R" "hL" 0 -1 0.0
 "H11S" "hL" 0 -1 0.0
 "C110" "cD" 0 -1 0.0
 "H10R" "hL" 0 -1 0.0
 "H10S" "hL" 0 -1 0.0
 "C19" "cD" 0 -1 0.0
 "H9R" "hL" 0 -1 0.0
 "H9S" "hL" 0 -1 0.0
 "C18" "cD" 0 -1 0.0
 "H8R" "hL" 0 -1 0.0
 "H8S" "hL" 0 -1 0.0
 "C17" "cD" 0 -1 0.0
 "H7R" "hL" 0 -1 0.0
 "H7S" "hL" 0 -1 0.0
 "C16" "cD" 0 -1 0.0
 "H6R" "hL" 0 -1 0.0
 "H6S" "hL" 0 -1 0.0
 "C15" "cD" 0 -1 0.0
 "H5R" "hL" 0 -1 0.0
 "H5S" "hL" 0 -1 0.0
 "C14" "cD" 0 -1 0.0
 "H4R" "hL" 0 -1 0.0
 "H4S" "hL" 0 -1 0.0
 "C13" "cD" 0 -1 0.0
 "H3R" "hL" 0 -1 0.0
 "H3S" "hL" 0 -1 0.0
 "C12" "cD" 0 -1 0.0
 "H2R" "hL" 0 -1 0.0
 "H2S" "hL" 0 -1 0.0
!entry.MY.unit.boundbox array dbl
 -1.000000
 0.0
 0.0
 0.0
 0.0
!entry.MY.unit.childsequence single int
 2
!entry.MY.unit.connect array int
```

```
 38
 38
!entry.MY.unit.connectivity table  int atom1x  int atom2x  int flags
 1 2 1
 2 5 1
 2 3 1
 2 4 1
 5 8 1
 5 6 1
 5 7 1
 8 11 1
 8 10 1
 8 9 1
 11 14 1
 11 12 1
 11 13 1
 14 17 1
 14 16 1
 14 15 1
 17 20 1
 17 18 1
 17 19 1
 20 23 1
 20 22 1
 20 21 1
 23 26 1
 23 24 1
 23 25 1
 26 29 1
 26 27 1
 26 28 1
 29 32 1
 29 30 1
 29 31 1
 32 35 1
 32 34 1
 32 33 1
 35 38 1
 35 37 1
 35 36 1
 38 40 1
 38 39 1
!entry.MY.unit.hierarchy table  str abovetype  int abovex  str belowtype  int belowx
```

```
 "U" 0 "R" 1
 "R" 1 "A" 1
 "R" 1 "A" 2
 "R" 1 "A" 3
 "R" 1 "A" 4
 "R" 1 "A" 5
 "R" 1 "A" 6
 "R" 1 "A" 7
 "R" 1 "A" 8
 "R" 1 "A" 9
 "R" 1 "A" 10
 "R" 1 "A" 11
 "R" 1 "A" 12
 "R" 1 "A" 13
 "R" 1 "A" 14
 "R" 1 "A" 15
 "R" 1 "A" 16
 "R" 1 "A" 17
 "R" 1 "A" 18
 "R" 1 "A" 19
 "R" 1 "A" 20
 "R" 1 "A" 21
 "R" 1 "A" 22
 "R" 1 "A" 23
 "R" 1 "A" 24
 "R" 1 "A" 25
 "R" 1 "A" 26
 "R" 1 "A" 27
 "R" 1 "A" 28
 "R" 1 "A" 29
 "R" 1 "A" 30
 "R" 1 "A" 31
 "R" 1 "A" 32
 "R" 1 "A" 33
 "R" 1 "A" 34
 "R" 1 "A" 35
 "R" 1 "A" 36
 "R" 1 "A" 37
 "R" 1 "A" 38
 "R" 1 "A" 39
 "R" 1 "A" 40
!entry.MY.unit.name single str
 "MY"
```

```
!entry.MY.unit.positions table  dbl x  dbl y  dbl z
 72.992000 10.982000 21.559000
 73.387000 11.208000 20.669000
 74.081000 10.373000 20.543000
 73.988000 12.092000 20.900000
 72.635000 11.469000 19.341000
 73.359000 11.785000 18.585000
 71.940000 12.299000 19.482000
 71.871000 10.231000 18.815000
 71.178000 9.870000 19.578000
 72.578000 9.424000 18.604000
 71.063000 10.566000 17.542000
 71.736000 10.898000 16.748000
 70.381000 11.389000 17.765000
 70.248000 9.346000 17.062000
 69.657000 8.960000 17.896000
 70.930000 8.552000 16.748000
 69.295000 9.709000 15.900000
 69.867000 10.082000 15.047000
 68.620000 10.502000 16.225000
 68.458000 8.485000 15.479000
 67.960000 8.078000 16.362000
 69.123000 7.713000 15.091000
 67.396000 8.833000 14.413000
 67.887000 9.155000 13.492000
 66.788000 9.669000 14.765000
 66.494000 7.608000 14.129000
 66.260000 7.108000 15.071000
 67.042000 6.887000 13.518000
 65.152000 7.963000 13.447000
 64.552000 8.556000 14.137000
 64.602000 7.038000 13.264000
 65.314000 8.740000 12.120000
 66.094000 8.276000 11.515000
 65.638000 9.759000 12.335000
 64.012000 8.783000 11.285000
 63.755000 7.770000 10.968000
 64.207000 9.367000 10.384000
 62.815000 9.404000 12.036000
 63.101000 10.354000 12.488000
 62.489000 8.734000 12.830000
!entry.MY.unit.residueconnect table  int c1x  int c2x  int c3x  int c4x  int c5x  int c6x
 38 38 0 0 0 0
```

```
!entry.MY.unit.residues table  str name  int seq  int childseq  int startatomx  str restype
int imagingx
 "MY" 1 41 1 "?" 0
!entry.MY.unit.residuesPdbSequenceNumber array int
 1
!entry.MY.unit.solventcap array dbl
 -1.000000
 0.0
 0.0
 0.0
 0.0
!entry.MY.unit.velocities table  dbl x  dbl y  dbl z
 0.0 0.0 0.0
 0.0 0.0 0.0
 0.0 0.0 0.0
 0.0 0.0 0.0
 0.0 0.0 0.0
 0.0 0.0 0.0
 0.0 0.0 0.0
 0.0 0.0 0.0
 0.0 0.0 0.0
 0.0 0.0 0.0
 0.0 0.0 0.0
 0.0 0.0 0.0
 0.0 0.0 0.0
 0.0 0.0 0.0
 0.0 0.0 0.0
 0.0 0.0 0.0
 0.0 0.0 0.0
 0.0 0.0 0.0
 0.0 0.0 0.0
 0.0 0.0 0.0
 0.0 0.0 0.0
 0.0 0.0 0.0
 0.0 0.0 0.0
 0.0 0.0 0.0
 0.0 0.0 0.0
 0.0 0.0 0.0
 0.0 0.0 0.0
 0.0 0.0 0.0
 0.0 0.0 0.0
 0.0 0.0 0.0
 0.0 0.0 0.0
```

```
 0.0 0.0 0.0
 0.0 0.0 0.0
 0.0 0.0 0.0
 0.0 0.0 0.0
 0.0 0.0 0.0
 0.0 0.0 0.0
 0.0 0.0 0.0
 0.0 0.0 0.0
 0.0 0.0 0.0
!entry.OL.unit.atoms table  str name  str type  int typex  int resx  int flags  int seq
int elmnt  dbl chg
 "C12" "cD" 0 1 131073 8 6 -0.143418
 "H2R" "hL" 0 1 131073 9 1 0.042861
 "H2S" "hL" 0 1 131073 10 1 0.042861
 "C13" "cD" 0 1 131073 11 6 0.001619
 "H3R" "hL" 0 1 131073 12 1 0.019529
 "H3S" "hL" 0 1 131073 13 1 0.019529
 "C14" "cD" 0 1 131073 14 6 -0.022960
 "H4R" "hL" 0 1 131073 15 1 0.018134
 "H4S" "hL" 0 1 131073 16 1 0.018134
 "C15" "cD" 0 1 131073 17 6 -0.016692
 "H5R" "hL" 0 1 131073 18 1 0.008690
 "H5S" "hL" 0 1 131073 19 1 0.008690
 "C16" "cD" 0 1 131073 20 6 -0.023789
 "H6R" "hL" 0 1 131073 21 1 0.009266
 "H6S" "hL" 0 1 131073 22 1 0.009266
 "C17" "cD" 0 1 131073 23 6 -0.017495
 "H7R" "hL" 0 1 131073 24 1 0.019598
 "H7S" "hL" 0 1 131073 25 1 0.019598
 "C18" "cD" 0 1 131073 26 6 0.031775
 "H8R" "hL" 0 1 131073 27 1 0.032685
 "H8S" "hL" 0 1 131073 28 1 0.032685
 "C19" "cB" 0 1 131073 29 6 -0.244228
 "H9R" "hB" 0 1 131073 30 1 0.131512
 "C110" "cB" 0 1 131073 31 6 -0.239284
 "H10R" "hB" 0 1 131073 32 1 0.126878
 "C111" "cD" 0 1 131073 33 6 0.029059
 "H11R" "hL" 0 1 131073 34 1 0.033531
 "H11S" "hL" 0 1 131073 35 1 0.033531
 "C112" "cD" 0 1 131073 36 6 -0.026353
 "H12R" "hL" 0 1 131073 37 1 0.019717
 "H12S" "hL" 0 1 131073 38 1 0.019717
 "C113" "cD" 0 1 131073 39 6 -0.018257
```

```
 "H13R" "hL" 0 1 131073 40 1 0.013800
 "H13S" "hL" 0 1 131073 41 1 0.013800
 "C114" "cD" 0 1 131073 42 6 -0.024849
 "H14R" "hL" 0 1 131073 43 1 0.008572
 "H14S" "hL" 0 1 131073 44 1 0.008572
 "C115" "cD" 0 1 131073 45 6 -0.023247
 "H15R" "hL" 0 1 131073 46 1 0.010403
 "H15S" "hL" 0 1 131073 47 1 0.010403
 "C116" "cD" 0 1 131073 48 6 -0.012488
 "H16R" "hL" 0 1 131073 49 1 0.013659
 "H16S" "hL" 0 1 131073 50 1 0.013659
 "C117" "cD" 0 1 131073 51 6 0.008684
 "H17R" "hL" 0 1 131073 52 1 0.010026
 "H17S" "hL" 0 1 131073 53 1 0.010026
 "C118" "cD" 0 1 131073 54 6 -0.117290
 "H18R" "hL" 0 1 131073 55 1 0.026627
 "H18S" "hL" 0 1 131073 56 1 0.026627
 "H18T" "hL" 0 1 131073 57 1 0.026627
!entry.OL.unit.atomspertinfo table  str pname  str ptype  int ptypex  int pelmnt  dbl pchg
 "C12" "cD" 0 -1 0.0
 "H2R" "hL" 0 -1 0.0
 "H2S" "hL" 0 -1 0.0
 "C13" "cD" 0 -1 0.0
 "H3R" "hL" 0 -1 0.0
 "H3S" "hL" 0 -1 0.0
 "C14" "cD" 0 -1 0.0
 "H4R" "hL" 0 -1 0.0
 "H4S" "hL" 0 -1 0.0
 "C15" "cD" 0 -1 0.0
 "H5R" "hL" 0 -1 0.0
 "H5S" "hL" 0 -1 0.0
 "C16" "cD" 0 -1 0.0
 "H6R" "hL" 0 -1 0.0
 "H6S" "hL" 0 -1 0.0
 "C17" "cD" 0 -1 0.0
 "H7R" "hL" 0 -1 0.0
 "H7S" "hL" 0 -1 0.0
 "C18" "cD" 0 -1 0.0
 "H8R" "hL" 0 -1 0.0
 "H8S" "hL" 0 -1 0.0
 "C19" "cB" 0 -1 0.0
 "H9R" "hB" 0 -1 0.0
 "C110" "cB" 0 -1 0.0
```

```
 "H10R" "hB" 0 -1 0.0
 "C111" "cD" 0 -1 0.0
 "H11R" "hL" 0 -1 0.0
 "H11S" "hL" 0 -1 0.0
 "C112" "cD" 0 -1 0.0
 "H12R" "hL" 0 -1 0.0
 "H12S" "hL" 0 -1 0.0
 "C113" "cD" 0 -1 0.0
 "H13R" "hL" 0 -1 0.0
 "H13S" "hL" 0 -1 0.0
 "C114" "cD" 0 -1 0.0
 "H14R" "hL" 0 -1 0.0
 "H14S" "hL" 0 -1 0.0
 "C115" "cD" 0 -1 0.0
 "H15R" "hL" 0 -1 0.0
 "H15S" "hL" 0 -1 0.0
 "C116" "cD" 0 -1 0.0
 "H16R" "hL" 0 -1 0.0
 "H16S" "hL" 0 -1 0.0
 "C117" "cD" 0 -1 0.0
 "H17R" "hL" 0 -1 0.0
 "H17S" "hL" 0 -1 0.0
 "C118" "cD" 0 -1 0.0
 "H18R" "hL" 0 -1 0.0
 "H18S" "hL" 0 -1 0.0
 "H18T" "hL" 0 -1 0.0
!entry.OL.unit.boundbox array dbl
 -1.000000
 0.0
 0.0
 0.0
 0.0
!entry.OL.unit.childsequence single int
 2
!entry.OL.unit.connect array int
 1
 1
!entry.OL.unit.connectivity table  int atom1x  int atom2x  int flags
 1 2 1
 1 3 1
 1 4 1
 4 5 1
 4 6 1
```

```
4 7 1
7 8 1
7 9 1
7 10 1
10 11 1
10 12 1
10 13 1
13 14 1
13 15 1
13 16 1
16 17 1
16 18 1
16 19 1
19 20 1
19 21 1
19 22 1
22 23 1
22 24 2
24 25 1
24 26 1
26 27 1
26 28 1
26 29 1
29 30 1
29 31 1
29 32 1
32 33 1
32 34 1
32 35 1
35 36 1
35 37 1
35 38 1
38 39 1
38 40 1
38 41 1
41 42 1
41 43 1
41 44 1
44 45 1
44 46 1
44 47 1
47 48 1
47 49 1
```

```
 47 50 1
!entry.OL.unit.hierarchy table  str abovetype  int abovex  str belowtype  int belowx
 "U" 0 "R" 1
 "R" 1 "A" 1
 "R" 1 "A" 2
 "R" 1 "A" 3
 "R" 1 "A" 4
 "R" 1 "A" 5
 "R" 1 "A" 6
 "R" 1 "A" 7
 "R" 1 "A" 8
 "R" 1 "A" 9
 "R" 1 "A" 10
 "R" 1 "A" 11
 "R" 1 "A" 12
 "R" 1 "A" 13
 "R" 1 "A" 14
 "R" 1 "A" 15
 "R" 1 "A" 16
 "R" 1 "A" 17
 "R" 1 "A" 18
 "R" 1 "A" 19
 "R" 1 "A" 20
 "R" 1 "A" 21
 "R" 1 "A" 22
 "R" 1 "A" 23
 "R" 1 "A" 24
 "R" 1 "A" 25
 "R" 1 "A" 26
 "R" 1 "A" 27
 "R" 1 "A" 28
 "R" 1 "A" 29
 "R" 1 "A" 30
 "R" 1 "A" 31
 "R" 1 "A" 32
 "R" 1 "A" 33
 "R" 1 "A" 34
 "R" 1 "A" 35
 "R" 1 "A" 36
 "R" 1 "A" 37
 "R" 1 "A" 38
 "R" 1 "A" 39
 "R" 1 "A" 40
```

```
 "R" 1 "A" 41
 "R" 1 "A" 42
 "R" 1 "A" 43
 "R" 1 "A" 44
 "R" 1 "A" 45
 "R" 1 "A" 46
 "R" 1 "A" 47
 "R" 1 "A" 48
 "R" 1 "A" 49
 "R" 1 "A" 50
!entry.OL.unit.name single str
 "OL"
!entry.OL.unit.positions table  dbl x  dbl y  dbl z
 6.166000 -0.064000 0.159000
 5.945000 -0.958000 0.733000
 5.869000 0.773000 0.786000
 5.422000 -0.043000 -1.175000
 5.748000 0.824000 -1.739000
 5.713000 -0.909000 -1.761000
 3.897000 -0.002000 -1.029000
 3.618000 0.876000 -0.452000
 3.467000 0.136000 -2.018000
 3.285000 -1.260000 -0.401000
 3.676000 -2.132000 -0.922000
 3.604000 -1.356000 0.634000
 1.753000 -1.313000 -0.454000
 1.427000 -1.233000 -1.489000
 1.431000 -2.294000 -0.110000
 1.047000 -0.244000 0.385000
 1.408000 -0.296000 1.411000
 1.297000 0.749000 0.022000
 -0.480000 -0.400000 0.376000
 -0.842000 -0.344000 -0.644000
 -0.733000 -1.396000 0.737000
 -1.164000 0.618000 1.252000
 -0.917000 0.539000 2.300000
 -2.000000 1.581000 0.901000
 -2.380000 2.214000 1.690000
 -2.510000 1.937000 -0.472000
 -2.194000 2.954000 -0.698000
 -2.066000 1.305000 -1.233000
 -4.042000 1.873000 -0.585000
 -4.484000 2.503000 0.186000
```

```
 -4.337000 2.308000 -1.538000
 -4.615000 0.459000 -0.479000
 -4.172000 -0.164000 -1.256000
 -4.321000 0.017000 0.469000
 -6.139000 0.419000 -0.609000
 -6.575000 1.054000 0.159000
 -6.433000 0.851000 -1.564000
 -6.708000 -0.999000 -0.500000
 -6.207000 -1.632000 -1.230000
 -6.458000 -1.413000 0.473000
 -8.220000 -1.104000 -0.743000
 -8.435000 -0.751000 -1.750000
 -8.499000 -2.156000 -0.726000
 -9.120000 -0.346000 0.242000
 -8.919000 0.721000 0.187000
 -10.151000 -0.470000 -0.082000
 -9.002000 -0.812000 1.694000
 -9.724000 -0.300000 2.323000
 -8.017000 -0.616000 2.104000
 -9.188000 -1.879000 1.778000
!entry.OL.unit.residueconnect table  int c1x  int c2x  int c3x  int c4x  int c5x  int c6x
 1 1 0 0 0 0
!entry.OL.unit.residues table  str name  int seq  int childseq  int startatomx  str restype
int imagingx
 "OL" 1 58 1 "?" 0
!entry.OL.unit.residuesPdbSequenceNumber array int
 0
!entry.OL.unit.solventcap array dbl
 -1.000000
 0.0
 0.0
 0.0
 0.0
!entry.OL.unit.velocities table  dbl x  dbl y  dbl z
 0.0 0.0 0.0
 0.0 0.0 0.0
 0.0 0.0 0.0
 0.0 0.0 0.0
 0.0 0.0 0.0
 0.0 0.0 0.0
 0.0 0.0 0.0
 0.0 0.0 0.0
 0.0 0.0 0.0
```

```
 0.0 0.0 0.0
 0.0 0.0 0.0
 0.0 0.0 0.0
 0.0 0.0 0.0
 0.0 0.0 0.0
 0.0 0.0 0.0
 0.0 0.0 0.0
 0.0 0.0 0.0
 0.0 0.0 0.0
 0.0 0.0 0.0
 0.0 0.0 0.0
 0.0 0.0 0.0
 0.0 0.0 0.0
 0.0 0.0 0.0
 0.0 0.0 0.0
 0.0 0.0 0.0
 0.0 0.0 0.0
 0.0 0.0 0.0
 0.0 0.0 0.0
 0.0 0.0 0.0
 0.0 0.0 0.0
 0.0 0.0 0.0
 0.0 0.0 0.0
 0.0 0.0 0.0
 0.0 0.0 0.0
 0.0 0.0 0.0
 0.0 0.0 0.0
 0.0 0.0 0.0
 0.0 0.0 0.0
 0.0 0.0 0.0
 0.0 0.0 0.0
 0.0 0.0 0.0
 0.0 0.0 0.0
 0.0 0.0 0.0
 0.0 0.0 0.0
 0.0 0.0 0.0
 0.0 0.0 0.0
 0.0 0.0 0.0
 0.0 0.0 0.0
 0.0 0.0 0.0
 0.0 0.0 0.0
!entry.PA.unit.atoms table  str name  str type  int typex  int resx  int flags  int seq
int elmnt  dbl chg
```

"C116" "cD" 0 1 131073 1 6 -0.125447
"H16R" "hL" 0 1 131073 2 1 0.029047
"H16S" "hL" 0 1 131073 3 1 0.029047
"H16T" "hL" 0 1 131073 4 1 0.029047
"C115" "cD" 0 1 131073 5 6 0.013975
"H15R" "hL" 0 1 131073 6 1 0.009292
"H15S" "hL" 0 1 131073 7 1 0.009292
"C114" "cD" 0 1 131073 8 6 -0.020086
"H14R" "hL" 0 1 131073 9 1 0.015929
"H14S" "hL" 0 1 131073 10 1 0.015929
"C113" "cD" 0 1 131073 11 6 -0.033096
"H13R" "hL" 0 1 131073 12 1 0.014621
"H13S" "hL" 0 1 131073 13 1 0.014621
"C112" "cD" 0 1 131073 14 6 -0.027633
"H12R" "hL" 0 1 131073 15 1 0.011368
"H12S" "hL" 0 1 131073 16 1 0.011368
"C111" "cD" 0 1 131073 17 6 -0.025206
"H11R" "hL" 0 1 131073 18 1 0.014334
"H11S" "hL" 0 1 131073 19 1 0.014334
"C110" "cD" 0 1 131073 20 6 -0.028831
"H10R" "hL" 0 1 131073 21 1 0.014691
"H10S" "hL" 0 1 131073 22 1 0.014691
"C19" "cD" 0 1 131073 23 6 -0.030472
"H9R" "hL" 0 1 131073 24 1 0.013897
"H9S" "hL" 0 1 131073 25 1 0.013897
"C18" "cD" 0 1 131073 26 6 -0.015793
"H8R" "hL" 0 1 131073 27 1 0.009067
"H8S" "hL" 0 1 131073 28 1 0.009067
"C17" "cD" 0 1 131073 29 6 -0.019630
"H7R" "hL" 0 1 131073 30 1 0.011041
"H7S" "hL" 0 1 131073 31 1 0.011041
"C16" "cD" 0 1 131073 32 6 -0.024427
"H6R" "hL" 0 1 131073 33 1 0.013334
"H6S" "hL" 0 1 131073 34 1 0.013334
"C15" "cD" 0 1 131073 35 6 -0.029387
"H5R" "hL" 0 1 131073 36 1 0.016763
"H5S" "hL" 0 1 131073 37 1 0.016763
"C14" "cD" 0 1 131073 38 6 -0.030099
"H4R" "hL" 0 1 131073 39 1 0.020877
"H4S" "hL" 0 1 131073 40 1 0.020877
"C13" "cD" 0 1 131073 41 6 -0.004882
"H3R" "hL" 0 1 131073 42 1 0.019007
"H3S" "hL" 0 1 131073 43 1 0.019007

```
 "C12" "cD" 0 1 131073 44 6 -0.149259
 "H2R" "hL" 0 1 131073 45 1 0.047345
 "H2S" "hL" 0 1 131073 46 1 0.047345
!entry.PA.unit.atomspertinfo table  str pname  str ptype  int ptypex  int pelmnt  dbl pchg
 "C116" "cD" 0 -1 0.0
 "H16R" "hL" 0 -1 0.0
 "H16S" "hL" 0 -1 0.0
 "H16T" "hL" 0 -1 0.0
 "C115" "cD" 0 -1 0.0
 "H15R" "hL" 0 -1 0.0
 "H15S" "hL" 0 -1 0.0
 "C114" "cD" 0 -1 0.0
 "H14R" "hL" 0 -1 0.0
 "H14S" "hL" 0 -1 0.0
 "C113" "cD" 0 -1 0.0
 "H13R" "hL" 0 -1 0.0
 "H13S" "hL" 0 -1 0.0
 "C112" "cD" 0 -1 0.0
 "H12R" "hL" 0 -1 0.0
 "H12S" "hL" 0 -1 0.0
 "C111" "cD" 0 -1 0.0
 "H11R" "hL" 0 -1 0.0
 "H11S" "hL" 0 -1 0.0
 "C110" "cD" 0 -1 0.0
 "H10R" "hL" 0 -1 0.0
 "H10S" "hL" 0 -1 0.0
 "C19" "cD" 0 -1 0.0
 "H9R" "hL" 0 -1 0.0
 "H9S" "hL" 0 -1 0.0
 "C18" "cD" 0 -1 0.0
 "H8R" "hL" 0 -1 0.0
 "H8S" "hL" 0 -1 0.0
 "C17" "cD" 0 -1 0.0
 "H7R" "hL" 0 -1 0.0
 "H7S" "hL" 0 -1 0.0
 "C16" "cD" 0 -1 0.0
 "H6R" "hL" 0 -1 0.0
 "H6S" "hL" 0 -1 0.0
 "C15" "cD" 0 -1 0.0
 "H5R" "hL" 0 -1 0.0
 "H5S" "hL" 0 -1 0.0
 "C14" "cD" 0 -1 0.0
 "H4R" "hL" 0 -1 0.0
```

```
 "H4S" "hL" 0 -1 0.0
 "C13" "cD" 0 -1 0.0
 "H3R" "hL" 0 -1 0.0
 "H3S" "hL" 0 -1 0.0
 "C12" "cD" 0 -1 0.0
 "H2R" "hL" 0 -1 0.0
 "H2S" "hL" 0 -1 0.0
!entry.PA.unit.boundbox array dbl
 -1.000000
 0.0
 0.0
 0.0
 0.0
!entry.PA.unit.childsequence single int
 2
!entry.PA.unit.connect array int
 44
 44
!entry.PA.unit.connectivity table  int atom1x  int atom2x  int flags
 1 2 1
 1 3 1
 1 4 1
 1 5 1
 5 6 1
 5 7 1
 5 8 1
 8 9 1
 8 10 1
 8 11 1
 11 12 1
 11 13 1
 11 14 1
 14 15 1
 14 16 1
 14 17 1
 17 18 1
 17 19 1
 17 20 1
 20 21 1
 20 22 1
 20 23 1
 23 24 1
 23 25 1
```

```
 23 26 1
 26 27 1
 26 28 1
 26 29 1
 29 30 1
 29 31 1
 29 32 1
 32 33 1
 32 34 1
 32 35 1
 35 36 1
 35 37 1
 35 38 1
 38 39 1
 38 40 1
 38 41 1
 41 42 1
 41 43 1
 41 44 1
 44 45 1
 44 46 1
!entry.PA.unit.hierarchy table  str abovetype  int abovex  str belowtype  int belowx
 "U" 0 "R" 1
 "R" 1 "A" 1
 "R" 1 "A" 2
 "R" 1 "A" 3
 "R" 1 "A" 4
 "R" 1 "A" 5
 "R" 1 "A" 6
 "R" 1 "A" 7
 "R" 1 "A" 8
 "R" 1 "A" 9
 "R" 1 "A" 10
 "R" 1 "A" 11
 "R" 1 "A" 12
 "R" 1 "A" 13
 "R" 1 "A" 14
 "R" 1 "A" 15
 "R" 1 "A" 16
 "R" 1 "A" 17
 "R" 1 "A" 18
 "R" 1 "A" 19
 "R" 1 "A" 20
```

```
 "R" 1 "A" 21
 "R" 1 "A" 22
 "R" 1 "A" 23
 "R" 1 "A" 24
 "R" 1 "A" 25
 "R" 1 "A" 26
 "R" 1 "A" 27
 "R" 1 "A" 28
 "R" 1 "A" 29
 "R" 1 "A" 30
 "R" 1 "A" 31
 "R" 1 "A" 32
 "R" 1 "A" 33
 "R" 1 "A" 34
 "R" 1 "A" 35
 "R" 1 "A" 36
 "R" 1 "A" 37
 "R" 1 "A" 38
 "R" 1 "A" 39
 "R" 1 "A" 40
 "R" 1 "A" 41
 "R" 1 "A" 42
 "R" 1 "A" 43
 "R" 1 "A" 44
 "R" 1 "A" 45
 "R" 1 "A" 46
!entry.PA.unit.name single str
 "PA"
!entry.PA.unit.positions table  dbl x  dbl y  dbl z
 -10.262000 -0.302000 -0.549000
 -10.644000 -0.891000 -1.377000
 -10.859000 0.603000 -0.483000
 -10.424000 -0.870000 0.363000
 -8.781000 0.025000 -0.737000
 -8.648000 0.570000 -1.670000
 -8.224000 -0.902000 -0.843000
 -8.209000 0.854000 0.416000
 -8.302000 0.290000 1.344000
 -8.824000 1.743000 0.541000
 -6.751000 1.293000 0.235000
 -6.500000 1.986000 1.035000
 -6.660000 1.857000 -0.692000
 -5.730000 0.153000 0.237000
```

```
 -5.920000 -0.519000 -0.596000
 -5.856000 -0.439000 1.142000
 -4.284000 0.648000 0.155000
 -4.084000 1.312000 0.994000
 -4.160000 1.248000 -0.744000
 -3.256000 -0.485000 0.151000
 -3.455000 -1.148000 -0.689000
 -3.381000 -1.087000 1.050000
 -1.811000 0.010000 0.072000
 -1.612000 0.673000 0.913000
 -1.685000 0.612000 -0.826000
 -0.782000 -1.123000 0.068000
 -0.983000 -1.787000 -0.770000
 -0.906000 -1.724000 0.968000
 0.663000 -0.628000 -0.013000
 0.854000 0.045000 0.820000
 0.790000 -0.036000 -0.919000
 1.685000 -1.769000 -0.010000
 1.433000 -2.463000 -0.809000
 1.595000 -2.331000 0.918000
 3.142000 -1.328000 -0.195000
 3.227000 -0.783000 -1.132000
 3.759000 -2.218000 -0.304000
 3.698000 -0.485000 0.958000
 3.490000 -0.997000 1.896000
 3.179000 0.468000 1.010000
 5.207000 -0.229000 0.884000
 5.739000 -1.174000 0.848000
 5.519000 0.273000 1.796000
 5.627000 0.634000 -0.318000
 5.083000 1.570000 -0.321000
 5.426000 0.112000 -1.246000
!entry.PA.unit.residueconnect table  int c1x  int c2x  int c3x  int c4x  int c5x  int c6x
 44 44 0 0 0 0
!entry.PA.unit.residues table  str name  int seq  int childseq  int startatomx  str restype
int imagingx
 "PA" 1 54 1 "?" 0
!entry.PA.unit.residuesPdbSequenceNumber array int
 0
!entry.PA.unit.solventcap array dbl
 -1.000000
 0.0
 0.0
```

```
 0.0
 0.0
!entry.PA.unit.velocities table  dbl x  dbl y  dbl z
 0.0 0.0 0.0
 0.0 0.0 0.0
 0.0 0.0 0.0
 0.0 0.0 0.0
 0.0 0.0 0.0
 0.0 0.0 0.0
 0.0 0.0 0.0
 0.0 0.0 0.0
 0.0 0.0 0.0
 0.0 0.0 0.0
 0.0 0.0 0.0
 0.0 0.0 0.0
 0.0 0.0 0.0
 0.0 0.0 0.0
 0.0 0.0 0.0
 0.0 0.0 0.0
 0.0 0.0 0.0
 0.0 0.0 0.0
 0.0 0.0 0.0
 0.0 0.0 0.0
 0.0 0.0 0.0
 0.0 0.0 0.0
 0.0 0.0 0.0
 0.0 0.0 0.0
 0.0 0.0 0.0
 0.0 0.0 0.0
 0.0 0.0 0.0
 0.0 0.0 0.0
 0.0 0.0 0.0
 0.0 0.0 0.0
 0.0 0.0 0.0
 0.0 0.0 0.0
 0.0 0.0 0.0
 0.0 0.0 0.0
 0.0 0.0 0.0
 0.0 0.0 0.0
 0.0 0.0 0.0
 0.0 0.0 0.0
 0.0 0.0 0.0
 0.0 0.0 0.0
```

```
 0.0 0.0 0.0
 0.0 0.0 0.0
 0.0 0.0 0.0
 0.0 0.0 0.0
 0.0 0.0 0.0
 0.0 0.0 0.0
!entry.PC.unit.atoms table  str name  str type  int typex  int resx  int flags  int seq
int elmnt  dbl chg
 "C11" "cC" 0 1 131073 5 6 0.910403
 "O12" "oC" 0 1 131073 6 8 -0.671566
 "O11" "oS" 0 1 131073 7 8 -0.575489
 "C1" "cA" 0 1 131073 8 6 0.196939
 "HR" "hE" 0 1 131073 9 1 0.070469
 "HS" "hE" 0 1 131073 10 1 0.070469
 "C2" "cA" 0 1 131073 11 6 0.281242
 "HX" "hE" 0 1 131073 12 1 0.063253
 "C3" "cA" 0 1 131073 13 6 0.017459
 "HA" "hE" 0 1 131073 14 1 0.095591
 "HB" "hE" 0 1 131073 15 1 0.095591
 "O31" "oT" 0 1 131073 16 8 -0.509480
 "P31" "pA" 0 1 131073 17 15 1.339721
 "O32" "oT" 0 1 131073 18 8 -0.508176
 "C31" "cA" 0 1 131073 19 6 0.166801
 "H1A" "hE" 0 1 131073 20 1 0.078534
 "H1B" "hE" 0 1 131073 21 1 0.078534
 "C32" "cA" 0 1 131073 22 6 -0.170824
 "H2A" "hX" 0 1 131073 23 1 0.136415
 "H2B" "hX" 0 1 131073 24 1 0.136415
 "N31" "nA" 0 1 131073 25 7 0.245262
 "C33" "cA" 0 1 131073 26 6 -0.338973
 "H3A" "hX" 0 1 131073 27 1 0.172230
 "H3B" "hX" 0 1 131073 28 1 0.172230
 "H3C" "hX" 0 1 131073 29 1 0.172230
 "C34" "cA" 0 1 131073 30 6 -0.338973
 "H4A" "hX" 0 1 131073 31 1 0.172230
 "H4B" "hX" 0 1 131073 32 1 0.172230
 "H4C" "hX" 0 1 131073 33 1 0.172230
 "C35" "cA" 0 1 131073 34 6 -0.338973
 "H5A" "hX" 0 1 131073 35 1 0.172230
 "H5B" "hX" 0 1 131073 36 1 0.172230
 "H5C" "hX" 0 1 131073 37 1 0.172230
 "O33" "oP" 0 1 131073 38 8 -0.875812
 "O34" "oP" 0 1 131073 39 8 -0.875812
```

```
 "O21" "oS" 0 1 131073 40 8 -0.552948
 "C21" "cC" 0 1 131073 41 6 0.897613
 "O22" "oC" 0 1 131073 42 8 -0.673755
!entry.PC.unit.atomspertinfo table  str pname  str ptype  int ptypex  int pelmnt  dbl pchg
 "C11" "cC" 0 -1 0.0
 "O12" "oC" 0 -1 0.0
 "O11" "oS" 0 -1 0.0
 "C1" "cA" 0 -1 0.0
 "HR" "hE" 0 -1 0.0
 "HS" "hE" 0 -1 0.0
 "C2" "cA" 0 -1 0.0
 "HX" "hE" 0 -1 0.0
 "C3" "cA" 0 -1 0.0
 "HA" "hE" 0 -1 0.0
 "HB" "hE" 0 -1 0.0
 "O31" "oT" 0 -1 0.0
 "P31" "pA" 0 -1 0.0
 "O32" "oT" 0 -1 0.0
 "C31" "cA" 0 -1 0.0
 "H1A" "hE" 0 -1 0.0
 "H1B" "hE" 0 -1 0.0
 "C32" "cA" 0 -1 0.0
 "H2A" "hX" 0 -1 0.0
 "H2B" "hX" 0 -1 0.0
 "N31" "nA" 0 -1 0.0
 "C33" "cA" 0 -1 0.0
 "H3A" "hX" 0 -1 0.0
 "H3B" "hX" 0 -1 0.0
 "H3C" "hX" 0 -1 0.0
 "C34" "cA" 0 -1 0.0
 "H4A" "hX" 0 -1 0.0
 "H4B" "hX" 0 -1 0.0
 "H4C" "hX" 0 -1 0.0
 "C35" "cA" 0 -1 0.0
 "H5A" "hX" 0 -1 0.0
 "H5B" "hX" 0 -1 0.0
 "H5C" "hX" 0 -1 0.0
 "O33" "oP" 0 -1 0.0
 "O34" "oP" 0 -1 0.0
 "O21" "oS" 0 -1 0.0
 "C21" "cC" 0 -1 0.0
 "O22" "oC" 0 -1 0.0
!entry.PC.unit.boundbox array dbl
```

```
 -1.000000
 0.0
 0.0
 0.0
 0.0
!entry.PC.unit.childsequence single int
 2
!entry.PC.unit.connect array int
 1
 37
!entry.PC.unit.connectivity table  int atom1x  int atom2x  int flags
 1 2 2
 1 3 1
 3 4 1
 4 5 1
 4 6 1
 4 7 1
 7 8 1
 7 9 1
 7 36 1
 9 10 1
 9 11 1
 9 12 1
 12 13 1
 13 14 1
 13 34 1
 13 35 1
 14 15 1
 15 16 1
 15 17 1
 15 18 1
 18 19 1
 18 20 1
 18 21 1
 21 22 1
 21 26 1
 21 30 1
 22 23 1
 22 24 1
 22 25 1
 26 27 1
 26 28 1
 26 29 1
```

```
 30 31 1
 30 32 1
 30 33 1
 36 37 1
 37 38 2
!entry.PC.unit.hierarchy table  str abovetype  int abovex  str belowtype  int belowx
 "U" 0 "R" 1
 "R" 1 "A" 1
 "R" 1 "A" 2
 "R" 1 "A" 3
 "R" 1 "A" 4
 "R" 1 "A" 5
 "R" 1 "A" 6
 "R" 1 "A" 7
 "R" 1 "A" 8
 "R" 1 "A" 9
 "R" 1 "A" 10
 "R" 1 "A" 11
 "R" 1 "A" 12
 "R" 1 "A" 13
 "R" 1 "A" 14
 "R" 1 "A" 15
 "R" 1 "A" 16
 "R" 1 "A" 17
 "R" 1 "A" 18
 "R" 1 "A" 19
 "R" 1 "A" 20
 "R" 1 "A" 21
 "R" 1 "A" 22
 "R" 1 "A" 23
 "R" 1 "A" 24
 "R" 1 "A" 25
 "R" 1 "A" 26
 "R" 1 "A" 27
 "R" 1 "A" 28
 "R" 1 "A" 29
 "R" 1 "A" 30
 "R" 1 "A" 31
 "R" 1 "A" 32
 "R" 1 "A" 33
 "R" 1 "A" 34
 "R" 1 "A" 35
 "R" 1 "A" 36
```

```
 "R" 1 "A" 37
 "R" 1 "A" 38
!entry.PC.unit.name single str
 "PC"
!entry.PC.unit.positions table  dbl x  dbl y  dbl z
 4.694000 -2.109000 0.405000
 4.576000 -2.896000 -0.477000
 3.902000 -1.060000 0.566000
 2.854000 -0.874000 -0.377000
 2.157000 -1.696000 -0.314000
 3.266000 -0.836000 -1.375000
 2.165000 0.425000 -0.026000
 1.911000 0.438000 1.021000
 0.919000 0.667000 -0.863000
 1.151000 0.638000 -1.919000
 0.516000 1.643000 -0.623000
 -0.006000 -0.339000 -0.535000
 -1.430000 -0.403000 -1.277000
 -2.179000 0.795000 -0.393000
 -3.461000 1.133000 -0.789000
 -3.812000 0.531000 -1.617000
 -3.484000 2.165000 -1.127000
 -4.434000 1.074000 0.381000
 -5.396000 1.448000 0.054000
 -4.072000 1.701000 1.183000
 -4.708000 -0.280000 1.003000
 -5.092000 -1.293000 -0.033000
 -5.889000 -0.883000 -0.638000
 -5.442000 -2.177000 0.479000
 -4.222000 -1.537000 -0.624000
 -3.521000 -0.799000 1.770000
 -3.178000 -0.023000 2.437000
 -2.753000 -1.088000 1.075000
 -3.853000 -1.656000 2.338000
 -5.839000 -0.102000 1.952000
 -5.570000 0.638000 2.691000
 -6.044000 -1.043000 2.440000
 -6.716000 0.223000 1.411000
 -2.106000 -1.653000 -0.869000
 -1.344000 0.050000 -2.666000
 3.096000 1.472000 -0.298000
 3.110000 2.550000 0.475000
 2.361000 2.734000 1.379000
```

```
!entry.PC.unit.residueconnect table  int c1x  int c2x  int c3x  int c4x  int c5x  int c6x
 1 37 0 0 0 0
!entry.PC.unit.residues table  str name  int seq  int childseq  int startatomx  str restype  int imagingx
 "PC" 1 47 1 "?" 0
!entry.PC.unit.residuesPdbSequenceNumber array int
 0
!entry.PC.unit.solventcap array dbl
 -1.000000
 0.0
 0.0
 0.0
 0.0
!entry.PC.unit.velocities table  dbl x  dbl y  dbl z
 0.0 0.0 0.0
 0.0 0.0 0.0
 0.0 0.0 0.0
 0.0 0.0 0.0
 0.0 0.0 0.0
 0.0 0.0 0.0
 0.0 0.0 0.0
 0.0 0.0 0.0
 0.0 0.0 0.0
 0.0 0.0 0.0
 0.0 0.0 0.0
 0.0 0.0 0.0
 0.0 0.0 0.0
 0.0 0.0 0.0
 0.0 0.0 0.0
 0.0 0.0 0.0
 0.0 0.0 0.0
 0.0 0.0 0.0
 0.0 0.0 0.0
 0.0 0.0 0.0
 0.0 0.0 0.0
 0.0 0.0 0.0
 0.0 0.0 0.0
 0.0 0.0 0.0
 0.0 0.0 0.0
 0.0 0.0 0.0
 0.0 0.0 0.0
 0.0 0.0 0.0
 0.0 0.0 0.0
```

```
 0.0 0.0 0.0
 0.0 0.0 0.0
 0.0 0.0 0.0
 0.0 0.0 0.0
 0.0 0.0 0.0
 0.0 0.0 0.0
 0.0 0.0 0.0
 0.0 0.0 0.0
 0.0 0.0 0.0
!entry.PE.unit.atoms table  str name  str type  int typex  int resx  int flags  int seq
int elmnt  dbl chg
 "C11" "cC" 0 1 131073 5 6 0.888359
 "O12" "oC" 0 1 131073 6 8 -0.650802
 "O11" "oS" 0 1 131073 7 8 -0.593826
 "C1" "cA" 0 1 131073 8 6 0.273776
 "HR" "hE" 0 1 131073 9 1 0.044574
 "HS" "hE" 0 1 131073 10 1 0.044574
 "C2" "cA" 0 1 131073 11 6 0.339004
 "HX" "hE" 0 1 131073 12 1 0.032843
 "C3" "cA" 0 1 131073 13 6 0.040967
 "HA" "hE" 0 1 131073 14 1 0.090994
 "HB" "hE" 0 1 131073 15 1 0.090994
 "O31" "oT" 0 1 131073 16 8 -0.546623
 "P31" "pA" 0 1 131073 17 15 1.373656
 "O32" "oT" 0 1 131073 18 8 -0.514542
 "C31" "cA" 0 1 131073 19 6 0.075159
 "H1A" "hE" 0 1 131073 20 1 0.091516
 "H1B" "hE" 0 1 131073 21 1 0.091516
 "C32" "cA" 0 1 131073 22 6 0.077319
 "H2A" "hX" 0 1 131073 23 1 0.091630
 "H2B" "hX" 0 1 131073 24 1 0.091630
 "N31" "nA" 0 1 131073 25 7 -0.368132
 "HN1A" "hN" 0 1 131073 26 1 0.348549
 "HN1B" "hN" 0 1 131073 27 1 0.348549
 "HN1C" "hN" 0 1 131073 28 1 0.348549
 "O33" "oP" 0 1 131073 29 8 -0.877447
 "O34" "oP" 0 1 131073 30 8 -0.877447
 "O21" "oS" 0 1 131073 31 8 -0.575480
 "C21" "cC" 0 1 131073 32 6 0.869340
 "O22" "oC" 0 1 131073 33 8 -0.649199
!entry.PE.unit.atomspertinfo table  str pname  str ptype  int ptypex  int pelmnt  dbl pchg
 "C11" "cC" 0 -1 0.0
 "O12" "oC" 0 -1 0.0
```

```
 "O11" "oS" 0 -1 0.0
 "C1" "cA" 0 -1 0.0
 "HR" "hE" 0 -1 0.0
 "HS" "hE" 0 -1 0.0
 "C2" "cA" 0 -1 0.0
 "HX" "hE" 0 -1 0.0
 "C3" "cA" 0 -1 0.0
 "HA" "hE" 0 -1 0.0
 "HB" "hE" 0 -1 0.0
 "O31" "oT" 0 -1 0.0
 "P31" "pA" 0 -1 0.0
 "O32" "oT" 0 -1 0.0
 "C31" "cA" 0 -1 0.0
 "H1A" "hE" 0 -1 0.0
 "H1B" "hE" 0 -1 0.0
 "C32" "cA" 0 -1 0.0
 "H2A" "hX" 0 -1 0.0
 "H2B" "hX" 0 -1 0.0
 "N31" "nA" 0 -1 0.0
 "HN1A" "hN" 0 -1 0.0
 "HN1B" "hN" 0 -1 0.0
 "HN1C" "hN" 0 -1 0.0
 "O33" "oP" 0 -1 0.0
 "O34" "oP" 0 -1 0.0
 "O21" "oS" 0 -1 0.0
 "C21" "cC" 0 -1 0.0
 "O22" "oC" 0 -1 0.0
!entry.PE.unit.boundbox array dbl
 -1.000000
 0.0
 0.0
 0.0
 0.0
!entry.PE.unit.childsequence single int
 2
!entry.PE.unit.connect array int
 1
 28
!entry.PE.unit.connectivity table  int atom1x  int atom2x  int flags
 1 2 2
 1 3 1
 3 4 1
 4 5 1
```

```
 4 6 1
 4 7 1
 7 8 1
 7 9 1
 7 27 1
 9 10 1
 9 11 1
 9 12 1
 12 13 1
 13 14 1
 13 25 1
 13 26 1
 14 15 1
 15 16 1
 15 17 1
 15 18 1
 18 19 1
 18 20 1
 18 21 1
 21 22 1
 21 23 1
 21 24 1
 27 28 1
 28 29 2
!entry.PE.unit.hierarchy table  str abovetype  int abovex  str belowtype  int belowx
 "U" 0 "R" 1
 "R" 1 "A" 1
 "R" 1 "A" 2
 "R" 1 "A" 3
 "R" 1 "A" 4
 "R" 1 "A" 5
 "R" 1 "A" 6
 "R" 1 "A" 7
 "R" 1 "A" 8
 "R" 1 "A" 9
 "R" 1 "A" 10
 "R" 1 "A" 11
 "R" 1 "A" 12
 "R" 1 "A" 13
 "R" 1 "A" 14
 "R" 1 "A" 15
 "R" 1 "A" 16
 "R" 1 "A" 17
```

```
 "R" 1 "A" 18
 "R" 1 "A" 19
 "R" 1 "A" 20
 "R" 1 "A" 21
 "R" 1 "A" 22
 "R" 1 "A" 23
 "R" 1 "A" 24
 "R" 1 "A" 25
 "R" 1 "A" 26
 "R" 1 "A" 27
 "R" 1 "A" 28
 "R" 1 "A" 29
!entry.PE.unit.name single str
 "PE"
!entry.PE.unit.positions table  dbl x  dbl y  dbl z
 2.180000 2.916000 -0.064000
 1.200000 3.445000 0.353000
 2.300000 1.607000 -0.216000
 1.186000 0.802000 0.148000
 0.303000 1.147000 -0.363000
 1.024000 0.875000 1.214000
 1.505000 -0.620000 -0.256000
 1.807000 -0.650000 -1.291000
 0.330000 -1.562000 -0.031000
 0.041000 -1.559000 1.011000
 0.613000 -2.568000 -0.305000
 -0.732000 -1.142000 -0.860000
 -2.233000 -1.108000 -0.332000
 -2.136000 0.266000 0.585000
 -3.161000 0.573000 1.479000
 -2.877000 1.497000 1.967000
 -3.275000 -0.198000 2.230000
 -4.503000 0.755000 0.756000
 -4.980000 -0.196000 0.574000
 -5.171000 1.387000 1.325000
 -4.264000 1.356000 -0.588000
 -3.660000 2.160000 -0.543000
 -3.765000 0.574000 -1.162000
 -5.121000 1.620000 -1.043000
 -2.580000 -2.225000 0.542000
 -3.100000 -0.702000 -1.484000
 2.581000 -1.061000 0.567000
 3.515000 -1.854000 0.049000
```

```
 3.494000 -2.265000 -1.064000
!entry.PE.unit.residueconnect table  int c1x  int c2x  int c3x  int c4x  int c5x  int c6x
 1 28 0 0 0 0
!entry.PE.unit.residues table  str name  int seq  int childseq  int startatomx  str restype
int imagingx
 "PE" 1 38 1 "?" 0
!entry.PE.unit.residuesPdbSequenceNumber array int
 0
!entry.PE.unit.solventcap array dbl
 -1.000000
 0.0
 0.0
 0.0
 0.0
!entry.PE.unit.velocities table  dbl x  dbl y  dbl z
 0.0 0.0 0.0
 0.0 0.0 0.0
 0.0 0.0 0.0
 0.0 0.0 0.0
 0.0 0.0 0.0
 0.0 0.0 0.0
 0.0 0.0 0.0
 0.0 0.0 0.0
 0.0 0.0 0.0
 0.0 0.0 0.0
 0.0 0.0 0.0
 0.0 0.0 0.0
 0.0 0.0 0.0
 0.0 0.0 0.0
 0.0 0.0 0.0
 0.0 0.0 0.0
 0.0 0.0 0.0
 0.0 0.0 0.0
 0.0 0.0 0.0
 0.0 0.0 0.0
 0.0 0.0 0.0
 0.0 0.0 0.0
 0.0 0.0 0.0
 0.0 0.0 0.0
 0.0 0.0 0.0
 0.0 0.0 0.0
 0.0 0.0 0.0
 0.0 0.0 0.0
```

```
 0.0 0.0 0.0
!entry.PGR.unit.atoms table  str name  str type  int typex  int resx  int flags  int seq
int elmnt  dbl chg
 "C11" "cC" 0 1 131073 5 6 0.877006
 "O12" "oC" 0 1 131073 6 8 -0.649265
 "O11" "oS" 0 1 131073 7 8 -0.585165
 "C1" "cA" 0 1 131073 8 6 0.295119
 "HR" "hE" 0 1 131073 9 1 0.033797
 "HS" "hE" 0 1 131073 10 1 0.033797
 "C2" "cA" 0 1 131073 11 6 0.353807
 "HX" "hE" 0 1 131073 12 1 0.037488
 "C3" "cA" 0 1 131073 13 6 0.043713
 "HA" "hE" 0 1 131073 14 1 0.091041
 "HB" "hE" 0 1 131073 15 1 0.091041
 "O31" "oT" 0 1 131073 16 8 -0.546098
 "P31" "pA" 0 1 131073 17 15 1.380464
 "O32" "oT" 0 1 131073 18 8 -0.534308
 "C31" "cA" 0 1 131073 19 6 -0.056706
 "H1A" "hE" 0 1 131073 20 1 0.093354
 "H1B" "hE" 0 1 131073 21 1 0.093354
 "C32" "cA" 0 1 131073 22 6 0.336816
 "H2A" "hE" 0 1 131073 23 1 0.030042
 "O35" "oH" 0 1 131073 24 8 -0.726192
 "HO5A" "hO" 0 1 131073 25 1 0.439523
 "C33" "cA" 0 1 131073 26 6 0.193597
 "H3A" "hE" 0 1 131073 27 1 0.034715
 "H3B" "hE" 0 1 131073 28 1 0.034715
 "O33" "oP" 0 1 131073 29 8 -0.873444
 "O34" "oP" 0 1 131073 30 8 -0.873444
 "O21" "oS" 0 1 131073 31 8 -0.599131
 "C21" "cC" 0 1 131073 32 6 0.883669
 "O22" "oC" 0 1 131073 33 8 -0.655762
 "O36" "oH" 0 1 131073 38 8 -0.700871
 "HO6A" "hO" 0 1 131073 39 1 0.423328
!entry.PGR.unit.atomspertinfo table  str pname  str ptype  int ptypex  int pelmnt  dbl pchg
 "C11" "cC" 0 -1 0.0
 "O12" "oC" 0 -1 0.0
 "O11" "oS" 0 -1 0.0
 "C1" "cA" 0 -1 0.0
 "HR" "hE" 0 -1 0.0
 "HS" "hE" 0 -1 0.0
 "C2" "cA" 0 -1 0.0
 "HX" "hE" 0 -1 0.0
```

```
 "C3" "cA" 0 -1 0.0
 "HA" "hE" 0 -1 0.0
 "HB" "hE" 0 -1 0.0
 "O31" "oT" 0 -1 0.0
 "P31" "pA" 0 -1 0.0
 "O32" "oT" 0 -1 0.0
 "C31" "cA" 0 -1 0.0
 "H1A" "hE" 0 -1 0.0
 "H1B" "hE" 0 -1 0.0
 "C32" "cA" 0 -1 0.0
 "H2A" "hE" 0 -1 0.0
 "O35" "oH" 0 -1 0.0
 "HO5A" "hO" 0 -1 0.0
 "C33" "cA" 0 -1 0.0
 "H3A" "hE" 0 -1 0.0
 "H3B" "hE" 0 -1 0.0
 "O33" "oP" 0 -1 0.0
 "O34" "oP" 0 -1 0.0
 "O21" "oS" 0 -1 0.0
 "C21" "cC" 0 -1 0.0
 "O22" "oC" 0 -1 0.0
 "O36" "oH" 0 -1 0.0
 "HO6A" "hO" 0 -1 0.0
!entry.PGR.unit.boundbox array dbl
 -1.000000
 0.0
 0.0
 0.0
 0.0
!entry.PGR.unit.childsequence single int
 2
!entry.PGR.unit.connect array int
 1
 28
!entry.PGR.unit.connectivity table  int atom1x  int atom2x  int flags
 1 2 2
 1 3 1
 3 4 1
 4 5 1
 4 6 1
 4 7 1
 7 8 1
 7 9 1
```

```
 7 27 1
 9 10 1
 9 11 1
 9 12 1
 12 13 1
 13 14 1
 13 25 1
 13 26 1
 14 15 1
 15 16 1
 15 17 1
 15 18 1
 18 19 1
 18 20 1
 18 22 1
 20 21 1
 22 23 1
 22 24 1
 22 30 1
 27 28 1
 28 29 2
 30 31 1
!entry.PGR.unit.hierarchy table  str abovetype  int abovex  str belowtype  int belowx
 "U" 0 "R" 1
 "R" 1 "A" 1
 "R" 1 "A" 2
 "R" 1 "A" 3
 "R" 1 "A" 4
 "R" 1 "A" 5
 "R" 1 "A" 6
 "R" 1 "A" 7
 "R" 1 "A" 8
 "R" 1 "A" 9
 "R" 1 "A" 10
 "R" 1 "A" 11
 "R" 1 "A" 12
 "R" 1 "A" 13
 "R" 1 "A" 14
 "R" 1 "A" 15
 "R" 1 "A" 16
 "R" 1 "A" 17
 "R" 1 "A" 18
 "R" 1 "A" 19
```

```
 "R" 1 "A" 20
 "R" 1 "A" 21
 "R" 1 "A" 22
 "R" 1 "A" 23
 "R" 1 "A" 24
 "R" 1 "A" 25
 "R" 1 "A" 26
 "R" 1 "A" 27
 "R" 1 "A" 28
 "R" 1 "A" 29
 "R" 1 "A" 30
 "R" 1 "A" 31
!entry.PGR.unit.name single str
 "PGR"
!entry.PGR.unit.positions table  dbl x  dbl y  dbl z
 -4.477000 -1.887000 -0.726000
 -4.371000 -2.868000 -0.063000
 -3.639000 -0.870000 -0.694000
 -2.503000 -0.948000 0.169000
 -1.828000 -1.711000 -0.185000
 -2.829000 -1.195000 1.169000
 -1.828000 0.404000 0.138000
 -1.630000 0.695000 -0.880000
 -0.548000 0.426000 0.959000
 -0.757000 0.182000 1.993000
 -0.133000 1.429000 0.931000
 0.324000 -0.498000 0.392000
 1.792000 -0.746000 1.103000
 2.555000 0.619000 0.624000
 2.986000 0.770000 -0.700000
 2.623000 1.721000 -1.076000
 2.604000 -0.014000 -1.340000
 4.505000 0.786000 -0.761000
 4.873000 1.541000 -0.068000
 4.862000 1.134000 -2.080000
 5.785000 0.936000 -2.177000
 5.129000 -0.545000 -0.390000
 4.915000 -0.793000 0.640000
 4.728000 -1.331000 -1.018000
 2.390000 -1.892000 0.403000
 1.622000 -0.640000 2.557000
 -2.727000 1.350000 0.732000
 -3.217000 2.354000 0.028000
```

```
 -2.963000 2.587000 -1.110000
 6.529000 -0.405000 -0.601000
 6.945000 -1.239000 -0.440000
!entry.PGR.unit.residueconnect table  int c1x  int c2x  int c3x  int c4x  int c5x  int c6x
 1 28 0 0 0 0
!entry.PGR.unit.residues table  str name  int seq  int childseq  int startatomx  str
restype  int imagingx
 "PGR" 1 40 1 "?" 0
!entry.PGR.unit.residuesPdbSequenceNumber array int
 0
!entry.PGR.unit.solventcap array dbl
 -1.000000
 0.0
 0.0
 0.0
 0.0
!entry.PGR.unit.velocities table  dbl x  dbl y  dbl z
 0.0 0.0 0.0
 0.0 0.0 0.0
 0.0 0.0 0.0
 0.0 0.0 0.0
 0.0 0.0 0.0
 0.0 0.0 0.0
 0.0 0.0 0.0
 0.0 0.0 0.0
 0.0 0.0 0.0
 0.0 0.0 0.0
 0.0 0.0 0.0
 0.0 0.0 0.0
 0.0 0.0 0.0
 0.0 0.0 0.0
 0.0 0.0 0.0
 0.0 0.0 0.0
 0.0 0.0 0.0
 0.0 0.0 0.0
 0.0 0.0 0.0
 0.0 0.0 0.0
 0.0 0.0 0.0
 0.0 0.0 0.0
 0.0 0.0 0.0
 0.0 0.0 0.0
 0.0 0.0 0.0
 0.0 0.0 0.0
```

```
 0.0 0.0 0.0
 0.0 0.0 0.0
 0.0 0.0 0.0
 0.0 0.0 0.0
 0.0 0.0 0.0
!entry.PGS.unit.atoms table  str name  str type  int typex  int resx  int flags  int seq
int elmnt  dbl chg
 "C11" "cC" 0 1 131073 5 6 0.877006
 "O12" "oC" 0 1 131073 6 8 -0.649265
 "O11" "oS" 0 1 131073 7 8 -0.585165
 "C1" "cA" 0 1 131073 8 6 0.295119
 "HR" "hE" 0 1 131073 9 1 0.033797
 "HS" "hE" 0 1 131073 10 1 0.033797
 "C2" "cA" 0 1 131073 11 6 0.353807
 "HX" "hE" 0 1 131073 12 1 0.037488
 "C3" "cA" 0 1 131073 13 6 0.043713
 "HA" "hE" 0 1 131073 14 1 0.091041
 "HB" "hE" 0 1 131073 15 1 0.091041
 "O31" "oT" 0 1 131073 16 8 -0.546098
 "P31" "pA" 0 1 131073 17 15 1.380464
 "O32" "oT" 0 1 131073 18 8 -0.534308
 "C31" "cA" 0 1 131073 19 6 -0.056706
 "H1A" "hE" 0 1 131073 20 1 0.093354
 "H1B" "hE" 0 1 131073 21 1 0.093354
 "C32" "cA" 0 1 131073 22 6 0.336816
 "H2A" "hE" 0 1 131073 23 1 0.030042
 "O35" "oH" 0 1 131073 24 8 -0.726192
 "HO5A" "hO" 0 1 131073 25 1 0.439523
 "C33" "cA" 0 1 131073 26 6 0.193597
 "H3A" "hE" 0 1 131073 27 1 0.034715
 "H3B" "hE" 0 1 131073 28 1 0.034715
 "O33" "oP" 0 1 131073 29 8 -0.873444
 "O34" "oP" 0 1 131073 30 8 -0.873444
 "O21" "oS" 0 1 131073 31 8 -0.599131
 "C21" "cC" 0 1 131073 32 6 0.883669
 "O22" "oC" 0 1 131073 33 8 -0.655762
 "O36" "oH" 0 1 131073 38 8 -0.700871
 "HO6A" "hO" 0 1 131073 39 1 0.423328
!entry.PGS.unit.atomspertinfo table  str pname  str ptype  int ptypex  int pelmnt  dbl pchg
 "C11" "cC" 0 -1 0.0
 "O12" "oC" 0 -1 0.0
 "O11" "oS" 0 -1 0.0
 "C1" "cA" 0 -1 0.0
```

```
 "HR" "hE" 0 -1 0.0
 "HS" "hE" 0 -1 0.0
 "C2" "cA" 0 -1 0.0
 "HX" "hE" 0 -1 0.0
 "C3" "cA" 0 -1 0.0
 "HA" "hE" 0 -1 0.0
 "HB" "hE" 0 -1 0.0
 "O31" "oT" 0 -1 0.0
 "P31" "pA" 0 -1 0.0
 "O32" "oT" 0 -1 0.0
 "C31" "cA" 0 -1 0.0
 "H1A" "hE" 0 -1 0.0
 "H1B" "hE" 0 -1 0.0
 "C32" "cA" 0 -1 0.0
 "H2A" "hE" 0 -1 0.0
 "O35" "oH" 0 -1 0.0
 "HO5A" "hO" 0 -1 0.0
 "C33" "cA" 0 -1 0.0
 "H3A" "hE" 0 -1 0.0
 "H3B" "hE" 0 -1 0.0
 "O33" "oP" 0 -1 0.0
 "O34" "oP" 0 -1 0.0
 "O21" "oS" 0 -1 0.0
 "C21" "cC" 0 -1 0.0
 "O22" "oC" 0 -1 0.0
 "O36" "oH" 0 -1 0.0
 "HO6A" "hO" 0 -1 0.0
!entry.PGS.unit.boundbox array dbl
 -1.000000
 0.0
 0.0
 0.0
 0.0
!entry.PGS.unit.childsequence single int
 2
!entry.PGS.unit.connect array int
 1
 28
!entry.PGS.unit.connectivity table  int atom1x  int atom2x  int flags
 1 2 2
 1 3 1
 3 4 1
 4 5 1
```

```
 4 6 1
 4 7 1
 7 8 1
 7 9 1
 7 27 1
 9 10 1
 9 11 1
 9 12 1
 12 13 1
 13 14 1
 13 25 1
 13 26 1
 14 15 1
 15 16 1
 15 17 1
 15 18 1
 18 19 1
 18 20 1
 18 22 1
 20 21 1
 22 23 1
 22 24 1
 22 30 1
 27 28 1
 28 29 2
 30 31 1
!entry.PGS.unit.hierarchy table  str abovetype  int abovex  str belowtype  int belowx
 "U" 0 "R" 1
 "R" 1 "A" 1
 "R" 1 "A" 2
 "R" 1 "A" 3
 "R" 1 "A" 4
 "R" 1 "A" 5
 "R" 1 "A" 6
 "R" 1 "A" 7
 "R" 1 "A" 8
 "R" 1 "A" 9
 "R" 1 "A" 10
 "R" 1 "A" 11
 "R" 1 "A" 12
 "R" 1 "A" 13
 "R" 1 "A" 14
 "R" 1 "A" 15
```

```
 "R" 1 "A" 16
 "R" 1 "A" 17
 "R" 1 "A" 18
 "R" 1 "A" 19
 "R" 1 "A" 20
 "R" 1 "A" 21
 "R" 1 "A" 22
 "R" 1 "A" 23
 "R" 1 "A" 24
 "R" 1 "A" 25
 "R" 1 "A" 26
 "R" 1 "A" 27
 "R" 1 "A" 28
 "R" 1 "A" 29
 "R" 1 "A" 30
 "R" 1 "A" 31
!entry.PGS.unit.name single str
 "PGS"
!entry.PGS.unit.positions table  dbl x  dbl y  dbl z
 -4.694000 -1.417000 0.240000
 -4.509000 -2.326000 0.983000
 -3.752000 -0.596000 -0.184000
 -2.409000 -0.821000 0.246000
 -2.038000 -1.739000 -0.181000
 -2.383000 -0.893000 1.323000
 -1.588000 0.353000 -0.240000
 -1.724000 0.488000 -1.299000
 -0.111000 0.199000 0.091000
 0.027000 0.102000 1.161000
 0.417000 1.085000 -0.244000
 0.348000 -0.934000 -0.576000
 1.837000 -1.519000 -0.273000
 2.681000 -0.306000 -0.969000
 4.081000 -0.360000 -0.920000
 4.460000 -0.364000 -1.935000
 4.422000 -1.266000 -0.434000
 4.680000 0.829000 -0.187000
 5.745000 0.618000 -0.068000
 4.513000 1.978000 -0.993000
 4.845000 2.731000 -0.522000
 4.080000 1.063000 1.197000
 4.575000 1.928000 1.641000
 3.031000 1.312000 1.083000
```

```
 2.005000 -2.765000 -1.022000
 2.084000 -1.434000 1.191000
 -2.046000 1.522000 0.450000
 -2.640000 2.506000 -0.204000
 -2.822000 2.540000 -1.377000
 4.267000 -0.017000 2.049000
 3.542000 -0.633000 1.892000
!entry.PGS.unit.residueconnect table  int c1x  int c2x  int c3x  int c4x  int c5x  int c6x
 0 0 0 0 0 0
!entry.PGS.unit.residues table  str name  int seq  int childseq  int startatomx  str
restype  int imagingx
 "PGS" 1 40 1 "?" 0
!entry.PGS.unit.residuesPdbSequenceNumber array int
 0
!entry.PGS.unit.solventcap array dbl
 -1.000000
 0.0
 0.0
 0.0
 0.0
!entry.PGS.unit.velocities table  dbl x  dbl y  dbl z
 0.0 0.0 0.0
 0.0 0.0 0.0
 0.0 0.0 0.0
 0.0 0.0 0.0
 0.0 0.0 0.0
 0.0 0.0 0.0
 0.0 0.0 0.0
 0.0 0.0 0.0
 0.0 0.0 0.0
 0.0 0.0 0.0
 0.0 0.0 0.0
 0.0 0.0 0.0
 0.0 0.0 0.0
 0.0 0.0 0.0
 0.0 0.0 0.0
 0.0 0.0 0.0
 0.0 0.0 0.0
 0.0 0.0 0.0
 0.0 0.0 0.0
 0.0 0.0 0.0
 0.0 0.0 0.0
 0.0 0.0 0.0
```

```
 0.0 0.0 0.0
 0.0 0.0 0.0
 0.0 0.0 0.0
 0.0 0.0 0.0
 0.0 0.0 0.0
 0.0 0.0 0.0
 0.0 0.0 0.0
 0.0 0.0 0.0
 0.0 0.0 0.0
!entry.PH-.unit.atoms table  str name  str type  int typex  int resx  int flags  int seq
int elmnt  dbl chg
 "C11" "cC" 0 1 131073 5 6 0.888207
 "O12" "oC" 0 1 131073 6 8 -0.671142
 "O11" "oS" 0 1 131073 7 8 -0.547824
 "C1" "cA" 0 1 131073 8 6 0.185299
 "HR" "hE" 0 1 131073 9 1 0.067676
 "HS" "hE" 0 1 131073 10 1 0.067676
 "C2" "cA" 0 1 131073 11 6 0.283546
 "HX" "hE" 0 1 131073 12 1 0.063862
 "C3" "cA" 0 1 131073 13 6 0.081528
 "HA" "hE" 0 1 131073 14 1 0.083099
 "HB" "hE" 0 1 131073 15 1 0.083099
 "O31" "oT" 0 1 131073 16 8 -0.594197
 "P31" "pA" 0 1 131073 17 15 1.456663
 "O32" "oH" 0 1 131073 18 8 -0.706197
 "HO2A" "hO" 0 1 131073 19 1 0.415628
 "O33" "oP" 0 1 131073 20 8 -0.914158
 "O34" "oP" 0 1 131073 21 8 -0.914158
 "O21" "oS" 0 1 131073 22 8 -0.554487
 "C21" "cC" 0 1 131073 23 6 0.897502
 "O22" "oC" 0 1 131073 24 8 -0.671622
!entry.PH-.unit.atomspertinfo table  str pname  str ptype  int ptypex  int pelmnt  dbl pchg
 "C11" "cC" 0 -1 0.0
 "O12" "oC" 0 -1 0.0
 "O11" "oS" 0 -1 0.0
 "C1" "cA" 0 -1 0.0
 "HR" "hE" 0 -1 0.0
 "HS" "hE" 0 -1 0.0
 "C2" "cA" 0 -1 0.0
 "HX" "hE" 0 -1 0.0
 "C3" "cA" 0 -1 0.0
 "HA" "hE" 0 -1 0.0
 "HB" "hE" 0 -1 0.0
```

```
 "O31" "oT" 0 -1 0.0
 "P31" "pA" 0 -1 0.0
 "O32" "oH" 0 -1 0.0
 "HO2A" "hO" 0 -1 0.0
 "O33" "oP" 0 -1 0.0
 "O34" "oP" 0 -1 0.0
 "O21" "oS" 0 -1 0.0
 "C21" "cC" 0 -1 0.0
 "O22" "oC" 0 -1 0.0
!entry.PH-.unit.boundbox array dbl
 -1.000000
 0.0
 0.0
 0.0
 0.0
!entry.PH-.unit.childsequence single int
 2
!entry.PH-.unit.connect array int
 1
 19
!entry.PH-.unit.connectivity table  int atom1x  int atom2x  int flags
 1 2 2
 1 3 1
 3 4 1
 4 5 1
 4 6 1
 4 7 1
 7 8 1
 7 9 1
 7 18 1
 9 10 1
 9 11 1
 9 12 1
 12 13 1
 13 14 1
 13 16 1
 13 17 1
 14 15 1
 18 19 1
 19 20 2
!entry.PH-.unit.hierarchy table  str abovetype  int abovex  str belowtype  int belowx
 "U" 0 "R" 1
 "R" 1 "A" 1
```

```
 "R" 1 "A" 2
 "R" 1 "A" 3
 "R" 1 "A" 4
 "R" 1 "A" 5
 "R" 1 "A" 6
 "R" 1 "A" 7
 "R" 1 "A" 8
 "R" 1 "A" 9
 "R" 1 "A" 10
 "R" 1 "A" 11
 "R" 1 "A" 12
 "R" 1 "A" 13
 "R" 1 "A" 14
 "R" 1 "A" 15
 "R" 1 "A" 16
 "R" 1 "A" 17
 "R" 1 "A" 18
 "R" 1 "A" 19
 "R" 1 "A" 20
!entry.PH-.unit.name single str
 "PH-"
!entry.PH-.unit.positions table  dbl x  dbl y  dbl z
 -3.044000 -2.013000 0.046000
 -2.728000 -2.874000 0.802000
 -2.283000 -0.988000 -0.281000
 -0.971000 -0.903000 0.280000
 -0.353000 -1.692000 -0.118000
 -1.034000 -1.005000 1.353000
 -0.409000 0.446000 -0.105000
 -0.481000 0.586000 -1.171000
 1.029000 0.623000 0.360000
 1.091000 0.533000 1.438000
 1.360000 1.622000 0.090000
 1.789000 -0.352000 -0.272000
 3.414000 -0.414000 0.038000
 3.909000 0.868000 -0.850000
 4.131000 0.544000 -1.712000
 3.901000 -1.629000 -0.630000
 3.601000 -0.081000 1.456000
 -1.197000 1.447000 0.555000
 -1.896000 2.327000 -0.137000
 -1.933000 2.399000 -1.324000
!entry.PH-.unit.residueconnect table  int c1x  int c2x  int c3x  int c4x  int c5x  int c6x
```

```
 1 19 0 0 0 0
!entry.PH-.unit.residues table  str name  int seq  int childseq  int startatomx  str
restype  int imagingx
 "PH-" 1 29 1 "?" 0
!entry.PH-.unit.residuesPdbSequenceNumber array int
 0
!entry.PH-.unit.solventcap array dbl
 -1.000000
 0.0
 0.0
 0.0
 0.0
!entry.PH-.unit.velocities table  dbl x  dbl y  dbl z
 0.0 0.0 0.0
 0.0 0.0 0.0
 0.0 0.0 0.0
 0.0 0.0 0.0
 0.0 0.0 0.0
 0.0 0.0 0.0
 0.0 0.0 0.0
 0.0 0.0 0.0
 0.0 0.0 0.0
 0.0 0.0 0.0
 0.0 0.0 0.0
 0.0 0.0 0.0
 0.0 0.0 0.0
 0.0 0.0 0.0
 0.0 0.0 0.0
 0.0 0.0 0.0
 0.0 0.0 0.0
 0.0 0.0 0.0
 0.0 0.0 0.0
 0.0 0.0 0.0
!entry.PS.unit.atoms table  str name  str type  int typex  int resx  int flags  int seq
int elmnt  dbl chg
 "C11" "cC" 0 1 131073 5 6 0.887549
 "O12" "oC" 0 1 131073 6 8 -0.663331
 "O11" "oS" 0 1 131073 7 8 -0.579073
 "C1" "cA" 0 1 131073 8 6 0.271173
 "HR" "hE" 0 1 131073 9 1 0.037454
 "HS" "hE" 0 1 131073 10 1 0.037454
 "C2" "cA" 0 1 131073 11 6 0.440650
 "HX" "hE" 0 1 131073 12 1 0.002473
```

```
 "C3" "cA" 0 1 131073 13 6 0.012366
 "HA" "hE" 0 1 131073 14 1 0.097347
 "HB" "hE" 0 1 131073 15 1 0.097347
 "O31" "oT" 0 1 131073 16 8 -0.556992
 "P31" "pA" 0 1 131073 17 15 1.380208
 "O32" "oT" 0 1 131073 18 8 -0.520128
 "C31" "cA" 0 1 131073 19 6 0.060375
 "H1A" "hE" 0 1 131073 20 1 0.093484
 "H1B" "hE" 0 1 131073 21 1 0.093484
 "C32" "cA" 0 1 131073 22 6 0.039448
 "H2A" "hX" 0 1 131073 23 1 0.090549
 "N31" "nA" 0 1 131073 24 7 -0.414327
 "HN1A" "hN" 0 1 131073 25 1 0.353866
 "HN1B" "hN" 0 1 131073 26 1 0.353866
 "HN1C" "hN" 0 1 131073 27 1 0.353866
 "C33" "cC" 0 1 131073 28 6 0.809867
 "O33" "oP" 0 1 131073 29 8 -0.877922
 "O34" "oP" 0 1 131073 30 8 -0.877922
 "O21" "oS" 0 1 131073 31 8 -0.634381
 "C21" "cC" 0 1 131073 32 6 0.891897
 "O22" "oC" 0 1 131073 33 8 -0.650291
 "O35" "oO" 0 1 131073 38 8 -0.815178
 "O36" "oO" 0 1 131073 39 8 -0.815178
!entry.PS.unit.atomspertinfo table  str pname  str ptype  int ptypex  int pelmnt  dbl pchg
 "C11" "cC" 0 -1 0.0
 "O12" "oC" 0 -1 0.0
 "O11" "oS" 0 -1 0.0
 "C1" "cA" 0 -1 0.0
 "HR" "hE" 0 -1 0.0
 "HS" "hE" 0 -1 0.0
 "C2" "cA" 0 -1 0.0
 "HX" "hE" 0 -1 0.0
 "C3" "cA" 0 -1 0.0
 "HA" "hE" 0 -1 0.0
 "HB" "hE" 0 -1 0.0
 "O31" "oT" 0 -1 0.0
 "P31" "pA" 0 -1 0.0
 "O32" "oT" 0 -1 0.0
 "C31" "cA" 0 -1 0.0
 "H1A" "hE" 0 -1 0.0
 "H1B" "hE" 0 -1 0.0
 "C32" "cA" 0 -1 0.0
 "H2A" "hX" 0 -1 0.0
```

```
 "N31" "nA" 0 -1 0.0
 "HN1A" "hN" 0 -1 0.0
 "HN1B" "hN" 0 -1 0.0
 "HN1C" "hN" 0 -1 0.0
 "C33" "cC" 0 -1 0.0
 "O33" "oP" 0 -1 0.0
 "O34" "oP" 0 -1 0.0
 "O21" "oS" 0 -1 0.0
 "C21" "cC" 0 -1 0.0
 "O22" "oC" 0 -1 0.0
 "O35" "oO" 0 -1 0.0
 "O36" "oO" 0 -1 0.0
!entry.PS.unit.boundbox array dbl
 -1.000000
 0.0
 0.0
 0.0
 0.0
!entry.PS.unit.childsequence single int
 2
!entry.PS.unit.connect array int
 1
 28
!entry.PS.unit.connectivity table  int atom1x  int atom2x  int flags
 1 2 2
 1 3 1
 3 4 1
 4 5 1
 4 6 1
 4 7 1
 7 8 1
 7 9 1
 7 27 1
 9 10 1
 9 11 1
 9 12 1
 12 13 1
 13 14 1
 13 25 1
 13 26 1
 14 15 1
 15 16 1
 15 17 1
```

```
 15 18 1
 18 19 1
 18 20 1
 18 24 1
 20 21 1
 20 22 1
 20 23 1
 24 30 1
 24 31 1
 27 28 1
 28 29 2
!entry.PS.unit.hierarchy table  str abovetype  int abovex  str belowtype  int belowx
 "U" 0 "R" 1
 "R" 1 "A" 1
 "R" 1 "A" 2
 "R" 1 "A" 3
 "R" 1 "A" 4
 "R" 1 "A" 5
 "R" 1 "A" 6
 "R" 1 "A" 7
 "R" 1 "A" 8
 "R" 1 "A" 9
 "R" 1 "A" 10
 "R" 1 "A" 11
 "R" 1 "A" 12
 "R" 1 "A" 13
 "R" 1 "A" 14
 "R" 1 "A" 15
 "R" 1 "A" 16
 "R" 1 "A" 17
 "R" 1 "A" 18
 "R" 1 "A" 19
 "R" 1 "A" 20
 "R" 1 "A" 21
 "R" 1 "A" 22
 "R" 1 "A" 23
 "R" 1 "A" 24
 "R" 1 "A" 25
 "R" 1 "A" 26
 "R" 1 "A" 27
 "R" 1 "A" 28
 "R" 1 "A" 29
 "R" 1 "A" 30
```

"R" 1 "A" 31
!entry.PS.unit.name single str
"PS"
!entry.PS.unit.positions table dbl x dbl y dbl z
-4.858000 -1.850000 -0.410000
-4.698000 -2.763000 0.333000
-4.019000 -0.838000 -0.535000
-2.831000 -0.844000 0.255000
-2.200000 -1.666000 -0.042000
-3.092000 -0.954000 1.297000
-2.131000 0.474000 0.008000
-1.992000 0.627000 -1.049000
-0.799000 0.566000 0.736000
-0.935000 0.434000 1.801000
-0.371000 1.548000 0.565000
0.031000 -0.431000 0.214000
1.459000 -0.736000 0.918000
2.273000 0.544000 0.314000
3.667000 0.633000 0.494000
3.999000 -0.001000 1.309000
3.918000 1.652000 0.742000
4.399000 0.283000 -0.793000
4.021000 0.911000 -1.586000
4.144000 -1.146000 -1.167000
5.037000 -1.599000 -0.965000
3.345000 -1.571000 -0.652000
3.974000 -1.245000 -2.151000
5.947000 0.464000 -0.664000
2.019000 -1.944000 0.253000
1.341000 -0.631000 2.375000
-2.961000 1.514000 0.532000
-3.475000 2.435000 -0.269000
-3.284000 2.507000 -1.439000
6.589000 -0.587000 -0.792000
6.300000 1.609000 -0.448000
!entry.PS.unit.residueconnect table int c1x int c2x int c3x int c4x int c5x int c6x
1 28 0 0 0 0
!entry.PS.unit.residues table str name int seq int childseq int startatomx str restype int imagingx
"PS" 1 40 1 "?" 0
!entry.PS.unit.residuesPdbSequenceNumber array int
0
!entry.PS.unit.solventcap array dbl

```
 -1.000000
 0.0
 0.0
 0.0
 0.0
!entry.PS.unit.velocities table  dbl x  dbl y  dbl z
 0.0 0.0 0.0
 0.0 0.0 0.0
 0.0 0.0 0.0
 0.0 0.0 0.0
 0.0 0.0 0.0
 0.0 0.0 0.0
 0.0 0.0 0.0
 0.0 0.0 0.0
 0.0 0.0 0.0
 0.0 0.0 0.0
 0.0 0.0 0.0
 0.0 0.0 0.0
 0.0 0.0 0.0
 0.0 0.0 0.0
 0.0 0.0 0.0
 0.0 0.0 0.0
 0.0 0.0 0.0
 0.0 0.0 0.0
 0.0 0.0 0.0
 0.0 0.0 0.0
 0.0 0.0 0.0
 0.0 0.0 0.0
 0.0 0.0 0.0
 0.0 0.0 0.0
 0.0 0.0 0.0
 0.0 0.0 0.0
 0.0 0.0 0.0
 0.0 0.0 0.0
 0.0 0.0 0.0
 0.0 0.0 0.0
 0.0 0.0 0.0
!entry.SA.unit.atoms table  str name  str type  int typex  int resx  int flags  int seq
int elmnt  dbl chg
 "C116" "cD" 0 1 131073 1 6 -0.199999
 "H16R" "hL" 0 1 131073 2 1 0.049471
 "H16S" "hL" 0 1 131073 3 1 0.049471
 "H16T" "hL" 0 1 131073 4 1 0.049471
```

```
 "C115" "cD" 0 1 131073 5 6 -0.046781
 "H15R" "hL" 0 1 131073 6 1 0.026484
 "H15S" "hL" 0 1 131073 7 1 0.026484
 "C114" "cD" 0 1 131073 8 6 0.044648
 "H14R" "hL" 0 1 131073 9 1 0.010970
 "H14S" "hL" 0 1 131073 10 1 0.010970
 "C113" "cD" 0 1 131073 11 6 -0.119225
 "H13R" "hL" 0 1 131073 12 1 0.040330
 "H13S" "hL" 0 1 131073 13 1 0.040330
 "C112" "cD" 0 1 131073 14 6 0.001048
 "H12R" "hL" 0 1 131073 15 1 0.016935
 "H12S" "hL" 0 1 131073 16 1 0.016935
 "C111" "cD" 0 1 131073 17 6 -0.079417
 "H11R" "hL" 0 1 131073 18 1 0.023502
 "H11S" "hL" 0 1 131073 19 1 0.023502
 "C110" "cD" 0 1 131073 20 6 -0.066372
 "H10R" "hL" 0 1 131073 21 1 0.021723
 "H10S" "hL" 0 1 131073 22 1 0.021723
 "C19" "cD" 0 1 131073 23 6 0.053352
 "H9R" "hL" 0 1 131073 24 1 0.020200
 "H9S" "hL" 0 1 131073 25 1 0.020200
 "C18" "cD" 0 1 131073 26 6 -0.187889
 "H8R" "hL" 0 1 131073 27 1 0.046909
 "H8S" "hL" 0 1 131073 28 1 0.046909
 "C17" "cD" 0 1 131073 29 6 0.049288
 "H7R" "hL" 0 1 131073 30 1 0.006045
 "H7S" "hL" 0 1 131073 31 1 0.006045
 "C16" "cD" 0 1 131073 32 6 -0.012997
 "H6R" "hL" 0 1 131073 33 1 0.003223
 "H6S" "hL" 0 1 131073 34 1 0.003223
 "C15" "cD" 0 1 131073 35 6 0.093171
 "H5R" "hL" 0 1 131073 36 1 -0.011690
 "H5S" "hL" 0 1 131073 37 1 -0.011690
 "C14" "cD" 0 1 131073 38 6 -0.258138
 "H4R" "hL" 0 1 131073 39 1 0.076843
 "H4S" "hL" 0 1 131073 40 1 0.076843
 "C13" "cB" 0 1 131073 41 6 0.028290
 "H3R" "hB" 0 1 131073 42 1 0.116281
 "C12" "cB" 0 1 131073 43 6 -0.341889
 "H2R" "hB" 0 1 131073 44 1 0.145550
!entry.SA.unit.atomspertinfo table  str pname  str ptype  int ptypex  int pelmnt  dbl pchg
 "C116" "cD" 0 -1 0.0
 "H16R" "hL" 0 -1 0.0
```

```
 "H16S" "hL" 0 -1 0.0
 "H16T" "hL" 0 -1 0.0
 "C115" "cD" 0 -1 0.0
 "H15R" "hL" 0 -1 0.0
 "H15S" "hL" 0 -1 0.0
 "C114" "cD" 0 -1 0.0
 "H14R" "hL" 0 -1 0.0
 "H14S" "hL" 0 -1 0.0
 "C113" "cD" 0 -1 0.0
 "H13R" "hL" 0 -1 0.0
 "H13S" "hL" 0 -1 0.0
 "C112" "cD" 0 -1 0.0
 "H12R" "hL" 0 -1 0.0
 "H12S" "hL" 0 -1 0.0
 "C111" "cD" 0 -1 0.0
 "H11R" "hL" 0 -1 0.0
 "H11S" "hL" 0 -1 0.0
 "C110" "cD" 0 -1 0.0
 "H10R" "hL" 0 -1 0.0
 "H10S" "hL" 0 -1 0.0
 "C19" "cD" 0 -1 0.0
 "H9R" "hL" 0 -1 0.0
 "H9S" "hL" 0 -1 0.0
 "C18" "cD" 0 -1 0.0
 "H8R" "hL" 0 -1 0.0
 "H8S" "hL" 0 -1 0.0
 "C17" "cD" 0 -1 0.0
 "H7R" "hL" 0 -1 0.0
 "H7S" "hL" 0 -1 0.0
 "C16" "cD" 0 -1 0.0
 "H6R" "hL" 0 -1 0.0
 "H6S" "hL" 0 -1 0.0
 "C15" "cD" 0 -1 0.0
 "H5R" "hL" 0 -1 0.0
 "H5S" "hL" 0 -1 0.0
 "C14" "cD" 0 -1 0.0
 "H4R" "hL" 0 -1 0.0
 "H4S" "hL" 0 -1 0.0
 "C13" "cB" 0 -1 0.0
 "H3R" "hB" 0 -1 0.0
 "C12" "cB" 0 -1 0.0
 "H2R" "hB" 0 -1 0.0
!entry.SA.unit.boundbox array dbl
```

```
 -1.000000
 0.0
 0.0
 0.0
 0.0
!entry.SA.unit.childsequence single int
 2
!entry.SA.unit.connect array int
 43
 43
!entry.SA.unit.connectivity table  int atom1x  int atom2x  int flags
 1 5 1
 1 3 1
 1 2 1
 1 4 1
 5 8 1
 5 7 1
 5 6 1
 8 11 1
 8 9 1
 8 10 1
 11 14 1
 11 13 1
 11 12 1
 14 17 1
 14 16 1
 14 15 1
 17 20 1
 17 19 1
 17 18 1
 20 23 1
 20 21 1
 20 22 1
 23 26 1
 23 24 1
 23 25 1
 26 29 1
 26 28 1
 26 27 1
 29 32 1
 29 31 1
 29 30 1
 32 35 1
```

```
 32 33 1
 32 34 1
 35 38 1
 35 37 1
 35 36 1
 38 41 1
 38 39 1
 38 40 1
 41 43 1
 41 42 1
 43 44 1
!entry.SA.unit.hierarchy table  str abovetype  int abovex  str belowtype  int belowx
 "U" 0 "R" 1
 "R" 1 "A" 1
 "R" 1 "A" 2
 "R" 1 "A" 3
 "R" 1 "A" 4
 "R" 1 "A" 5
 "R" 1 "A" 6
 "R" 1 "A" 7
 "R" 1 "A" 8
 "R" 1 "A" 9
 "R" 1 "A" 10
 "R" 1 "A" 11
 "R" 1 "A" 12
 "R" 1 "A" 13
 "R" 1 "A" 14
 "R" 1 "A" 15
 "R" 1 "A" 16
 "R" 1 "A" 17
 "R" 1 "A" 18
 "R" 1 "A" 19
 "R" 1 "A" 20
 "R" 1 "A" 21
 "R" 1 "A" 22
 "R" 1 "A" 23
 "R" 1 "A" 24
 "R" 1 "A" 25
 "R" 1 "A" 26
 "R" 1 "A" 27
 "R" 1 "A" 28
 "R" 1 "A" 29
 "R" 1 "A" 30
```

```
 "R" 1 "A" 31
 "R" 1 "A" 32
 "R" 1 "A" 33
 "R" 1 "A" 34
 "R" 1 "A" 35
 "R" 1 "A" 36
 "R" 1 "A" 37
 "R" 1 "A" 38
 "R" 1 "A" 39
 "R" 1 "A" 40
 "R" 1 "A" 41
 "R" 1 "A" 42
 "R" 1 "A" 43
 "R" 1 "A" 44
!entry.SA.unit.name single str
 "SA"
!entry.SA.unit.positions table  dbl x  dbl y  dbl z
 9.152000 -1.077000 0.697000
 10.204000 -1.219000 0.564000
 8.914000 -0.040000 0.579000
 8.620000 -1.650000 -0.033000
 8.749000 -1.537000 2.100000
 8.998000 -2.571000 2.214000
 7.696000 -1.389000 2.217000
 9.476000 -0.727000 3.175000
 9.150000 0.290000 3.104000
 10.523000 -0.848000 2.988000
 9.235000 -1.205000 4.608000
 9.328000 -2.270000 4.640000
 8.253000 -0.901000 4.904000
 10.244000 -0.588000 5.579000
 10.085000 0.465000 5.680000
 11.258000 -0.696000 5.255000
 9.953000 -1.315000 6.894000
 10.536000 -2.210000 6.950000
 8.917000 -1.578000 6.950000
 10.325000 -0.382000 8.047000
 9.732000 0.507000 7.999000
 11.358000 -0.109000 7.982000
 10.054000 -1.114000 9.363000
 10.960000 -1.558000 9.719000
 9.324000 -1.880000 9.205000
 9.534000 -0.113000 10.397000
```

8.612000 0.297000 10.041000
10.285000 0.639000 10.520000
9.289000 -0.743000 11.770000
8.953000 -1.749000 11.629000
8.557000 -0.145000 12.271000
10.542000 -0.765000 12.648000
10.872000 0.244000 12.784000
11.259000 -1.375000 12.139000
10.340000 -1.392000 14.029000
9.875000 -0.666000 14.663000
11.308000 -1.686000 14.378000
9.472000 -2.651000 14.023000
10.007000 -3.387000 13.461000
8.542000 -2.295000 13.631000
9.061000 -3.309000 15.307000
8.616000 -4.240000 15.290000
9.271000 -2.689000 16.476000
9.747000 -1.773000 16.480000
!entry.SA.unit.residueconnect table int c1x int c2x int c3x int c4x int c5x int c6x
43 43 0 0 0 0
!entry.SA.unit.residues table str name int seq int childseq int startatomx str restype int imagingx
"SA" 1 45 1 "?" 0
!entry.SA.unit.residuesPdbSequenceNumber array int
1
!entry.SA.unit.solventcap array dbl
-1.000000
0.0
0.0
0.0
0.0
!entry.SA.unit.velocities table dbl x dbl y dbl z
0.0 0.0 0.0
0.0 0.0 0.0
0.0 0.0 0.0
0.0 0.0 0.0
0.0 0.0 0.0
0.0 0.0 0.0
0.0 0.0 0.0
0.0 0.0 0.0
0.0 0.0 0.0
0.0 0.0 0.0
0.0 0.0 0.0

```
 0.0 0.0 0.0
 0.0 0.0 0.0
 0.0 0.0 0.0
 0.0 0.0 0.0
 0.0 0.0 0.0
 0.0 0.0 0.0
 0.0 0.0 0.0
 0.0 0.0 0.0
 0.0 0.0 0.0
 0.0 0.0 0.0
 0.0 0.0 0.0
 0.0 0.0 0.0
 0.0 0.0 0.0
 0.0 0.0 0.0
 0.0 0.0 0.0
 0.0 0.0 0.0
 0.0 0.0 0.0
 0.0 0.0 0.0
 0.0 0.0 0.0
 0.0 0.0 0.0
 0.0 0.0 0.0
 0.0 0.0 0.0
 0.0 0.0 0.0
 0.0 0.0 0.0
 0.0 0.0 0.0
 0.0 0.0 0.0
 0.0 0.0 0.0
 0.0 0.0 0.0
 0.0 0.0 0.0
 0.0 0.0 0.0
 0.0 0.0 0.0
 0.0 0.0 0.0
 0.0 0.0 0.0
!entry.SPM.unit.atoms table  str name  str type  int typex  int resx  int flags  int seq
int elmnt  dbl chg
 "C2" "cA" 0 1 131075 1 6 0.012739
 "HX" "hE" 0 1 131075 2 1 0.087455
 "C3" "cA" 0 1 131075 3 6 0.176625
 "HA" "hE" 0 1 131075 4 1 0.051622
 "HB" "hE" 0 1 131075 5 1 0.051622
 "O31" "oT" 0 1 131075 6 8 -0.533356
 "P31" "pA" 0 1 131075 7 15 1.324762
 "O32" "oT" 0 1 131075 8 8 -0.459308
```

```
 "C31" "cA" 0 1 131075 9 6 0.282447
 "H1A" "hE" 0 1 131075 10 1 0.049886
 "H1B" "hE" 0 1 131075 11 1 0.049886
 "C32" "cA" 0 1 131075 12 6 -0.493665
 "H2A" "hX" 0 1 131075 13 1 0.233840
 "H2B" "hX" 0 1 131075 14 1 0.233840
 "N31" "nA" 0 1 131075 15 7 0.245275
 "C33" "cA" 0 1 131075 16 6 -0.386611
 "H3A" "hX" 0 1 131075 17 1 0.191569
 "H3B" "hX" 0 1 131075 18 1 0.191569
 "H3C" "hX" 0 1 131075 19 1 0.191569
 "C34" "cA" 0 1 131075 20 6 -0.386611
 "H4A" "hX" 0 1 131075 21 1 0.191569
 "H4B" "hX" 0 1 131075 22 1 0.191569
 "H4C" "hX" 0 1 131075 23 1 0.191569
 "C35" "cA" 0 1 131075 24 6 -0.386611
 "H5A" "hX" 0 1 131075 25 1 0.191569
 "H5B" "hX" 0 1 131075 26 1 0.191569
 "H5C" "hX" 0 1 131075 27 1 0.191569
 "O33" "oP" 0 1 131075 28 8 -0.884789
 "O34" "oP" 0 1 131075 29 8 -0.884789
 "N11" "nN" 0 1 131075 30 7 -0.388410
 "H11" "hN" 0 1 131075 31 1 0.273592
 "C11" "cC" 0 1 131075 32 6 0.617231
 "O12" "oC" 0 1 131075 33 8 -0.645303
 "C1" "cA" 0 1 131075 34 6 0.323092
 "HR" "hE" 0 1 131075 35 1 0.069208
 "O22" "oH" 0 1 131075 36 8 -0.723033
 "H22" "hO" 0 1 131075 37 1 0.434964
!entry.SPM.unit.atomspertinfo table  str pname  str ptype  int ptypex  int pelmnt  dbl pchg
 "C2" "cA" 0 -1 0.0
 "HX" "hE" 0 -1 0.0
 "C3" "cA" 0 -1 0.0
 "HA" "hE" 0 -1 0.0
 "HB" "hE" 0 -1 0.0
 "O31" "oT" 0 -1 0.0
 "P31" "pA" 0 -1 0.0
 "O32" "oT" 0 -1 0.0
 "C31" "cA" 0 -1 0.0
 "H1A" "hE" 0 -1 0.0
 "H1B" "hE" 0 -1 0.0
 "C32" "cA" 0 -1 0.0
 "H2A" "hX" 0 -1 0.0
```

```
 "H2B" "hX" 0 -1 0.0
 "N31" "nA" 0 -1 0.0
 "C33" "cA" 0 -1 0.0
 "H3A" "hX" 0 -1 0.0
 "H3B" "hX" 0 -1 0.0
 "H3C" "hX" 0 -1 0.0
 "C34" "cA" 0 -1 0.0
 "H4A" "hX" 0 -1 0.0
 "H4B" "hX" 0 -1 0.0
 "H4C" "hX" 0 -1 0.0
 "C35" "cA" 0 -1 0.0
 "H5A" "hX" 0 -1 0.0
 "H5B" "hX" 0 -1 0.0
 "H5C" "hX" 0 -1 0.0
 "O33" "oP" 0 -1 0.0
 "O34" "oP" 0 -1 0.0
 "N11" "nN" 0 -1 0.0
 "H11" "hN" 0 -1 0.0
 "C11" "cC" 0 -1 0.0
 "O12" "oC" 0 -1 0.0
 "C1" "cA" 0 -1 0.0
 "HR" "hE" 0 -1 0.0
 "O22" "oH" 0 -1 0.0
 "H22" "hO" 0 -1 0.0
!entry.SPM.unit.boundbox array dbl
 -1.000000
 0.0
 0.0
 0.0
 0.0
!entry.SPM.unit.childsequence single int
 2
!entry.SPM.unit.connect array int
 32
 34
!entry.SPM.unit.connectivity table  int atom1x  int atom2x  int flags
 1 30 1
 1 3 1
 1 2 1
 1 34 1
 3 6 1
 3 5 1
 3 4 1
```

```
 6 7 1
 7 8 1
 7 28 1
 7 29 1
 8 9 1
 9 12 1
 9 11 1
 9 10 1
 12 15 1
 12 13 1
 12 14 1
 15 20 1
 15 24 1
 15 16 1
 16 17 1
 16 18 1
 16 19 1
 20 23 1
 20 22 1
 20 21 1
 24 27 1
 24 25 1
 24 26 1
 30 32 1
 30 31 1
 32 33 1
 34 35 1
 34 36 1
 36 37 1
!entry.SPM.unit.hierarchy table  str abovetype  int abovex  str belowtype  int belowx
 "U" 0 "R" 1
 "R" 1 "A" 1
 "R" 1 "A" 2
 "R" 1 "A" 3
 "R" 1 "A" 4
 "R" 1 "A" 5
 "R" 1 "A" 6
 "R" 1 "A" 7
 "R" 1 "A" 8
 "R" 1 "A" 9
 "R" 1 "A" 10
 "R" 1 "A" 11
 "R" 1 "A" 12
```

```
 "R" 1 "A" 13
 "R" 1 "A" 14
 "R" 1 "A" 15
 "R" 1 "A" 16
 "R" 1 "A" 17
 "R" 1 "A" 18
 "R" 1 "A" 19
 "R" 1 "A" 20
 "R" 1 "A" 21
 "R" 1 "A" 22
 "R" 1 "A" 23
 "R" 1 "A" 24
 "R" 1 "A" 25
 "R" 1 "A" 26
 "R" 1 "A" 27
 "R" 1 "A" 28
 "R" 1 "A" 29
 "R" 1 "A" 30
 "R" 1 "A" 31
 "R" 1 "A" 32
 "R" 1 "A" 33
 "R" 1 "A" 34
 "R" 1 "A" 35
 "R" 1 "A" 36
 "R" 1 "A" 37
!entry.SPM.unit.name single str
 "SPM"
!entry.SPM.unit.positions table  dbl x  dbl y  dbl z
 -41.583000 54.249000 135.723000
 -42.533000 53.708000 135.697000
 -40.999000 53.884000 137.051000
 -40.811000 52.812000 137.148000
 -41.752000 54.112000 137.810000
 -39.815000 54.716000 137.276000
 -39.293000 55.046000 138.706000
 -38.408000 53.823000 139.126000
 -39.007000 52.753000 139.780000
 -39.703000 52.245000 139.107000
 -39.513000 53.055000 140.700000
 -37.924000 51.780000 140.007000
 -37.321000 51.694000 139.101000
 -38.386000 50.810000 140.197000
 -36.964000 52.047000 141.081000
```

```
 -36.033000 53.154000 140.832000
 -35.716000 52.950000 139.809000
 -36.582000 54.084000 140.986000
 -35.184000 53.174000 141.517000
 -37.627000 52.236000 142.409000
 -36.897000 52.122000 143.212000
 -37.859000 53.302000 142.403000
 -38.452000 51.526000 142.332000
 -36.268000 50.708000 141.208000
 -35.199000 50.867000 141.358000
 -36.826000 50.065000 141.889000
 -36.280000 50.262000 140.212000
 -40.354000 55.264000 139.710000
 -38.275000 56.136000 138.639000
 -40.692000 54.026000 134.611000
 -39.968000 54.718000 134.492000
 -40.660000 52.864000 133.849000
 -41.427000 51.930000 133.938000
 -42.202000 55.632000 135.867000
 -41.432000 56.273000 136.304000
 -43.465000 55.672000 136.573000
 -44.064000 56.048000 135.903000
!entry.SPM.unit.residueconnect table  int c1x  int c2x  int c3x  int c4x  int c5x  int c6x
 0 0 0 0 0 0
!entry.SPM.unit.residues table  str name  int seq  int childseq  int startatomx  str
restype  int imagingx
 "SPM" 5 38 1 "?" 0
!entry.SPM.unit.residuesPdbSequenceNumber array int
 1
!entry.SPM.unit.solventcap array dbl
 -1.000000
 0.0
 0.0
 0.0
 0.0
!entry.SPM.unit.velocities table  dbl x  dbl y  dbl z
 0.0 0.0 0.0
 0.0 0.0 0.0
 0.0 0.0 0.0
 0.0 0.0 0.0
 0.0 0.0 0.0
 0.0 0.0 0.0
 0.0 0.0 0.0
```

```
 0.0 0.0 0.0
 0.0 0.0 0.0
 0.0 0.0 0.0
 0.0 0.0 0.0
 0.0 0.0 0.0
 0.0 0.0 0.0
 0.0 0.0 0.0
 0.0 0.0 0.0
 0.0 0.0 0.0
 0.0 0.0 0.0
 0.0 0.0 0.0
 0.0 0.0 0.0
 0.0 0.0 0.0
 0.0 0.0 0.0
 0.0 0.0 0.0
 0.0 0.0 0.0
 0.0 0.0 0.0
 0.0 0.0 0.0
 0.0 0.0 0.0
 0.0 0.0 0.0
 0.0 0.0 0.0
 0.0 0.0 0.0
 0.0 0.0 0.0
 0.0 0.0 0.0
 0.0 0.0 0.0
 0.0 0.0 0.0
 0.0 0.0 0.0
 0.0 0.0 0.0
 0.0 0.0 0.0
 0.0 0.0 0.0
!entry.ST.unit.atoms table  str name  str type  int typex  int resx  int flags  int seq
int elmnt  dbl chg
 "C118" "cD" 0 1 131073 1 6 -0.120069
 "H18R" "hL" 0 1 131073 2 1 0.027938
 "H18S" "hL" 0 1 131073 3 1 0.027938
 "H18T" "hL" 0 1 131073 4 1 0.027938
 "C117" "cD" 0 1 131073 5 6 0.020665
 "H17R" "hL" 0 1 131073 6 1 0.008483
 "H17S" "hL" 0 1 131073 7 1 0.008483
 "C116" "cD" 0 1 131073 8 6 -0.027654
 "H16R" "hL" 0 1 131073 9 1 0.015627
 "H16S" "hL" 0 1 131073 10 1 0.015627
 "C115" "cD" 0 1 131073 11 6 -0.032444
```

"H15R" "hL" 0 1 131073 12 1 0.010253
"H15S" "hL" 0 1 131073 13 1 0.010253
"C114" "cD" 0 1 131073 14 6 -0.013489
"H14R" "hL" 0 1 131073 15 1 0.010073
"H14S" "hL" 0 1 131073 16 1 0.010073
"C113" "cD" 0 1 131073 17 6 -0.011590
"H13R" "hL" 0 1 131073 18 1 0.006022
"H13S" "hL" 0 1 131073 19 1 0.006022
"C112" "cD" 0 1 131073 20 6 -0.019153
"H12R" "hL" 0 1 131073 21 1 0.008198
"H12S" "hL" 0 1 131073 22 1 0.008198
"C111" "cD" 0 1 131073 23 6 -0.017093
"H11R" "hL" 0 1 131073 24 1 0.010206
"H11S" "hL" 0 1 131073 25 1 0.010206
"C110" "cD" 0 1 131073 26 6 -0.026418
"H10R" "hL" 0 1 131073 27 1 0.010825
"H10S" "hL" 0 1 131073 28 1 0.010825
"C19" "cD" 0 1 131073 29 6 -0.017997
"H9R" "hL" 0 1 131073 30 1 0.009595
"H9S" "hL" 0 1 131073 31 1 0.009595
"C18" "cD" 0 1 131073 32 6 -0.014623
"H8R" "hL" 0 1 131073 33 1 0.007326
"H8S" "hL" 0 1 131073 34 1 0.007326
"C17" "cD" 0 1 131073 35 6 -0.019469
"H7R" "hL" 0 1 131073 36 1 0.009292
"H7S" "hL" 0 1 131073 37 1 0.009292
"C16" "cD" 0 1 131073 38 6 -0.037627
"H6R" "hL" 0 1 131073 39 1 0.017740
"H6S" "hL" 0 1 131073 40 1 0.017740
"C15" "cD" 0 1 131073 41 6 -0.013443
"H5R" "hL" 0 1 131073 42 1 0.008191
"H5S" "hL" 0 1 131073 43 1 0.008191
"C14" "cD" 0 1 131073 44 6 -0.059792
"H4R" "hL" 0 1 131073 45 1 0.024241
"H4S" "hL" 0 1 131073 46 1 0.024241
"C13" "cD" 0 1 131073 47 6 -0.000128
"H3R" "hL" 0 1 131073 48 1 0.013886
"H3S" "hL" 0 1 131073 49 1 0.013886
"C12" "cD" 0 1 131073 50 6 -0.117020
"H2R" "hL" 0 1 131073 51 1 0.051807
"H2S" "hL" 0 1 131073 52 1 0.051807
!entry.ST.unit.atomspertinfo table str pname str ptype int ptypex int pelmnt dbl pchg
"C118" "cD" 0 -1 0.0

```
"H18R" "hL" 0 -1 0.0
"H18S" "hL" 0 -1 0.0
"H18T" "hL" 0 -1 0.0
"C117" "cD" 0 -1 0.0
"H17R" "hL" 0 -1 0.0
"H17S" "hL" 0 -1 0.0
"C116" "cD" 0 -1 0.0
"H16R" "hL" 0 -1 0.0
"H16S" "hL" 0 -1 0.0
"C115" "cD" 0 -1 0.0
"H15R" "hL" 0 -1 0.0
"H15S" "hL" 0 -1 0.0
"C114" "cD" 0 -1 0.0
"H14R" "hL" 0 -1 0.0
"H14S" "hL" 0 -1 0.0
"C113" "cD" 0 -1 0.0
"H13R" "hL" 0 -1 0.0
"H13S" "hL" 0 -1 0.0
"C112" "cD" 0 -1 0.0
"H12R" "hL" 0 -1 0.0
"H12S" "hL" 0 -1 0.0
"C111" "cD" 0 -1 0.0
"H11R" "hL" 0 -1 0.0
"H11S" "hL" 0 -1 0.0
"C110" "cD" 0 -1 0.0
"H10R" "hL" 0 -1 0.0
"H10S" "hL" 0 -1 0.0
"C19" "cD" 0 -1 0.0
"H9R" "hL" 0 -1 0.0
"H9S" "hL" 0 -1 0.0
"C18" "cD" 0 -1 0.0
"H8R" "hL" 0 -1 0.0
"H8S" "hL" 0 -1 0.0
"C17" "cD" 0 -1 0.0
"H7R" "hL" 0 -1 0.0
"H7S" "hL" 0 -1 0.0
"C16" "cD" 0 -1 0.0
"H6R" "hL" 0 -1 0.0
"H6S" "hL" 0 -1 0.0
"C15" "cD" 0 -1 0.0
"H5R" "hL" 0 -1 0.0
"H5S" "hL" 0 -1 0.0
"C14" "cD" 0 -1 0.0
```

```
 "H4R" "hL" 0 -1 0.0
 "H4S" "hL" 0 -1 0.0
 "C13" "cD" 0 -1 0.0
 "H3R" "hL" 0 -1 0.0
 "H3S" "hL" 0 -1 0.0
 "C12" "cD" 0 -1 0.0
 "H2R" "hL" 0 -1 0.0
 "H2S" "hL" 0 -1 0.0
!entry.ST.unit.boundbox array dbl
 -1.000000
 0.0
 0.0
 0.0
 0.0
!entry.ST.unit.childsequence single int
 2
!entry.ST.unit.connect array int
 50
 50
!entry.ST.unit.connectivity table  int atom1x  int atom2x  int flags
 1 5 1
 1 4 1
 1 3 1
 1 2 1
 5 8 1
 5 7 1
 5 6 1
 8 11 1
 8 9 1
 8 10 1
 11 14 1
 11 13 1
 11 12 1
 14 17 1
 14 15 1
 14 16 1
 17 20 1
 17 19 1
 17 18 1
 20 23 1
 20 21 1
 20 22 1
 23 26 1
```

```
 23 25 1
 23 24 1
 26 29 1
 26 27 1
 26 28 1
 29 32 1
 29 31 1
 29 30 1
 32 35 1
 32 33 1
 32 34 1
 35 38 1
 35 37 1
 35 36 1
 38 41 1
 38 39 1
 38 40 1
 41 44 1
 41 43 1
 41 42 1
 44 47 1
 44 45 1
 44 46 1
 47 50 1
 47 49 1
 47 48 1
 50 51 1
 50 52 1
!entry.ST.unit.hierarchy table  str abovetype  int abovex  str belowtype  int belowx
 "U" 0 "R" 1
 "R" 1 "A" 1
 "R" 1 "A" 2
 "R" 1 "A" 3
 "R" 1 "A" 4
 "R" 1 "A" 5
 "R" 1 "A" 6
 "R" 1 "A" 7
 "R" 1 "A" 8
 "R" 1 "A" 9
 "R" 1 "A" 10
 "R" 1 "A" 11
 "R" 1 "A" 12
 "R" 1 "A" 13
```

```
 "R" 1 "A" 14
 "R" 1 "A" 15
 "R" 1 "A" 16
 "R" 1 "A" 17
 "R" 1 "A" 18
 "R" 1 "A" 19
 "R" 1 "A" 20
 "R" 1 "A" 21
 "R" 1 "A" 22
 "R" 1 "A" 23
 "R" 1 "A" 24
 "R" 1 "A" 25
 "R" 1 "A" 26
 "R" 1 "A" 27
 "R" 1 "A" 28
 "R" 1 "A" 29
 "R" 1 "A" 30
 "R" 1 "A" 31
 "R" 1 "A" 32
 "R" 1 "A" 33
 "R" 1 "A" 34
 "R" 1 "A" 35
 "R" 1 "A" 36
 "R" 1 "A" 37
 "R" 1 "A" 38
 "R" 1 "A" 39
 "R" 1 "A" 40
 "R" 1 "A" 41
 "R" 1 "A" 42
 "R" 1 "A" 43
 "R" 1 "A" 44
 "R" 1 "A" 45
 "R" 1 "A" 46
 "R" 1 "A" 47
 "R" 1 "A" 48
 "R" 1 "A" 49
 "R" 1 "A" 50
 "R" 1 "A" 51
 "R" 1 "A" 52
!entry.ST.unit.name single str
 "ST"
!entry.ST.unit.positions table  dbl x  dbl y  dbl z
 12.346000 0.172000 0.239000
```

13.222000 0.143000 -0.403000
12.424000 -0.652000 0.943000
12.385000 1.096000 0.809000
11.057000 0.081000 -0.577000
11.064000 -0.836000 -1.161000
11.026000 0.899000 -1.294000
9.797000 0.120000 0.290000
9.792000 1.038000 0.875000
9.831000 -0.698000 1.008000
8.502000 0.029000 -0.520000
8.507000 -0.889000 -1.105000
8.469000 0.847000 -1.238000
7.242000 0.068000 0.348000
7.236000 0.986000 0.933000
7.275000 -0.750000 1.066000
5.947000 -0.023000 -0.462000
5.952000 -0.941000 -1.047000
5.914000 0.795000 -1.180000
4.687000 0.016000 0.405000
4.681000 0.934000 0.990000
4.720000 -0.802000 1.123000
3.392000 -0.075000 -0.404000
3.397000 -0.993000 -0.989000
3.359000 0.743000 -1.122000
2.132000 -0.036000 0.463000
2.126000 0.882000 1.048000
2.165000 -0.854000 1.181000
0.837000 -0.127000 -0.347000
0.842000 -1.045000 -0.932000
0.804000 0.691000 -1.065000
-0.423000 -0.089000 0.521000
-0.429000 0.830000 1.106000
-0.390000 -0.906000 1.239000
-1.718000 -0.180000 -0.289000
-1.712000 -1.098000 -0.874000
-1.751000 0.638000 -1.007000
-2.977000 -0.141000 0.578000
-2.984000 0.777000 1.163000
-2.944000 -0.959000 1.297000
-4.272000 -0.234000 -0.232000
-4.267000 -1.152000 -0.815000
-4.306000 0.584000 -0.950000
-5.531000 -0.196000 0.636000

```
 -5.539000 0.724000 1.220000
 -5.498000 -1.012000 1.355000
 -6.822000 -0.294000 -0.177000
 -6.828000 -1.215000 -0.752000
 -6.861000 0.522000 -0.896000
 -8.079000 -0.265000 0.706000
 -8.110000 0.652000 1.284000
 -8.050000 -1.094000 1.403000
!entry.ST.unit.residueconnect table  int c1x  int c2x  int c3x  int c4x  int c5x  int c6x
 50 50 0 0 0 0
!entry.ST.unit.residues table  str name  int seq  int childseq  int startatomx  str restype
int imagingx
 "ST" 1 54 1 "?" 0
!entry.ST.unit.residuesPdbSequenceNumber array int
 1
!entry.ST.unit.solventcap array dbl
 -1.000000
 0.0
 0.0
 0.0
 0.0
!entry.ST.unit.velocities table  dbl x  dbl y  dbl z
 0.0 0.0 0.0
 0.0 0.0 0.0
 0.0 0.0 0.0
 0.0 0.0 0.0
 0.0 0.0 0.0
 0.0 0.0 0.0
 0.0 0.0 0.0
 0.0 0.0 0.0
 0.0 0.0 0.0
 0.0 0.0 0.0
 0.0 0.0 0.0
 0.0 0.0 0.0
 0.0 0.0 0.0
 0.0 0.0 0.0
 0.0 0.0 0.0
 0.0 0.0 0.0
 0.0 0.0 0.0
 0.0 0.0 0.0
 0.0 0.0 0.0
 0.0 0.0 0.0
 0.0 0.0 0.0
```

```
0.0 0.0 0.0
0.0 0.0 0.0
0.0 0.0 0.0
0.0 0.0 0.0
0.0 0.0 0.0
0.0 0.0 0.0
0.0 0.0 0.0
0.0 0.0 0.0
0.0 0.0 0.0
0.0 0.0 0.0
0.0 0.0 0.0
0.0 0.0 0.0
0.0 0.0 0.0
0.0 0.0 0.0
0.0 0.0 0.0
0.0 0.0 0.0
0.0 0.0 0.0
0.0 0.0 0.0
0.0 0.0 0.0
0.0 0.0 0.0
0.0 0.0 0.0
0.0 0.0 0.0
0.0 0.0 0.0
0.0 0.0 0.0
0.0 0.0 0.0
0.0 0.0 0.0
0.0 0.0 0.0
0.0 0.0 0.0
0.0 0.0 0.0
0.0 0.0 0.0
0.0 0.0 0.0
```

REFERENCES

1. Skjevik, Å. A.; Madej, B. D.; Walker, R. C.; Teigen, K., LIPID11: A Modular Framework for Lipid Simulations Using Amber. *J. Phys. Chem. B* **2012,** *116* (36), 11124-11136.
